# Double Catalytic Activity Unveiled: Synthesis, Characterization, and Catalytic Applications of Iridium Complexes in Transfer Hydrogenation and Photomediated Transformations

**DOI:** 10.1021/acscatal.4c00673

**Published:** 2024-04-11

**Authors:** Laura Blanco, Andrea Uroz, Kevin Gutiérrez, Silvia Cabrera, Alba Collado, José Alemán

**Affiliations:** ‡ Inorganic Chemistry Department, Sciences Faculty, Universidad Autónoma de Madrid, Madrid 28049, Spain; † Organic Chemistry Department, Sciences Faculty, Universidad Autónoma de Madrid, Madrid 28049, Spain; § Institute for Advanced Research in Chemical Sciences (IAdChem), Sciences Faculty, Universidad Autónoma de Madrid, Madrid 28049, Spain

**Keywords:** catalysis, iridium, photocatalysis, transfer hydrogenation, tandem catalysis

## Abstract

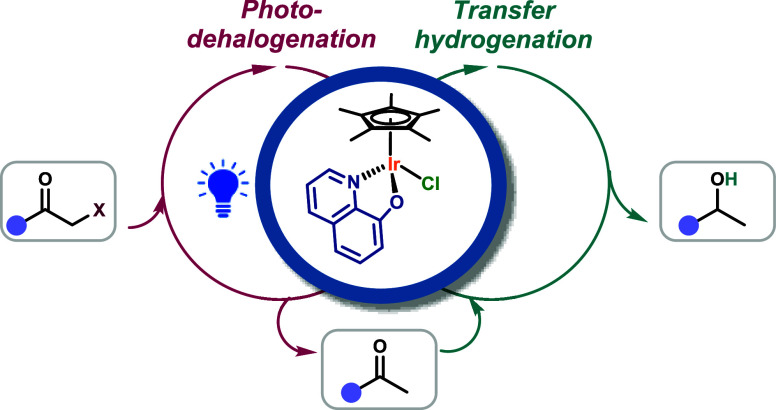

Iridium complexes have been demonstrated to be highly active catalysts for a wide variety of transformations. Their unique photophysical and photochemical properties render them as one of the most established photocatalysts. Moreover, iridium complexes are widely acknowledged for their efficiency in transfer hydrogenation reactions. However, the development of iridium complexes able to promote both traditional organometallic catalysis and photocatalysis is scarce. Thus, the design of iridium-based catalysts is still an active area of research. In this context, we targeted the synthesis of a family of Ir-Cp* systems to explore their (photo)catalytic applications. Here, we describe the synthesis, structural characterization, and photophysical properties of iridium complexes of formula [IrCp*Cl(N^O)]. These complexes have been applied with a double catalytic function, in transfer hydrogenation for carbonyl reduction and in different photomediated transformations.

## Introduction

The use of iridium complexes as photocatalysts has emerged as a highly promising area of research, with significant implications in various scientific disciplines. Iridium complexes exhibit unique photophysical and photochemical properties that render them well-suited for harnessing light energy, thereby promoting a wide array of catalytic processes.^1,2^ Therefore, iridium complexes can absorb visible light and undergo excited-state transformations to trigger a range of chemical reactions. These reactions encompass a broad spectrum of organic transformations, including but not limited to C–C bond formation, C–H activation, C–X bond reduction reactions, and redox reactions among others.^1,2^ The exceptional photophysical properties of iridium complexes, such as their long-lived excited states and high quantum yields, enable efficient energy and single-electron transfer processes to generate reactive intermediates for subsequent transformations.

**Figure 1:**
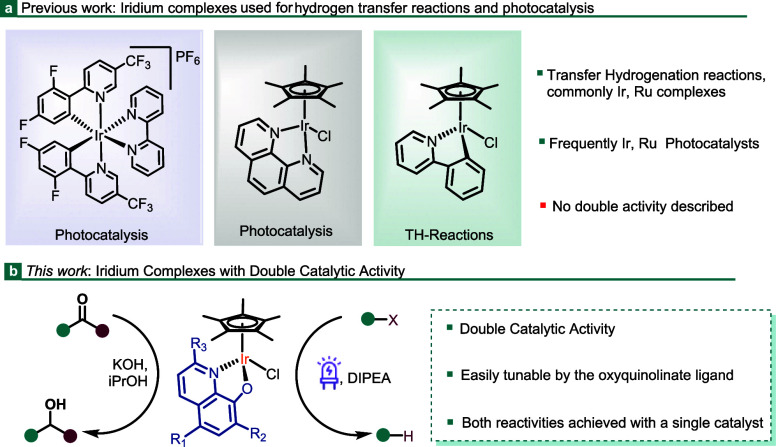
Iridium complexes for (a) hydrogen transfer reactions, photocatalysis, and (b) our concept.

The most common Ir-based photocatalysts are octahedral Ir(III)complexes containing *N*-heterocyclic bidentate ligands, including the well-known *fac-*[Ir(ppy)_3_], [Ir(ppy)_2_(dtbbpy)]^+^, and derivatives (left, Figure 1a).^1^ These complexes are outstanding photocatalysts, and their use is currently widespread in the field. However, their high stability makes them inert toward ligand substitution, preventing their use as traditional organometallic catalysts. In this area, piano-stool Ir(III)Cp*-based compounds are a very common family of catalysts. These complexes, known since the 70s,^3^ have attracted much attention and have found a tremendous number of applications in catalysis^3−8^ due to their straightforward synthesis and high versatility. The complexes' catalytic activity heavily depends on the nature of the ligands completing the metal coordination sphere, and therefore, they offer great opportunities for catalyst design by careful ligand choice. A particularly interesting transformation, typically promoted by Ir complexes, is the transfer hydrogenation (TH) of unsaturated substrates (right, Figure 1a).^7,9,10^ TH plays a crucial role in the pharmaceutical, agrochemical, and fine chemical industries, serving as a fundamental transformation. TH is widely recognized as a key method for conveniently obtaining valuable molecules with enhanced properties. This reaction involves the addition of hydrogen to an unsaturated molecule using a non-H_2_ gaseous source, eliminating the need for hazardous reagents.^7^ Consequently, TH has emerged as an appealing alternative to direct hydrogenation. In recent years, it has found frequent applications in the gentle production of alcohols or amines by reacting carbonyl compounds and imines with readily available, inexpensive hydrogen donors.^7^

Interestingly, despite the high number of IrCp*-based complexes reported in the literature and their wide range of catalytic applications, their use in photocatalysis is an underexplored field. Indeed, only two examples of IrCp*-promoted photocatalytic hydrogenation^11^ and dehydrogenation reactions have been very recently reported (middle, Figure 1a).^12^ Therefore, the full potential of IrCp*-based species in photocatalysis has yet to be explored. The development of IrCp* photocatalysts is highly desirable since it would open the door to highly versatile catalysts able to promote both photocatalysis and traditional organometallic catalysis. To the best of our knowledge, the combination of photocatalysis and a catalytic organometallic transformation in a tandem process employing a single catalyst has not been described.^13^

In this context and in our search for new and more efficient (photo)catalytic transformations,^14−19^ we set two objectives: first, we sought to explore the photocatalytic potential of IrCp*-based complexes, studying the ligand influence on the photocatalytic activity; second, exploring the combination of photocatalytic and traditional organometallic catalysis employing a single catalyst. To develop this concept, we focused on hydroxyquinolines as proligands. Hydroxyquinolines are commercially available and offer a wide range of substitutions. We have previously reported that oxyquinolinates are excellent bidentate ligands to modulate the photocatalytic activity of Pt complexes.^16,19^ Additionally, [IrCp*Cl(N^O)] complexes have been employed as organometallic catalysts in chemoselective transformations, where the selectivity was controlled by the ligand nature.^20−24^ However, these complexes have not been employed as photocatalysts or in transfer hydrogenation reactions. In this context, we aimed to synthesize iridium complexes with Cp* and 8-oxyquinolinate ligands capable of catalyzing both carbonyl reduction (TH reactions) and photomediated processes like halogen reduction and alkene isomerization (right, Figure 1b).

Thus, in this work, we present our efforts in the synthesis of iridium complexes, their photophysical properties, and double catalytic activity in a range of transformations. This includes the reduction of ketones through TH reactions as well as the reduction of carbon–halogen bonds and isomerization of double bonds under photocatalysis. Finally, as a proof of concept, the ability of the [IrCp*Cl(N^O)] complexes to catalyze two independent reactions in a sequential manner as a single catalyst will be evaluated in the photoreductive debromination/transfer hydrogenation process.

## Results and Discussion

### Synthesis and Characterization of the [IrCp*Cl(N^O)] Complexes

**Scheme 1:**
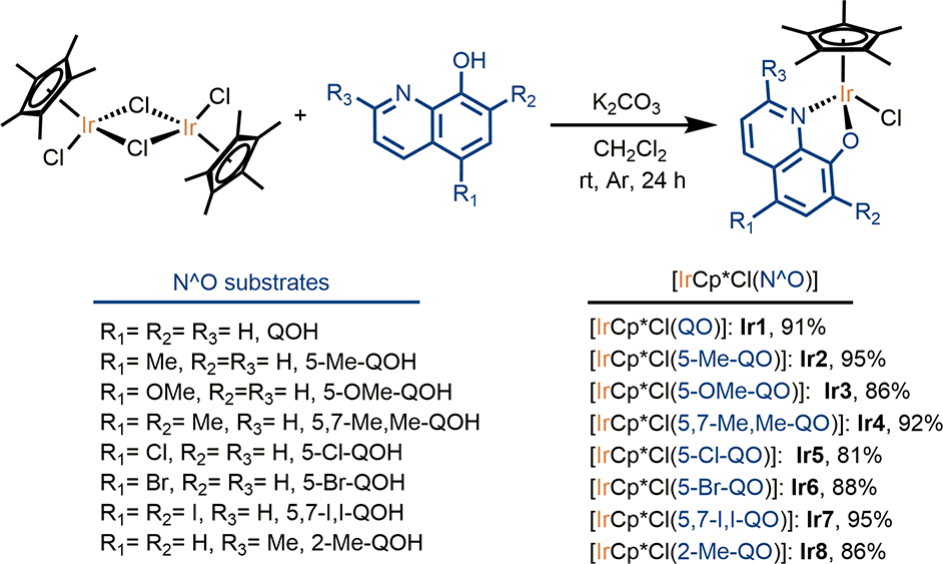
Synthesis of Complexes [IrCp*Cl(N^O)]

A family of iridium-8-oxyquinolinate complexes has been synthesized. In order to explore the electronic and steric influences of the 8-oxyquinolinate ligand on the catalytic activity of the complexes, diverse 8-hydroxyquinolines bearing different substituents, such as halogens, alkyls, and alkoxides, were selected. [IrCp*Cl(N^O)] complexes (**Ir1**–**Ir8**) were prepared by reacting the dinuclear [IrCp*Cl_2_]_2_ complex with the corresponding 8-hydroxyquinoline in the presence of K_2_CO_3_ as a base at rt for 24 h (Scheme 1).^21−24^ The corresponding pure complexes **Ir1**–**Ir8** were obtained as solids in high yields (81–95%) after filtration of the reaction mixture through Celite, concentration of the filtrate, and subsequent precipitation by addition of *n*-pentane.

All Ir complexes were characterized by ^1^H NMR spectroscopy, and the spectroscopic data were compared to those of the reported structures.^21−24^ The new [IrCp*Cl(N^O)] complexes, **Ir2**, **Ir4**, and **Ir8**, were characterized by ^1^H NMR, ^13^C{^1^H} NMR, and IR spectroscopy, HRMS spectrometry, and elemental analysis. The data matched the proposed structure shown in Scheme 1. The signal corresponding to the chemically equivalent CH_3_ groups of the Cp* ligand appeared as a singlet in the 1.69–1.72 ppm range in the ^1^H NMR spectra in CDCl_3_ or CD_2_Cl_2_ for all [IrCp*Cl(N^O)] complexes.^25^ This signal is downfield shifted with respect to that of the dinuclear [IrCp*Cl_2_]_2_ species, which resonates at 1.59 ppm.^26^ The signals corresponding to the 8-oxyquinolinate protons are shifted upfield compared to the hydroxyquinoline substrates (see the SI).

**Figure 2:**
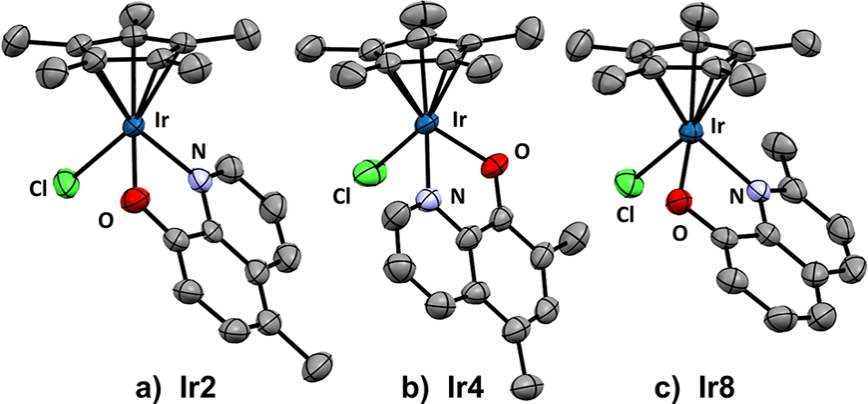
Structural views of complexes **Ir2** (a), **Ir4** (b), and **Ir8** (c). Ellipsoids are shown at a 50% level, and hydrogen atoms are omitted for clarity. Only one enantiomer of the complex is shown.

To unambiguously determine the structure of the new complexes **Ir2**, **Ir4**, and **Ir8**, single crystals were grown by the slow diffusion of *n*-pentane into saturated dichloromethane solutions of the corresponding complex. The obtained crystals were suitable for X-ray diffraction analysis, and the structures obtained are shown in Figure 2. Table 1 outlines the most relevant crystallographic data of **Ir2**, **Ir4**, and **Ir8** and those reported for **Ir1**(27) for comparison purposes. Complexes **Ir2**, **Ir4**, and **Ir8** show the expected three-legged piano-stool geometry. A η^5^-Cp*, a Cl, and the bidentate *O*-,*N*-oxyquinolinate ligands complete the coordination sphere around the Ir center. Complexes **Ir2**, **Ir4**, and **Ir8** were obtained as a racemic mixture, and both enantiomers were observed in the crystal structure. The bond distances (Å) and angles (deg) in **Ir2**, **Ir4**, and **Ir8** lie in the same range of those previously obtained for **Ir1**. The five C atoms of the Cp* moiety are in the same plane, and the distances and angles have been measured with respect to the ring centroid. The Ir-Cp* distances in **Ir2**, **Ir4**, and **Ir8** are within the same range (Table 2, entry 4) and are slightly shorter than that of **Ir1**. In contrast, the distances between the Ir center and the N, O, and Cl donor atoms in **Ir2**, **Ir4**, and **Ir8** are slightly longer than the related distances in **Ir1** (Table 2, entries 1–3). Due to the bite angle of the bidentate ligand, the O–Ir–N angles are *ca*. 78°, whereas the O–Ir–Cl and N–Ir–Cl angles are greater (84–89°). These values are similar to those found in **Ir1** (Table 1).

**Table 1:** Selected Bond Lengths (Å) and Angles (^o^) for Ir(III) Complexes **Ir2**, **Ir4**, **Ir8**, and **Ir1**^a^

entry	bond lengths	**Ir2**	**Ir4**	**Ir8**	**Ir1**
1	Ir–N	2.090(7)	2.118(12)	2.117(3)	2.088(7)
2	Ir–O	2.116(6)	2.095(10)	2.103(3)	2.091(6)
3	Ir–Cl	2.396(2)	2.392(4)	2.410(1)	2.386(2)
4	Ir-Cp* centroid	1.772	1.774	1.776	1.788(4)

entry	angles	**Ir2**	**Ir4**	**Ir8**	**Ir1**
5	O–Ir–N	78.3(3)	78.6(4)	78.3(1)	77.8(3)
6	O–Ir–Cl	87.30(19)	84.5(3)	88.81(8)	84.6(2)
7	N–Ir–Cl	84.60(19)	86.8(3)	86.22(9)	85.2(2)
8	centroid Cp*–Ir–Cl	128.33	126.35	128.21	126.80
9	centroid Cp*–Ir–O	127.76	129.67	124.31	131.25
10	centroid Cp*–Ir–N	133.29	133.54	134.36	133.15

a The crystallographic information for **Ir1** has been retrieved from the CCDC database^27^ and has been included for comparison purposes.

**Table 2:** UV–Vis Absorption and Emission Data for Complexes **Ir1**–**Ir8**

		absorption, 298 K^a^	emission, 298 K^b^		emission, 77 K^d^		
entry	Ir complex	λ_abs_/nm (ε/L·mol^–1^·cm^–1^)	λ_em_/nm	τ (ns)^c^	λ_em_/nm	*E*_0–0_/eV^e^	*E*_T1_/eV^f^
1	**Ir1** [IrCp*Cl(QO)]	259 (20973), 349 (3835), 430 (3152)	675	131.9	619	2.24	2.00
2	**Ir2** [IrCp*Cl(5-Me-QO)]	263 (30556), 357 (6208), 471 (5452)	705	587.2	635	2.10	1.94
3	**Ir3** [IrCp*Cl(5-OMe-QO)]	265 (19864), 368 (5041), 485 (3135)	-g	-g	-g	-	-
4	**Ir4** [IrCp*Cl(5,7-Me,Me-QO)]	264 (20207), 358 (4460), 475 (3582)	736	498.7	609	2.05	2.03
5	**Ir5** [IrCp*Cl(5-Cl-QO)]	262 (22968), 359 (4238), 468 (3394)	696	496.2	644	2.11	1.92
6	**Ir6** [IrCp*Cl(5-Br-QO)]	265 (28387), 358 (4225), 465 (4224)	696	381.4	642	2.14	1.93
7	**Ir7** [IrCp*Cl(5,7-I,I-QO)]	277 (22048), 365 (5338), 466 (4467)	684	164.3	639	2.16	1.94
8	**Ir8** [IrCp*Cl(2-Me-QO)]	260 (19339), 350 (3308), 420 (1961)	-g	-g	620	-	2.00

a UV/vis absorptions measured in dichloromethane solutions.

b Emissions recorded at λ_exc_ = 450–470 nm in degassed acetonitrile solutions.

c Measured with a 450 nm laser.

d Emissions recorded at λ_exc_ = 450 nm in glassy 2-MeTHF solution.

e Excited-state energy estimated from the midpoint between the absorption and emission maxima at rt and converted into eV.

f Triplet-state energy estimated from the phosphorescence maximum and converted into eV.

g No luminescence was observed.

**Figure 3:**
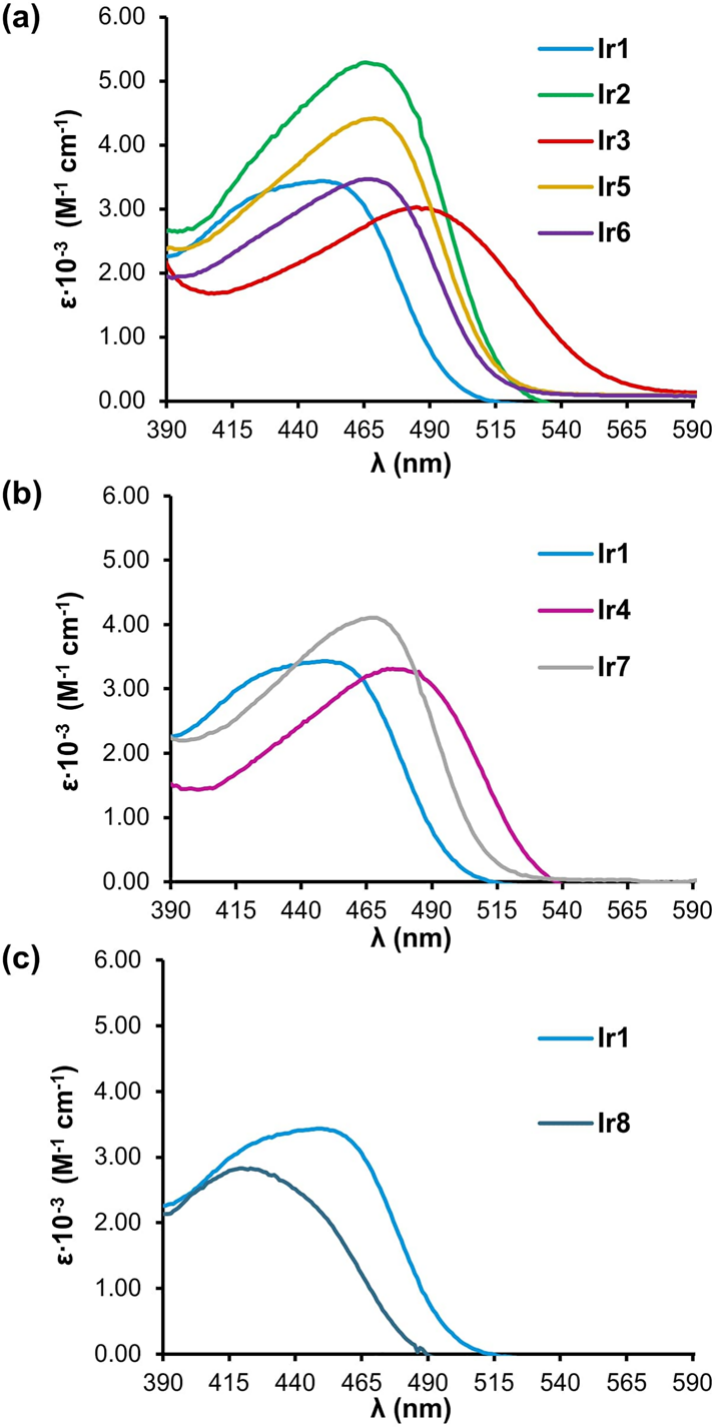
Comparative UV–vis spectra of **Ir1** and (a) 5-substituted 8-oxyquinolinate-Ir complexes, (b) 5,7-disubstituted oxyquinolinate-Ir complexes, and (c) 2-substituted 8-oxyquinolinate-Ir complex in dichloromethane.

The photophysical properties of the complexes were studied, having in mind that differently substituted 8-oxyquinolinate ligands are known to tune the absorption, emission, and redox properties of metallic complexes. First, the absorption spectra of the Ir complexes were recorded in dichloromethane solutions (see the SI for the full spectra and Table 2). All 8-oxyquinolinate-Ir(III) complexes showed an intense absorption band in the 250–300 nm region (ε > 12000 M^–1^·cm^–1^) ascribed to the π–π* transition of 8-oxyquinolinate and Cp* ligands. Another common feature is the presence of a broad absorption band within the 390–590 nm range. Such a band strongly depends on the 8-oxyquinolinate ligand nature, indicating a high ligand character contribution in such a transition, likely a metal-to-ligand charge transfer (MLCT) band. This is consistent with similar bands observed in other 8-oxyquinolinate-Ru(II) and 8-oxyquinolinate-Ir(III) complexes.^28,29^ Thus, the nonsubstituted 8-oxyquinolinate complex (**Ir1**) presents an absorption band at 430 nm that undergoes a significant bathochromic shift (35–55 nm) for complexes having 8-oxyquinolinates substituted either at the 5 or 5,7 positions, regardless of the electronic nature of the substituent (Table 2 and Figure 3a,b). On the contrary, the methyl substitution at the 2 position on the quinoline ring produces a 10 nm blueshift on the lowest energy maximum absorption band, which furthermore is less intense (ε = 1961 M^–1^·cm^–1^) than that of the other complexes (Table 2 and Figure 3c).

**Figure 4:**
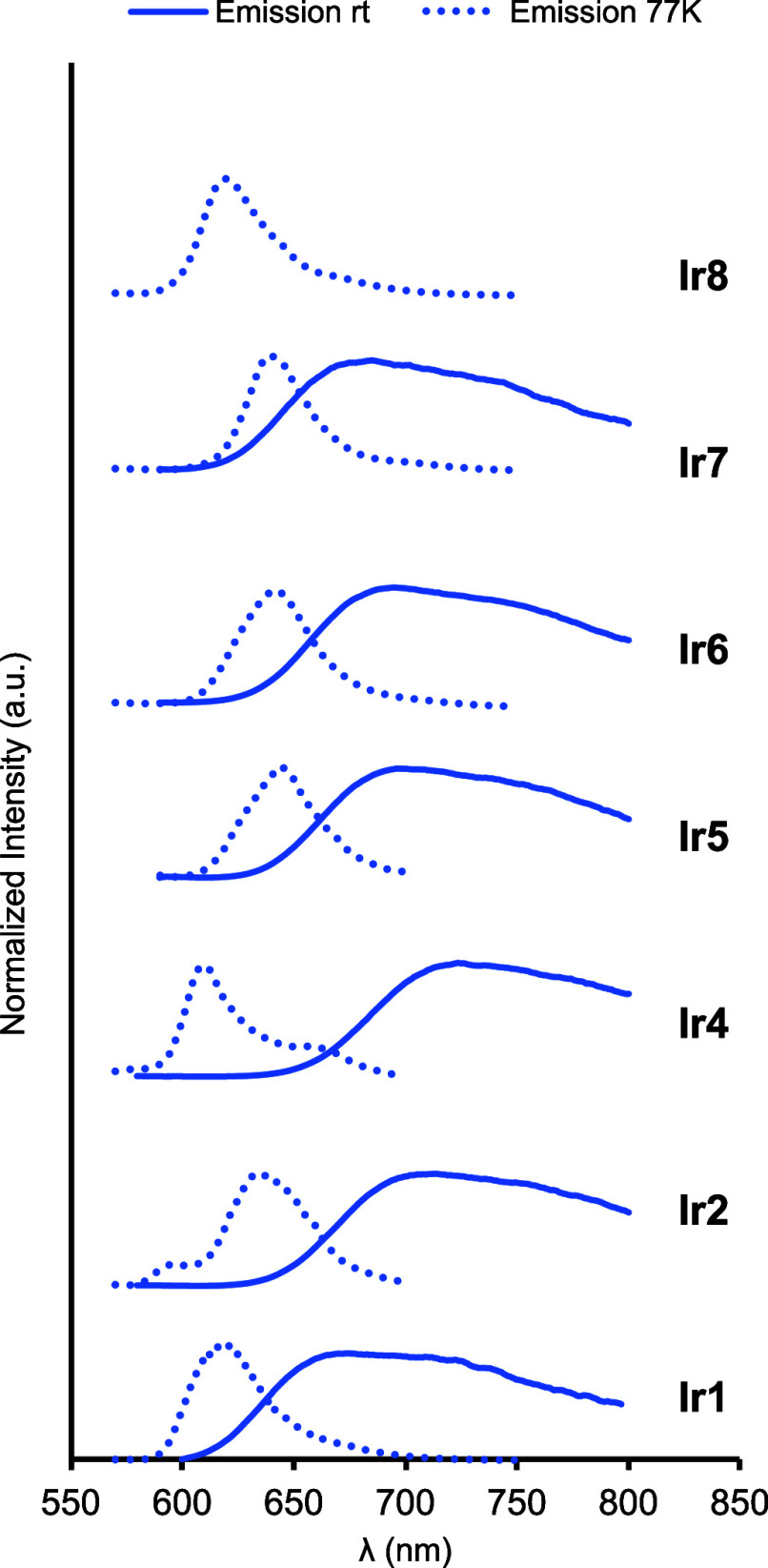
Overlaid room-temperature photoluminescence spectra of **Ir1**–**Ir8** complexes at 298 (solid line) and 77 K (dotted line). All the measurements were recorded in degassed acetonitrile for room-temperature emission and degassed glassy 2-MeTHF for low-temperature emission. Complexes **Ir3** and **Ir8** do not emit at room temperature.

Next, photoluminescence spectra were recorded by exciting at the highest wavelength band (λ_exc_ = 450–470 nm). All complexes, except for **Ir3** and **Ir8**, were emissive at room temperature in acetonitrile solutions (Figure 4 and Table 2). However, the presence of oxygen caused significant emission quenching. In addition, the concentration of the sample did not affect the intensity, shape, or maximum wavelength of the emission band. At room temperature, all complexes exhibited broad and unstructured emission bands in the red region (675–736 nm). Noteworthy, a large Stokes shift was observed for all the complexes (Δλ = 218–245 nm), which consequently prevents the overlap of absorption and emission spectra. Figure 4 depicts the stacked emission spectra of the Ir complexes at room temperature and 77 K, measured in glassy 2-methyltetrahydrofuran (2-MeTHF). The hypsochromic shift in the emission maxima for the low-temperature spectra in all cases can be easily noticed. This effect, named as luminescence rigidochromism, is associated with the change in the rigidity of the medium and its effect in dipole–dipole forces and has been previously observed in other organometallic and Ir complexes.^30−32^ Moreover, it is also reported that large shifts, as observed in **Ir1**–**Ir8** complexes, are indicative of a high contribution of the triplet MLCT state in the excited state.^33^ Based on the maximum emission wavelength at 77 K, the energy of the triplet excited states (*E*_T1_) was estimated for all iridium complexes except **Ir3**, which was not emissive, even at low temperatures (Table 2). It is important to note that the energy of the triplet excited states of these complexes is close to the 2.1 eV of the triplet excited-state energy of [Ir(ppy)_2_(bpy)]^+^^34^ and [Ru(bpy)_3_]^2+^.^35^ Finally, the photoluminescence lifetime (τ) ranges from 131.9 to 587.2 ns (Table 2), being the lowest value for the unsubstituted 8-oxyquinolate complex (**Ir1**), whereas the highest value corresponds to the 5-methyl-substituted 8-oxyquinolinate one (**Ir2**).

### Electrochemistry Studies

The electrochemical behavior of complexes **Ir1**–**Ir8** was evaluated by cyclic voltammetry experiments in acetonitrile. The complexes showed several redox processes. The first cathodic and anodic peak potentials for each complex are collected in Table 3. To study the individual waves, the redox processes were isolated by recording the cyclic voltammograms in a narrower potential window. The first oxidation process was found to be irreversible at a scan rate of 100 mV/s. However, increasing the scan rate showed a quasi-reversible behavior, suggesting a reversible electrochemical process followed by an irreversible chemical step (E_r_C_i_).^36^ This behavior was better observed for complex **Ir4** (see the SI). Conversely, the first reduction event was found to be irreversible in all cases. The full voltammograms are included in the Supporting Information.

**Table 3:** First Ground- and Excited-State Redox Potentials vs Fc/Fc^+^ of Ir Complexes

entry	Ir complex	*E*_pa_ (V)^a^	*E*_pc_ (V)^a^	*E*_ox_* (V)^b^	*E*_red_* (V)^b^
1	**Ir1**	0.47, 0.74	–2.10, −2.29	–1.77	0.14
2	**Ir2**	0.34	–2.13, −2.35	–1.76	–0.03
3	**Ir3**	0.21, 0.33	–2.26		
4	**Ir4**	0.33, 0.53, 0.69, 0.92	–2.30	–1.72	–0.25
5	**Ir5**	0.52, 0.66	–1.98, −2.22	–1.58	0.13
6	**Ir6**	0.53, 0.68	–1.98, −2.25	–1.61	0.16
7	**Ir7**	0.61, 0.83	–1.91, −2.14, −2.37	–1.55	0.25
8	**Ir8**	0.44, 0.72	–2.31		

a Cyclic voltammetry experiments were recorded under the following conditions: 100 mV·s^–1^ scan rate; 1.0 mM solution of the corresponding complex in argon-saturated MeCN solution; 0.1 M solution of Bu_4_NPF_6_; a glassy carbon disk (3.0 mm diameter) as a working electrode; a platinum sheet as a counter electrode; Ag/AgCl as a reference electrode. Potentials are referenced vs Fc/Fc^+^.

b Excited-state redox potentials estimated using the equation E*_ox_ = *E*_ox_ – *E*_0–0_ or E*_red_ = *E*_red_ + *E*_0–0_.

**Figure 5:**
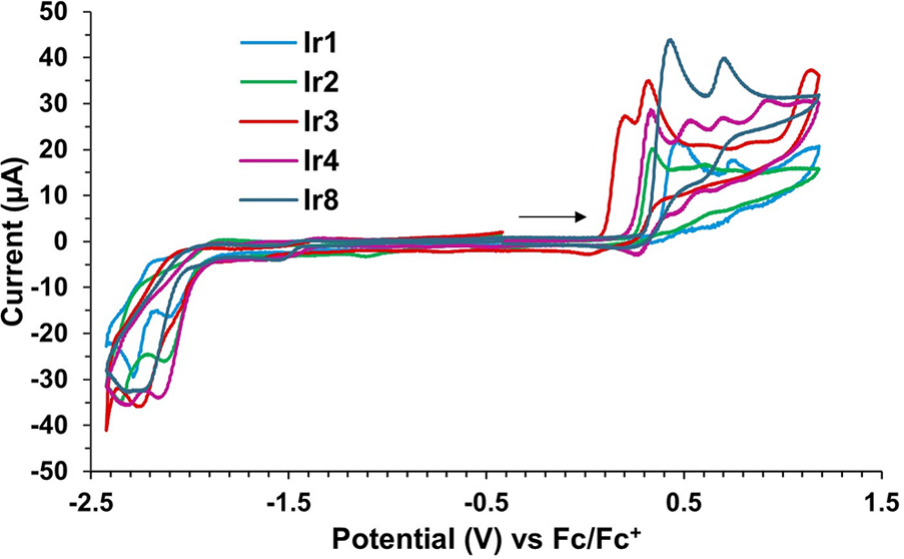
Cyclic voltammograms of complexes containing 8-oxyquinolinate ligands with electron-donating groups and parent **Ir1**. The arrow indicates the sweep direction.

**Figure 6:**
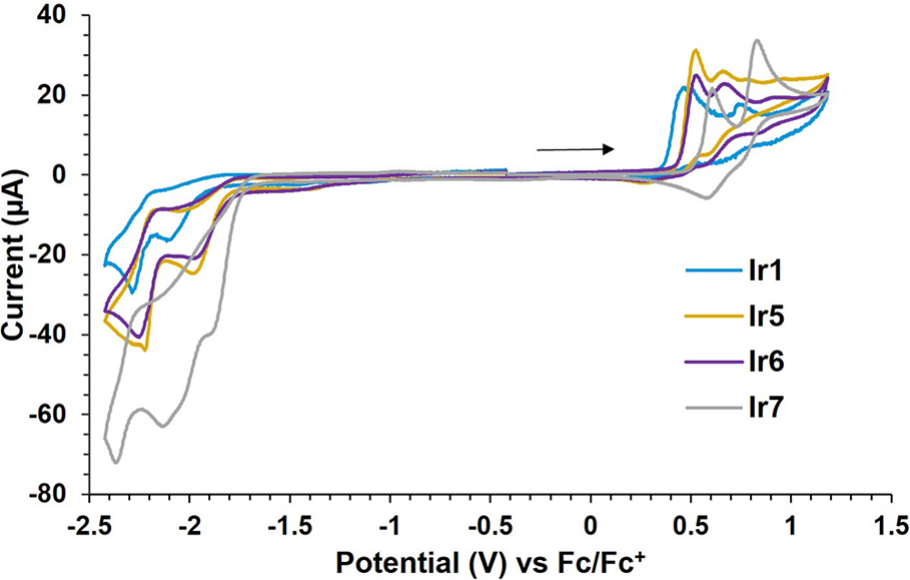
Cyclic voltammograms of complexes containing 8-oxyquinolinate ligands with electron-withdrawing groups and parent **Ir1**. The arrow indicates the sweep direction.

The redox potentials were affected by the nature of the 8-oxyquinolinate ligands. The first oxidation potential was found at 0.47 V for complex **Ir1**. As expected, this value suffered a cathodic shift for complexes containing 8-oxyquinolinates with electron-donating substituents (**Ir2**–**Ir4** and **Ir8**; *E*_pa_ = 0.21–0.44 V, Figure 5) and an anodic shift for those containing electron-withdrawing groups (**Ir5**–**Ir7**; *E*_pa_ = 0.52–0.61 V, Figure 6). In turn, the first reduction event was also modified upon the 8-oxyquinolinate substitution. While complex **Ir1** showed the first reduction at −2.10 V, complexes containing electron-donating groups were more difficult to reduce (*E*_pc_ = (−2.13) – (−2.31) V, Figure 5), and those bearing electron-withdrawing groups reduce at more positive potentials (*E*_pc_ = (−1.91) – (−1.98) V, Figure 6).

To estimate the excited-state redox potentials of the complexes, their ground-state redox values and their excited-state energies (*E*_0–0_) were considered. Regarding the ground-state redox potentials, the first oxidation or reduction value of the complexes was considered based on the fact that photoredox reactions are one-electron processes (Table 3). On the other hand, the excited-state energy (*E*_0–0_) is usually estimated from the intersection between the lowest energetic UV–vis band and the emission spectra. However, the considerable Stokes shift observed for all of the complexes precludes a crossover point. For this reason, the *E*_0–0_ values were calculated as the midpoint between the absorption and emission maxima and converted into eV (Table 2). Unfortunately, these values were not determined for the nonemissive complexes **Ir3** and **Ir8**. Having the ground-state oxidation and reduction potentials and the excited-state energy, the excited-state redox potentials of Ir complexes were estimated using the equation: E*_ox_ = *E*_ox_ – *E*_0–0_ or E*_red_ = *E*_red_ + *E*_0–0_, and the values are collected in Table 3. These excited-state redox values suggest that the complexes should act as strong reductants, being **Ir1** and **Ir2** the strongest ones (*E*_ox_* ∼ −1.76 V).

### Catalytic Studies

With the complexes in hand, their catalytic activities were next explored. Due to the photophysical properties above commented, **Ir1**–**Ir8** complexes were evaluated as photocatalysts in transformations involving different mechanistic pathways such as energy transfer and photoredox processes. Additionally, their ability to promote a transfer hydrogenation reaction was explored as well.

**Scheme 2:**
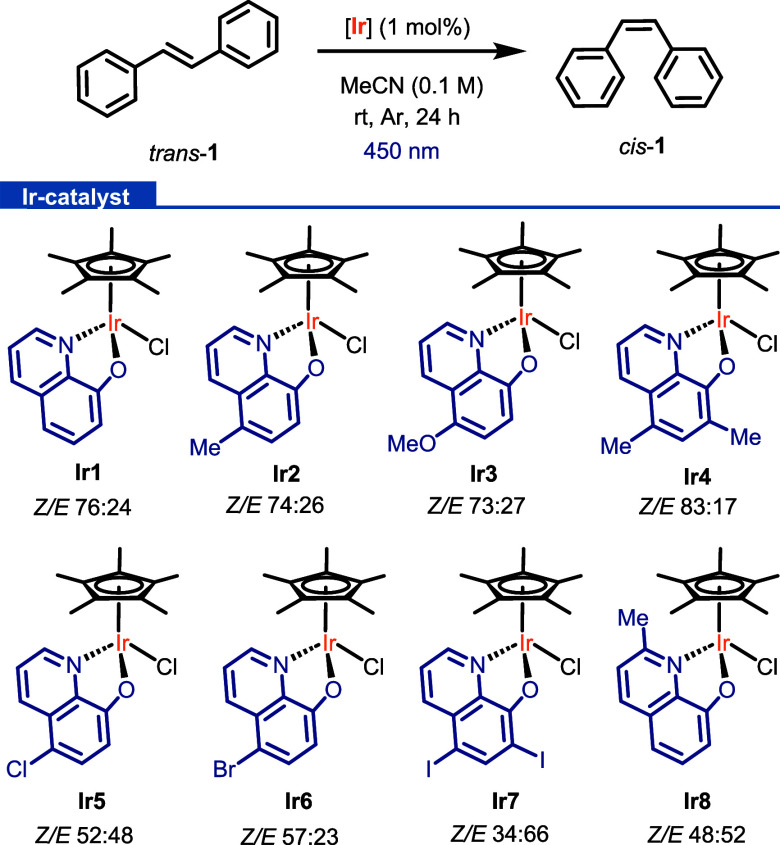
Catalyst Screening for the Photoisomerization of *trans*-Stilbene

The visible-light (*E*)- to (*Z*)-isomerization of double bonds using photocatalysts is a well-known process^34,37,38,39^ and can be used as a model reaction for evaluating the performance of a novel photocatalyst for energy transfer processes via triplet-state species. In this transformation, the triplet-state energies of both the alkene and the excited photocatalyst are used to estimate the thermodynamic feasibility of the reaction. Thus, following a Dexter energy transfer mechanism, the triplet energy of the excited state of the photocatalyst (donor) has to be higher than that of the alkene (acceptor) to effectively transfer the energy from the photocatalyst to the alkene, finally leading to its isomerization.^37,40^ Considering the estimated triple energy of **Ir1**–**Ir8** complexes (∼2.0 eV, Table 2), we decided to test them in the photoisomerization of *trans*-stilbene (**1**) using a 450 nm LED irradiation source (Scheme 2). The first data extracted from the catalyst screening show a clear ligand effect on the transformation. Complexes **Ir1**–**Ir4** afforded the highest *Z*/*E* isomerization ratio of **1**. Moreover, 83% of *cis*-**1** was achieved using **Ir4**, which is comparable with the 81% isomerization obtained using the well-known [Ru(bpy)_3_]^2+^.^35^ The best catalytic performance obtained for **Ir4** could be explained by considering its triplet excited-state energy (2.03 eV), which is the highest one along the series (Table 2). Consequently, it is capable of sensitizing *trans*-**1** (2.1 eV), considering the estimation error of the triplet-state energies of **Ir1**–**Ir8** complexes. Further optimization of the reaction conditions, in terms of the catalyst loading, concentration, and solvent (see SI, Table S1), did not improve the 83% isomerization reached using **Ir4** under the conditions indicated in Scheme 2. Lastly, control experiments carried out in the absence of light or a catalyst resulted in low conversions (<5%), showing the key role of both components for the isomerization.

**Scheme 3:**
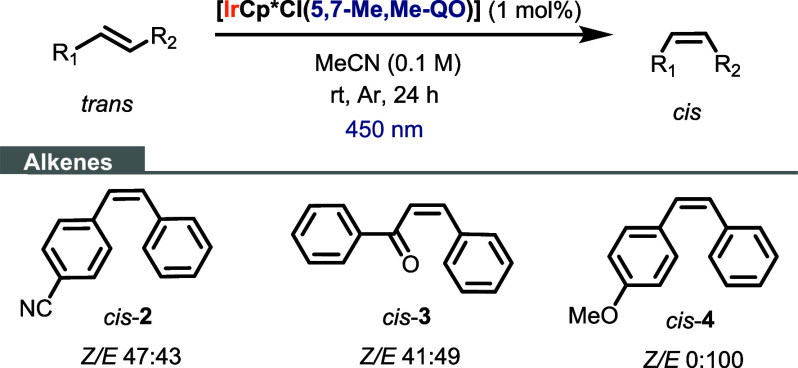
Photoisomerization of Different Alkenes Using **Ir4** as a Catalyst

Then, we evaluated the catalytic activity of **Ir4** [IrCp*Cl(5,7-Me,Me-QO)] for the isomerization of other alkenes (Scheme 3). *trans-p*-Cyanostilbene (*trans*-**2**) and *trans*-chalcone (*trans*-**3**) were moderately isomerized to their corresponding *cis*-alkenes, despite the slightly lower triplet energy (∼2.0 eV) of *trans*-**2** and *trans*-**3** alkenes compared to *trans*-stilbene. In agreement with the high triplet energy of *trans*-**4** (2.6 eV), **Ir4** was unable to catalyze its isomerization into *cis*-**4**.

**Scheme 4:**
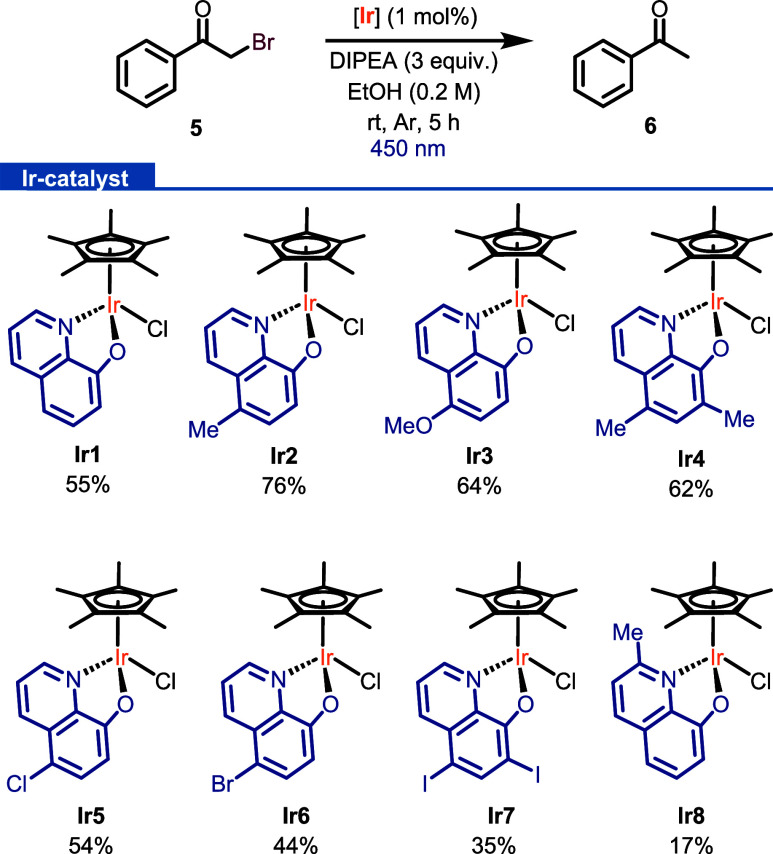
Catalyst Screening for the Debromination of **5** Yields were determined by GC analysis of the crude mixture.

Having demonstrated the potential of **Ir1**–**Ir8** complexes in energy transfer-mediated mechanisms, we next explored their catalytic performance in single-electron transfer processes. Among them, the formation of carbon-centered radicals from activated carbon–halogen bonds is an important step in both organic synthesis and decontamination of halogenated organic pollutants.^41,42^ Thus, to study the **Ir1**–**Ir8** complexes, we selected the photocatalytic dehalogenation reaction in which the radical intermediate reacts with a H-atom source to afford the corresponding dehalogenated derivative.^43,44^ First, the catalyst screening was evaluated in the debromination of 2-bromoacetophenone (**5**) using 1 mol % of **Ir1**–**Ir8** complexes,^45^ DIPEA as a hydrogen source, ethanol as a solvent, and 450 nm LED irradiation in the absence of air (Scheme 4). To identify differences in terms of catalytic performance, the reactions were stopped after 5 h. Under these reaction conditions, complexes having electron-donor substituents at the 8-oxyquinolinate ring showed remarkable catalytic performance. In particular, **Ir2** was the most active complex, being able to catalyze the debromination of **5** in 76% of yield. Here again, the low conversions obtained in the control experiments performed for this transformation proved the role of the Ir catalyst and light in the reactivity observed.

**Scheme 5:**
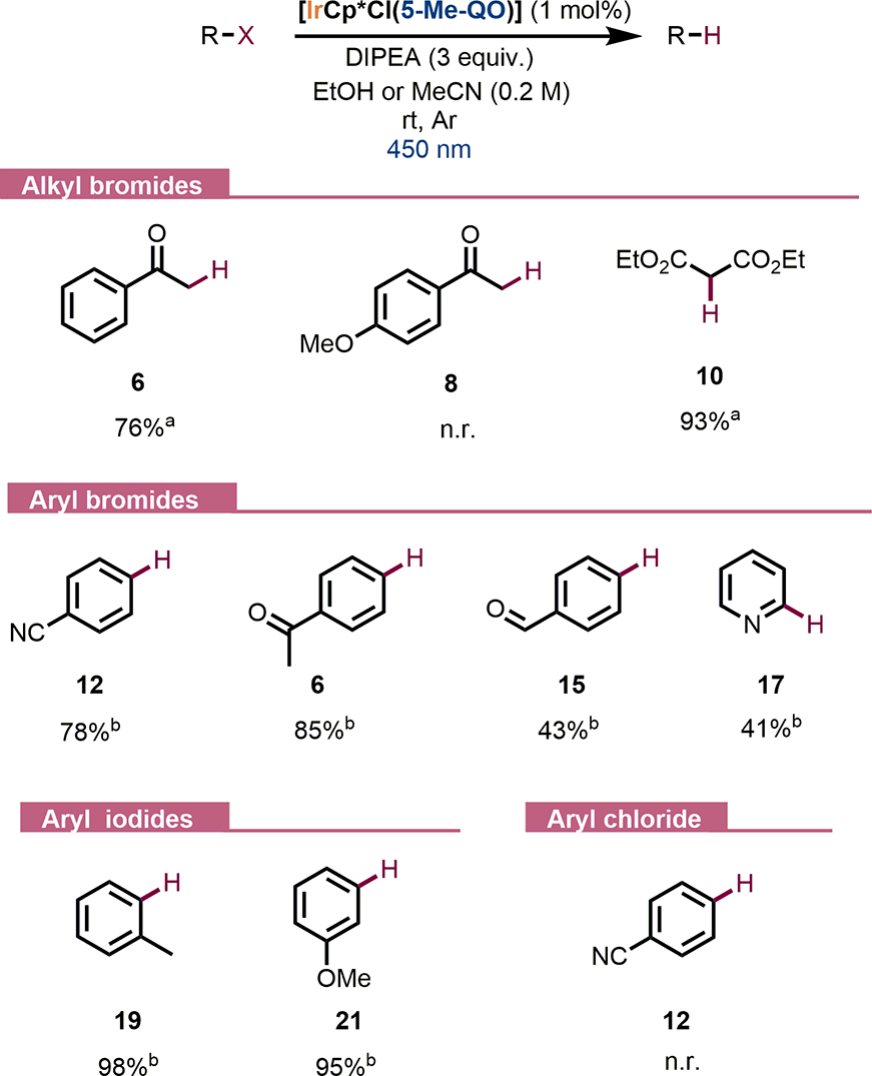
Dehalogenation Reaction Scope Promoted by the **Ir2** Complex (a) Reaction conditions: EtOH (0.2 M), 450 nm LED stripe, setup 2 (see the SI). (b) Reaction conditions: MeCN (0.2 M), single 450 nm LED, setup 1 (see the SI). ^1^H NMR yields with 1,3,5-trimethoxybenzene as an internal standard.

Next, the applicability of the **Ir2** complex was evaluated studying its catalytic activity toward the dehalogenation of different alkyl and aryl halides, including C–I, C–Br, and C–Cl bonds (Scheme 5). The dehalogenation of activated alkyl bromides with 1 mol % of catalyst **Ir2** proceeded smoothly in 5 h of reaction time to afford compounds **6** and **10**, though the debromination of electron-rich acetophenones, with high reduction potentials, was unsuccessful (see the SI). On the other hand, more challenging aryl bromides were satisfactorily reduced in moderate to good yields (41–85%). The activity exerted by **Ir2** toward the formation of pyridine (**17**) is remarkable due to the high reduction potential of bromopyridine (**16**) (*E*_red_= −2.26 V vs SCE).^46^ Moreover, the synthesis of acetophenone (**6**) could be successfully accomplished via the debromination reaction of either 2-bromoacetophenone (**5**) or 4′-bromoacetophenone (**13**). Additionally, the reduction of electron-rich aryl iodides was easily achieved, affording **19** and **21** in 98 and 95% yields, respectively. As expected, **Ir2** was unable to reduce substrates containing C–Cl bonds (see Scheme 5).

**Scheme 6:**
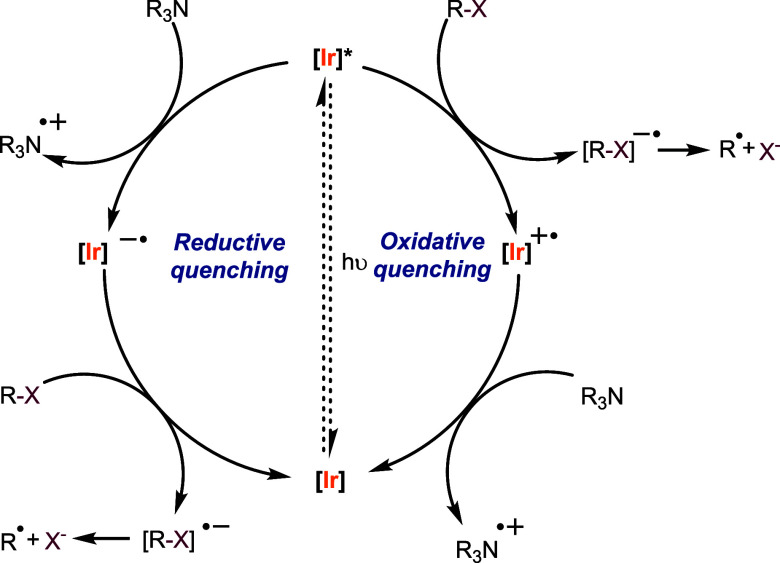
Photoredox Pathways for the Debromination Reaction

**Figure 7:**
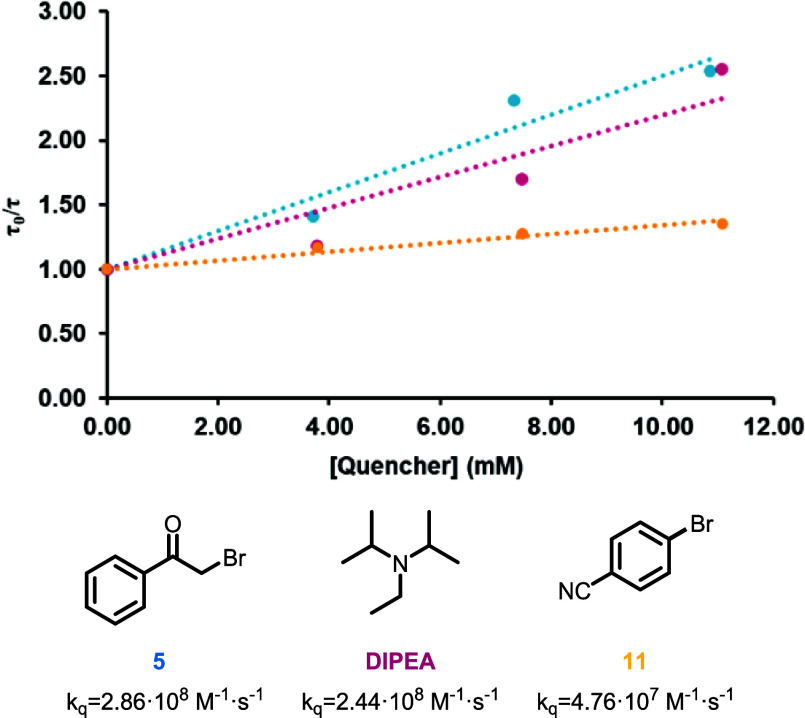
Top: Stern–Volmer plots of excited-state **Ir2** quenched by bromides **5** (blue line), **11** (yellow line), and DIPEA base (pink line). Bottom: Calculated quenching rate constants (*k*_q_). For all experiments, λ_ex_ = 450 nm.

As shown in Scheme 6, the debromination reaction can proceed via two different pathways, namely, reductive or oxidative quenching pathways, depending on the substrate that quenches the excited state of the catalyst.^41,42^ To discern between them, quenching experiments of **Ir2** were conducted using either DIPEA or an organic bromide (**5** or **11**) to gain insights into the kinetics of both plausible pathways. Initially, the steady-state emission at room temperature was recorded after the incremental addition of **5** or **11**. In all cases, the emission intensity decreased without altering the spectral shape, indicating the involvement of the excited state of **Ir2** in the photoinduced electron transfer process. Next, time-resolved emission experiments were also carried out, and the fluorescence lifetime of **Ir2** was monitored as a function of the added quencher concentration (**5**, **11**, or DIPEA) (Figure 7). The quenching rate constant, *k*_q_, for each quencher was determined through Stern–Volmer analysis (Figure 7). Thus, *k*_q_ was calculated using the formula *K*_sv_= *k*_q_·τ_0_, where *K*_sv_ is the Stern–Volmer constant obtained from the slope value of each plot and τ_0_ denotes the emission lifetime of the complex in the absence of a quencher (Figure 7 and the SI). The first information extracted from the *k*_q_ values is the different quenching efficiency of both bromides, which is six times higher for the alkyl bromide **5**. Moreover, upon comparison of the quenching rate constants of aryl bromide **11** and DIPEA, the excited state of **Ir2** is quenched faster by DIPEA, suggesting that a reductive quenching mechanism is taking place. This is the most common mechanism proposed for other metal-based photocatalysts.^47^ By contrast, the kinetics of the debromination of **5** showed a more complex scenario. The rate constants of **5** (*k*_q_ = 2.86 × 10^8^ M^–1^·s^–1^) and DIPEA (*k*_q_ = 2.44 × 10^8^ M^–1^·s^–1^) are very similar, indicating that any of the quenching pathways are possible as well as both quenchers are competing with the excited state of the catalyst.

**Scheme 7:**
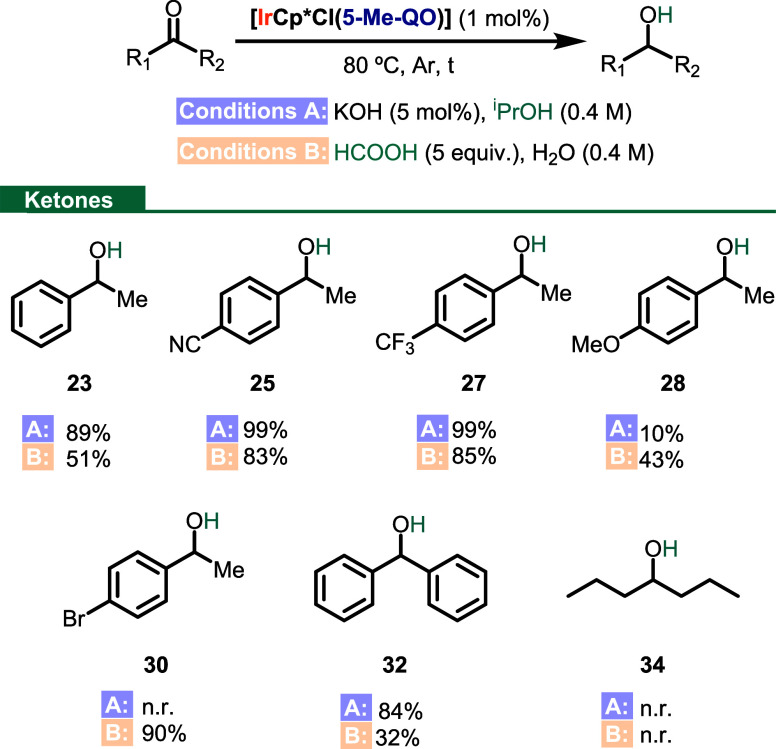
Transfer Hydrogenation Scope under Conditions A and B

Once the applicability of [IrCp*Cl(N^O)] complexes as photocatalysts was established, their ability to promote traditional transfer hydrogenation employing isopropyl alcohol and formic acid as hydrogen donors was evaluated. With the idea of combining photocatalysis and traditional organometallic catalysis employing a single catalyst in mind, complex **Ir2**, which was the best catalyst of the series in the debromination reaction, was selected to explore this transformation. As expected,^7^ complex **Ir2** was able to promote transfer hydrogenation from isopropanol to different ketones (Scheme 7, conditions A). The reaction worked well with acetophenone (**6**) and gave excellent yields with acetophenone derivatives bearing electron-withdrawing groups (99%, **25** and **27**, Scheme 7). However, the reaction with methoxy- and bromo-substituted acetophenones (**8** and **13**) afforded very low conversions (**28**) or no reaction at all (**30**), respectively. Finally, while benzophenone (**31**) was converted to the desired alcohol **32** in good yields (84%), 4-heptanone (**33**) did not afford the corresponding alcohol (**34**).

Transfer hydrogenation from formic acid was also successful (Scheme 7, conditions B).^48^ In general, slightly lower yields were obtained when compared to the reaction with 2-propanol (Scheme 7, **23**, **25**, **27**, and **32**). However, the reaction worked better with 4′-methoxyacetophenone (**8**) and 4′-bromoacetophenone (**13**), affording the corresponding alcohols, **28** and **30**, in 43 and 90% yield, respectively. The dialkylic ketone **33** did not undergo TH in the presence of formic acid either.

**Scheme 8:**
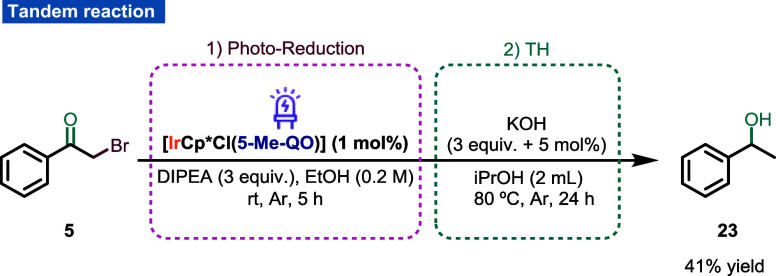
Proof-of-Concept Sequential Photodebromination/Transfer Hydrogenation Reactions Employing a Single Catalyst

To further show the versatility of the [IrCp*Cl(N^O)] complexes, the possibility of combining both catalytic reactivities (photocatalytic single-electron transfer and transfer hydrogenation) by employing a single catalyst was sought. Gratifyingly, 2-bromoacetophenone (**5**) was converted into 1-phenylethanol (**23**) in a sequential catalytic process involving first the photodebromination of **5** and the subsequent transfer hydrogenation of the resulting ketone with **Ir2** as the sole catalyst (Scheme 8). Even though the compatibility of both reaction conditions was difficult, alcohol **23** was obtained in 41% yield from **5** without the need of isolation or purification of any intermediate. It should be noted that no additional catalyst was added to perform the second step. To the best of our knowledge, this is the first example of a sequential catalysis mediated by a single Ir complex able to sequentially carry out a photoreductive debromination/transfer hydrogenation.

## Conclusions

In conclusion, we have shown the versatility of the [IrCp*Cl(N^O)] complexes. These complexes have been demonstrated to be active photocatalysts in energy transfer and single-electron transfer benchmark examples. The electronic nature of the 8-oxyquinolinate ligand has proven to have a deep impact on the properties and catalytic activity of the complexes. Additionally, these complexes present the expected traditional organometallic behavior and are able to promote transfer hydrogenation of ketones from isopropanol. This double catalytic activity has allowed us to develop a tandem process combining both reactivities using a single catalyst. This remarkable result proves the high versatility and applicability of the [IrCp*Cl(N^O)] complexes. Further studies to expand the reactivity of these complexes are currently ongoing in our laboratories.

## Experimental Section

The complete experimental section is included in the Supporting Information.

## Supporting Information Available

Synthesis and full characterization of all Ir compounds, catalytic procedures, absorption and emission spectra, electrochemical characterization and NMR spectra of all Ir compounds (PDF)

## Accession Codes

CCDC 2312176, 2312196, and 2312197 numbers contain the supplementary crystallographic data for complexes **Ir2**, **Ir4**, and **Ir8**. These data can be obtained free of charge via www.ccdc.cam.ac.uk/data_request/cif, or by emailing data_request@ccdc.cam.ac.uk, or by contacting The Cambridge Crystallographic Data Centre, 12 Union Road, Cambridge CB2 1EZ, UK; fax: + 44 1223 336033.
