# Supplementary material for: Double Catalytic Activity Unveiled: Synthesis, Characterization, and Catalytic Applications of Iridium Complexes in Transfer Hydrogenation and Photomediated Transformations
Source: ACS Catal. 2024 Apr 11;14(9):6413–22. doi: 10.1021/acscatal.4c00673 (PMC11075020; doi:10.1021/acscatal.4c00673)
Supplement: Supplementary file 1 — cs4c00673_si_001.pdf [file cs4c00673_si_001.pdf]

# SUPPORTING INFORMATION

# Doble Catalytic Activity Unveiled: Synthesis, Characterization, and Catalytic Applications of Iridium Complexes in Transfer Hydrogenation and Photo-Mediated Transformations

Laura Blanco,[a] Andrea Uroz,[b] Kevin Gutiérrez,[a] Silvia Cabrera,[a,c,*] Alba Collado,[a,c,*] José Alemán[b,c,*]

[a] *Inorganic Chemistry Department, Module 7, Universidad Autónoma de Madrid, 28049-Madrid (Spain).*

[b]*Organic Chemistry Department, Module 2, Universidad Autónoma de Madrid, 28049-Madrid (Spain).*

[c]*Institute for Advanced Research in Chemical Sciences (IAdChem), Universidad Autónoma de Madrid, Madrid (Spain).*

*E-mail: silvia.cabrera@uam.es; alba.collado@uam.es; jose.aleman@uam.es

**1. General information** .......... 3
**2. Materials and Methods** .......... 4
**3. General Procedure A for synthesis of [IrCp*Cl(N^O)] complexes** .......... 5
**4. Procedures for photocatalytic reactions (B and C)** .......... 8
**4.1. General procedure B for isomerization reactions** .......... 8
**4.2 General procedure C for dehalogenation reactions** .......... 10
**4.2.1. General procedure C1: Dehalogenation reactions of $Csp^3$ halides** .......... 10
**4.2.2. General procedure C1: Dehalogenation reactions of $Csp^2$ halides** .......... 11
**4.2.3. Stability test for Ir6 and Ir7 under photocatalytic conditions** .......... 12
**5. General procedure for transfer hydrogenation reactions** .......... 14
**5.1. General procedure D for transfer hydrogenation reactions using 2-propanol** .......... 14
**5.2. General procedure E for transfer hydrogenation reactions using formic acid** .......... 14
**6. General procedure E for one-pot sequential reaction** .......... 16
**7. Optimization of isomerization reactions** .......... 17
**8. UV-Vis absorption data for complexes [IrCp*Cl(N^O)]** .......... 17
**9. Emission spectra data for complexes [IrCp*Cl(N^O)]** .......... 22
**10. Stern-Volmer quenching studies for [IrCp*Cl(N^O)]** .......... 24
**11. Electrochemistry** .......... 25
**11.1 Cyclic voltammograms (CV) of Ir1-Ir8** .......... 26
**11.2 Scan rate dependence of first oxidation process of Ir1-Ir8** .......... 30
**12. NMR spectra** .......... 34
**13. Single Crystal X-Ray Diffraction of Ir2, Ir4 and Ir8** .......... 39
**14. References** .......... 42

## 1. General information

NMR spectra were acquired on a Bruker Avance 300 MHz and Bruker Avance Neo 500 spectrometers, running at 300 and 500 MHz for $^{1}$H and 75 MHz and 126 MHz for $^{13}$C, respectively. Chemical shifts (δ) are reported in ppm relative to residual solvent signals ($CDCl_3$: 7.26 ppm for $^{1}$H NMR, 77.0 ppm for $^{13}C\{^{1}H\}$ NMR; $CD_2Cl_2$: 5.32 ppm for $^{1}$H NMR, 53.8 ppm for $^{13}C\{^{1}H\}$ NMR). $^{13}C\{^{1}H\}$ NMR was acquired on a broad band decoupled mode. Coupling constants are given in Hz. The following abbreviations are used to describe peak patterns when appropriate: s (singlet), d (doublet) dd (doublet of doublets), t (triplet), q (quartet) and m (multiplet).

Electrospray ionization in positive mode (ESI+) has been used for measuring the exact mass of each compound (indicated for each case) using a MAXIS II Bruker spectrometer.

For elemental analysis a LECO CHNS-932 elementary analyzer was employed.

UV-Vis measurements were acquired on an Agilent 8453 UV-Vis Spectrophotometer controlled by UV-Visible ChemStation Software. HPLC grade $CH_2Cl_2$ solvent and a Teflon-top 10x10 mm precision cell made of quartz SUPRASIL® were used for all measurements. Emission intensities and time-resolved emission spectra were recorded using an Edinburg Instruments FS5 Spectrofluorometer in an oxygen-free atmosphere. Samples were excited at 420-500 nm using a Xenon lamp and at 450 nm using an EPL-450 diode laser. Samples for room-temperature emission were prepared in degassed HPLC grade MeCN and were kept in 1 cm quartz cuvettes with septum-sealed screw caps. Samples for low-temperature emission were done in a glassy 2-MeTHF media, contained in a quartz EPR tube, and immersed in liquid nitrogen. The IRF (Instrument Response Function) was acquired at the fluorescence wavelength using LUDOX. The decay trace was fitted using the instrument's analysis software.

Crystal of complexes [IrCp*Cl(5-Me-QO)] **Ir2**, [IrCp*Cl(5,7-Me,Me-QO)] **Ir4** and [IrCp*Cl(2-Me-QO)] **Ir8** were obtained by the controlled diffusion of n-pentane in a saturated solution of the corresponding complex in dichloromethane. In the case of **Ir2** and **Ir4,** the crystals were mounted at low temperature in inert oil on a glass fibre. Data were collected on Bruker Kappa Apex II diffractometer equipped with a graphite monochromated MoKα radiation source (λ = 0.71073 Å). Data were integrated using the Bruker SAINT software package[1] using a narrow-frame algorithm. Data was corrected for absorption effects using the Multi-Scan method (SADABS).[2] The structures were solved and refined using the Bruker SHELXTL software package and refined by full-matrix least-squares methods based on F2. All non-hydrogen atoms were refined with anisotropic thermal parameters. All H atoms were computed and refined with an overall isotropic temperature factor using a riding model. For complex **Ir8**, a suitable crystal was selected and placed on a MiTeGen micromount on a XtaLAB Synergy R, HyPix-Arc 100 diffractometer equipped with a graphite monochromated CuKα radiation source (λ = 1.5418 Å). The crystal was kept at 250.00(10) K during data collection. Using Olex2, the structure was solved with the SHELXT structure solution program using Intrinsic Phasing and refined with the SHELXL refinement package using Least Squares minimisation.[3]

## 2. Materials and Methods

Commercial grade reagents and solvents were purchased from Acros Organics, Alfa Aesar, Fluorochem, Sigma-Aldrich, BLD Pharm, and TCI Chemicals, and used as received without further purification. Dichloromethane and acetonitrile were dried using 4 and 3 Å molecular sieves, respectively.

Two different custom-made photoreactor setups were used for the photocatalytic reactions:

**Setup 1:** In the case of isomerization reactions and dehalogenation reaction of $Csp^2$ substrates, the vial is placed inside the fitted well in which irradiation takes place at the desired wavelengths (450 nm was employed during this project) using 380 mW single LEDs. Reaction temperature is easily controlled (20ºC) using a recirculating system.

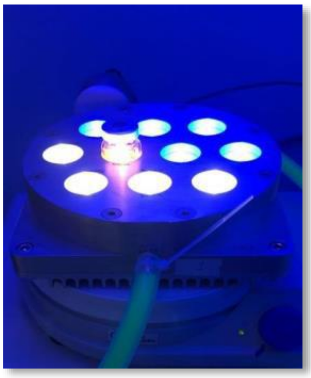

**Setup 2:** A custom-made "light box" with 5 strips of blue LEDs (15 W) attached around a test tube rack was used in the case of dehalogenation reactions of $Csp^3$ substrates, with a fan for keeping the reaction mixture at room temperature.

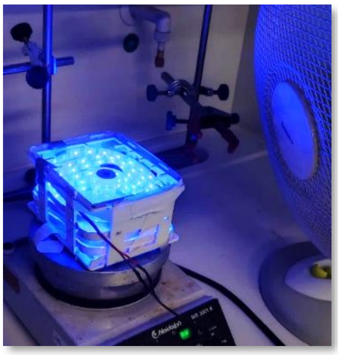

## 3. General Procedure A for synthesis of [IrCp*Cl(N^O)] complexes

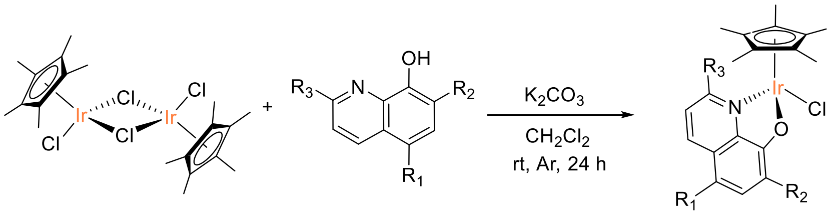


**Ir1 - Ir8**

All synthesis were carried out under argon using standard Schlenk techniques. $[Cp^*IrCl_2]_2$ (50 mg, 0.0627 mmol) was dissolved in 4 mL of degassed dichloromethane in a Schlenk flask. Then, the corresponding commercial 8-hydroxyquinoline (0.188 mmol) and potassium carbonate (78.0 mg, 0.565 mmol) were added. The reaction mixture was stirred at room temperature for 24 h, filtered through a mix of Celite and $SiO_2$ pad and elute with dichloromethane (3 x 10 mL). To completely elute **Ir2** and **Ir4** complexes ethyl acetate (3 x 5 mL) was also used. The resulting solution was concentrated under reduced pressure. Then, the final product was obtained by precipitation and was washed (3 x 5 mL) with n-pentane.

**[IrCp*Cl(QO)] (Ir1)**

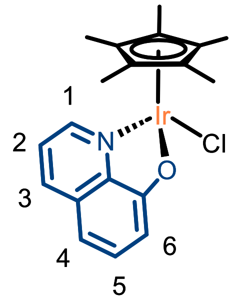


Yellow solid (58 mg, 91%) **$^{1}$H NMR** (300 MHz, $CDCl_3$): δ (ppm) 8.55 (d, *J* = 4.9 Hz, 1H, $CH_{(H-1)}$), 8.04 (d, *J* = 8.3 Hz, 1H, $CH_{(H-3)}$), 7.37 (t, *J* = 8.0, 1H, $CH_{(H-5)}$), 7.31 (dd, *J* = 8.3, 4.9, 1H, $CH_{(H-2)}$), 7.00 (d, *J* = 8.0 Hz, 1H, $CH_{(H-4)}$), 6.79 (d, *J* = 8.0 Hz, 1H, $CH_{(H-6)}$), 1.73 (s, 15H, $CH_{3(Cp*)}$). Spectroscopic data matched reported data for this compound.[4]

**[IrCp*Cl(5-Me-QO)] (Ir2)**

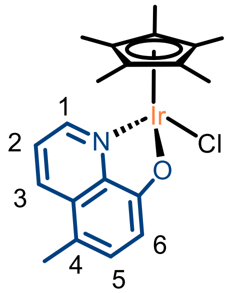


Yellow solid (62 mg, 95%). **$^{1}$H NMR** (500 MHz, $CD_2Cl_2$) δ (ppm) 8.58 (dd, *J* = 4.9, 1.2 Hz, 1H, $CH_{(H-1)}$), 8.19 (dd, *J* = 8.6, 1.2 Hz, 1H, $CH_{(H-3)}$), 7.40 (dd, *J* = 8.6, 4.9 Hz, 1H, $CH_{(H-2)}$),), 7.21 (d, *J* = 7.9 Hz, 1H, $CH_{(H-6)}$), 6.75 (d, *J* = 7.9 Hz, 1H, $CH_{(H-5)}$), 2.53 (s, 3H, $CH_{3(C4)}$), 1.72 (s, 15H, $CH_{3(Cp*)}$).). **$^{13}$C{$^{1}$H} NMR** (126 MHz, $CD_2Cl_2$) δ (ppm): 168.0 (C), 146.8 (C), 146.2 ($CH_{(C1)}$), 135.1 ($CH_{(C3)}$), 131.1 ($CH_{(C6)}$), 130.0 (C), 122.1 ($CH_{(C2)}$), 117.6 (C), 114.2 ($CH_{(C5)}$), 85.1 (C($_{Cp*}$)), 17.0 ($CH_{3(C4)}$), 9.0 ($CH_{3(Cp*)}$). **ATR-FTIR** ($cm^{-1}$): 3032, 2967, 2921, 1560, 1537, 1477, 1434. **MS** (ESI+): m/z calcd. for $C_{20}H_{23}IrNO$ [$M^{+}$-Cl] 486.1409, found: 486.1394. **Anal**. Calcd. for $C_{20}H_{23}IrClNO$: C, 46.10; H, 4.45, N, 2.69; found: C, 46.11; H 4.52; N, 2.66.

**[IrCp*Cl(5-OMe-QO)] (Ir3)**,

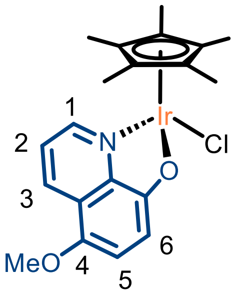


Red solid (58 mg, 86%). **$^{1}$H NMR** (300 MHz, $CD_2Cl_2$) δ (ppm) δ 8.57 (dd, *J* = 5.0, 1.4 Hz, 1H, $CH_{(H-1)}$), 8.40 (dd, *J* = 8.5, 1.4 Hz, 1H, $CH_{(H-3)}$), 7.35 (dd, *J* = 8.5, 5.0 Hz, 1H, $CH_{(H-2)}$), 6.90 (d, *J* = 8.6 Hz, 1H, $CH_{(H-6)}$), 6.73 (d, *J* = 8.6 Hz, 1H, $CH_{(H-5)}$), 3.90 (s, 3H, $CH_{3(OMe)}$) and 1.69 (s, 15H, $CH_{3(Cp*)}$). Spectroscopic data matched reported data for this compound.[5]

**[IrCp*Cl(5,7-Me, Me-QO)]  (Ir4)**

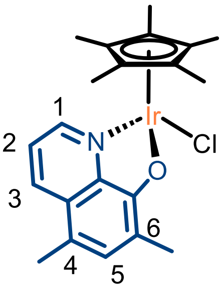


Orange solid (62 mg, 92%). **$^1$H NMR (**500 MHz, $CD_2Cl_2$) δ (ppm) 8.54 (dd, *J* = 4.9, 1.3 Hz, 1H, $CH_{(H-1)}$), 8.14 (dd, *J* = 8.5, 1.3 Hz, 1H, $CH_{(H-3)}$), 7.32 (dd, *J* = 8.5, 4.9 Hz, 1H, $CH_{(H-2)}$), 7.16 (s, 1H, $CH_{(H-5)}$), 2.52 (s, 3H, $CH_{3(C4)}$), 2.44 (s, 3H, $CH_{3(C6)}$) and 1.68 (s, 15H, $CH_{3(Cp*)}$). **$^{13}$C{$^1$H} NMR** (126 MHz, $CD_2Cl_2$) δ (ppm): 165.2 (C), 146.4 ($CH_{(C1)}$), 145.4 (C), 135.0 ($CH_{(C3)}$), 133.2 ($CH_{(C5)}$), 128.2 (C), 123.9 (C), 120.9 ($CH_{(C2)}$), 117.1 (C), 85.0 ($C_{(Cp*)}$), 16.8 ($CH_{3(C4)}$), 15.8 ($CH3_{(C6)}$), 8.9 ($CH_{3(Cp*)}$). **ATR-FTIR** ($cm^{-1}$): 2918, 1566, 1507, 1467, 1444. **MS** (ESI+): m/z calcd. for $C_{21}H_{25}IrNO$ [$M^+$-Cl] 500.1565, found: 500.1552. **Anal**. Calcd. for $C_{21}H_{25}IrClNO$: C, 47.14; H, 4.71, N, 2.62; found: C, 47.27; H, 4.87, N, 2.84.

**[IrCp*Cl(5-Cl-QO)] (Ir5)**

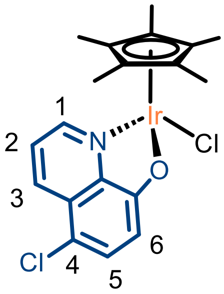


Dark orange solid (55 mg, 81%) **$^1$H NMR (**300 MHz, $CD_2Cl_2$) δ (ppm) 8.59 (dd, *J* = 4.9, 1.3 Hz, 1H, $CH_{(H-1)}$), 8.37 (dd, *J* = 8.6, 1.3 Hz, 1H, $CH_{(H-3)}$), 7.49 (dd, *J* = 8.6, 4.9 Hz, 1H, $CH_{(H-2)}$), 7.45 (d, *J* = 8.6 Hz, 1H, $CH_{(H-5)}$), 6.78 (d, *J* = 8.6 Hz, 1H, $CH_{(H-6)}$) and 1.69 (s, 15H, $CH_{3(Cp*)}$). Spectroscopic data matched reported data for this compound.[6]

**[IrCp*Cl(5-Br-QO)] (Ir6)**

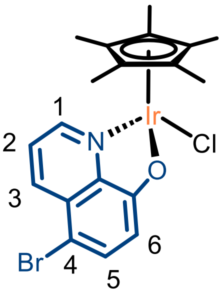


Yellow solid (65 mg, 88%) **$^1$H NMR (**300 MHz, $CDCl_3$) δ (ppm) 8.57 (dd, *J* = 4.9, 1.1 Hz, 1H, $CH_{(H-1)}$), 8.32 (dd, *J* = 8.6, 1.1 Hz, 1H, $CH_{(H-3)}$), 7.60 (d, *J* = 8.6 Hz, 1H, $CH_{(H-5)}$), 7.43 (dd, *J* = 8.6, 4.9 Hz, 1H, $CH_{(H-2)}$), 6.89 (d, *J* = 8.6 Hz, 1H, $CH_{(H-6)}$) and 1.73 (s, 15H, $CH_{3(Cp*)}$). Spectroscopic data matched reported data for this compound.[7]

**[IrCp*Cl(5,7-I, I-QO)] (Ir7)**

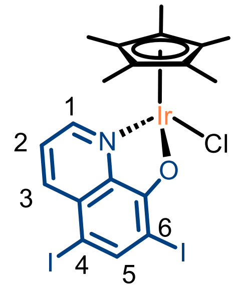


Orange solid (90 mg, 95%) **$^1$H NMR (**300 MHz, $CDCl_3$) δ (ppm) 8.53 (d, $J$ = 4.8 Hz, 1H, $CH_{(H\text{-}1)}$), 8.22 (s, 1H, $CH_{(H\text{-}5)}$), 8.16 (d, $J$ = 8.5 Hz, 1H, $CH_{(H\text{-}3)}$), 7.44 (dd, $J$ = 8.5, 4.8 Hz, 1H, $CH_{(H\text{-}2)}$) and 1.72 (s, 15H, $CH_{3(Cp*)}$). Spectroscopic data matched reported data for this compound.[7]

**[IrCp*Cl(2-Me-QO)] (Ir8)**

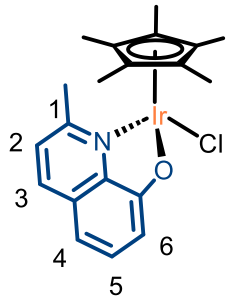


Yellow solid (65 mg, 86%) **$^1$H NMR (**500 MHz, $CD_2Cl_2$) δ 7.91 (d, $J$ = 8.5 Hz, 1H, $CH_{(H\text{-}3)}$), 7.32 (d, $J$ = 8.5 Hz, 1H, $CH_{(H\text{-}2)}$), 7.26 (t, $J$ = 7.9 Hz, 1H, $CH_{(H\text{-}5)}$), 6.81 (d, $J$ = 7.9, 1H, $CH_{(H\text{-}6)}$), 6.71 (d, $J$ = 7.9, 1H, $CH_{(H\text{-}4)}$), 3.08 (s, 3H, $CH_{3(C1)}$), 1.62 (s, 15H, $CH_{3(Cp*)}$).**$^{13}$C{$^1$H} RMN** (126 MHz, $CD_2Cl_2$) δ (ppm): 168.9 (C), 157.2 (C), 145.4 (C), 137.8 ($CH_{(C3)}$), 129.0 (C), 128.8 ($CH_{(C5)}$), 122.8 ($CH_{(C2)}$), 115.3 ($CH_{(C6)}$), 110.7 ($CH_{(C4)}$), 84.8 ($C_{(Cp*)}$), 28.4 ($CH_{3(C1)}$), 8.9 ($CH_{3(Cp*)}$). **ATR-FTIR** ($cm^{-1}$): 3039, 2958, 2922, 1563, 1506. **MS** (ESI+): m/z calcd. for $C_{20}H_{23}IrNO$ [$M^+$-Cl] 486.1409, found: 486.1393. **Anal**. Calcd. for $C_{20}H_{23}IrClNO$: C, 46.10; H, 4.45, N, 2.69; found: C, 45.86; H, 4.56; N, 2.58.

## 4. Procedures for photocatalytic reactions (B and C)

### 4.1. General procedure B for isomerization reactions

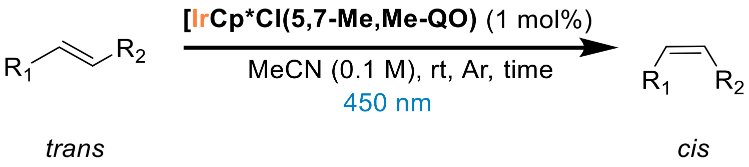


A vial equipped with a magnetic stir bar was charged with the corresponding *trans*-alkene (0.2 mmol), **Ir4** complex (1 mol%, 0.002 mmol) and 2.0 mL of degassed MeCN (0.1 M). Degasification of the reaction mixture was performed via freeze-pump-thaw cycles (3×10 min under vacuum). Then, the reaction was irradiated and stirred in the photoreactor **setup 1** under

450 nm LED for a specific time depending on the alkene. The solvent was removed under reduced pressure. The residue was dissolved in $CDCl_3$, 1,3,5-trimethoxybenzene (0.065 mmol) was added as an internal standard and a $^1$H NMR spectrum was recorded. The formation of the product was confirmed by comparison of the $^1$H NMR signals with authentic samples and the NMR yield was calculated using the internal standard.

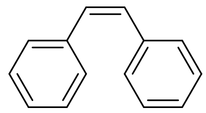

***cis*-1**

Following general procedure B for 24 h, 36.0 mg of *trans*-stilbene **1** (0.2 mmol) gave product *cis*-stilbene **1** in an 83% $^1$H NMR yield. **$^1$H NMR** (300 MHz, $CDCl_3$) δ (ppm) 7.20-7.08 (m, 10H, $CH_{(Ph)}$) and 6.52 (s, 2H, $CH_{(C=C)}$). The obtained spectroscopic data matched the signals of an authentic sample.

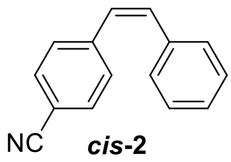

***cis*-2**

Following general procedure B for 24 h, 42.9 mg of ***trans*-2** (0.2 mmol) gave product ***cis*-2** in a 41% $^1$H NMR yield. **$^1$H NMR** (300 MHz, $CDCl_3$) δ (ppm) 7.96 (m, 2H, $CH_{(Ph)}$), 7.50 (m, 1H, $CH_{(Ph)}$), 7.44-7.37 (m, 4H, $CH_{(Ph)}$), 7.26-7.19 (m, 2H, $CH_{(Ph)}$), 7.02 (d, $J$ = 12.9 Hz, 1H, $CH_{(C=C)}$) and 6.62 (d, $J$ = 12.9 Hz, 1H, $CH_{(C=C)}$). Spectroscopic data matched reported data for this compound.[8]

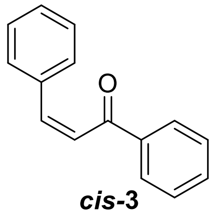

***cis*-3**

Following general procedure B for 2 h, 41.1 mg of *trans*-**3** (0.2 mmol) gave product *cis*-**3** in a 47% $^1$H NMR yield. **$^1$H NMR** (300 MHz, $CDCl_3$) δ (ppm): 7.48 (d, $J$ = 8.1 Hz, 2H, $CH_{(Ph)}$), 7.35-7.14 (m, 8H, $CH_{(Ph)}$), 6.76 (d, $J$ = 12.3 Hz, 1H, $CH_{(C=C)}$) and 6.56 (d, $J$ = 12.3 Hz, 1H, $CH_{(C=C)}$). Spectroscopic data matched reported data for this compound.[9]

## 4.2 General procedure C for dehalogenation reactions

### 4.2.1. General procedure C1: Dehalogenation reactions of Csp$^3$ halides

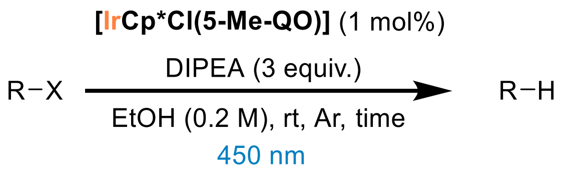


A vial equipped with a magnetic stir bar was charged with the corresponding halide (0.2 mmol), **Ir2** complex (1 mol%, 0.002 mmol) and 1.0 mL of EtOH (0.2 M). Degasification of the reaction mixture was performed via freeze-pump-thaw cycling (3×10 min under vacuum). Then, the reaction was irradiated and stirred in the photoreactor **setup 2** under 450 nm LED for 5 or 16 hours. The crude mixture was filtered through a silica pad and washed with 1 mL of EtOH. Finally, the yield was measured by GC or by $^1$H NMR using 1,3,5-trimethoxybenzene (0.065 mmol) as internal standard comparing the retention time or the $^1$H NMR signals with authentic samples.

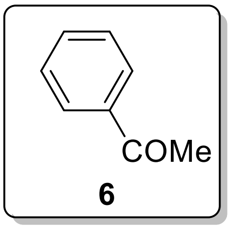


Following general procedure C1 for 16 h, 39.8 mg of 2-bromoacetophenone **5** (0.2 mmol) gave product **6** in a 76% GC yield.

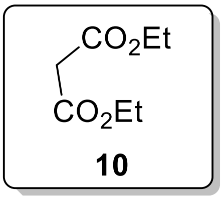


Following general procedure C1 for 5 h, 47.8 mg of 2-diethyl bromomalonate **9** (0.2 mmol) gave product **10** in a 93% $^1$H NMR. **$^1$H NMR** (300 MHz, $CDCl_3$) δ (ppm): 4.20 (q, $J$ = 7.1 Hz, 2H, $CH_{2(COOEt)}$), 3.35 (s, 1H, $CH_2$), 1.28 (t, $J$ = 7.1 Hz, 6H, $CH_{3(COOEt)}$).

### 4.2.2. General procedure C1: Dehalogenation reactions of Csp$^2$ halides

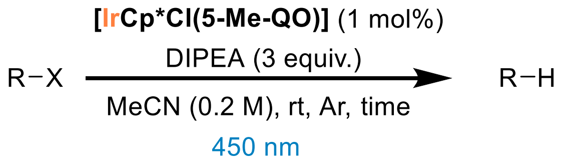


A vial equipped with a magnetic stir bar was charged with the corresponding halide (0.2 mmol), **Ir2** complex (1 mol%, 0.002 mmol) and 1.0 mL of MeCN (0.2 M). Degasification of the reaction mixture was performed via freeze-pump-thaw cycles (3×10 min under vacuum). Then, the reaction was irradiated and stirred in the photoreactor **setup 1** under 450 nm LED up to 72 hours. Without further purification, the final products were confirmed by comparing signals with authentic samples. 1,3,5-trimetoxybenzene (0.065 mmol) as internal standard was added to the reaction mixture and the yield was measured by $^1$H NMR.

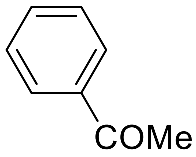


**6**

Following general procedure C2 for 72 h, 39.8 mg of 4′-bromoacetophenone **13** (0.2 mmol) gave product **6** in a 85% $^1$H NMR yield. **$^1$H NMR** (300 MHz, $CDCl_3$) δ (ppm): 7.94 – 7.77 (m, 2H, $CH_{(Ph)}$), 7.56 – 7.48 (m, 1H, $CH_{(Ph)}$), 7.47 – 7.32 (m, 2H, $CH_{(Ph)}$), 2.48 (s, 3H, $CH_3$).

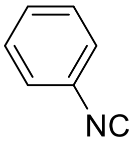


**12**

Following general procedure C2 for 72 h, 36.4 mg of 4-bromobenzonitrile **11** (0.2 mmol) gave product **12** in a 78% $^1$H NMR yield. **$^1$H NMR** (300 MHz, $CDCl_3$) δ (ppm): 7.72 – 7.67 (m, 2H, $CH_{(Ph)}$), 7.66 – 7.59 (m, 1H, $CH_{(Ph)}$), 7.57 – 7.47 (m, 2H, $CH_{(Ph)}$).

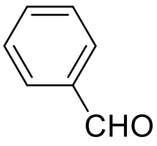


**15**

Following general procedure C2 for 72 h, 37.0 mg of 4-bromobenzaldehyde **14** (0.2 mmol) gave final product **15** in a 43% $^1$H NMR yield. **$^1$H NMR** (300 MHz, $CDCl_3$) δ (ppm): 10.02 (s, 1H, $CH_{(CHO)}$),

7.94 – 7.82 (m, 2H, $CH_{(Ph)}$), 7.69 – 7.57 (m, 1H, $CH_{(Ph)}$), 7.57 – 7.44 (m, 2H, $CH_{(Ph)}$).

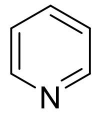

**17**

Following general procedure C2 for 72 h, 23.8 µL of 2-bromopyridine **16** (0.2 mmol) gave product **17** in a 41% $^1$H NMR yield. **$^1$H NMR** (300 MHz, $CDCl_3$) δ (ppm): 8.60 (m, 2H, $CH_{(Py)}$), 7.65 (m, 1H, $CH_{(Py)}$), 7.32 – 7.20 (m, 2H, $CH_{(Py)}$).

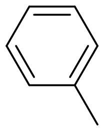

**19**

Following general procedure C2 for 62 h, 25.5 µL iodotoluene **18** (0.2 mmol) gave product **19** in a 98% $^1$H NMR yield. **$^1$H NMR** (300 MHz, $CDCl_3$) δ (ppm): 7.16-7.00 (m, 5H, $CH_{(Ph)}$) and 2.23 (s, 3H, $CH_3$).

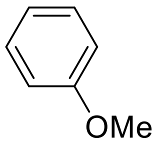


**21**

Following general procedure C2 for 72 h, 23.8 µL 1-iodo-3-methoxybenzene **20** (0.2 mmol) gave product **21** in a 95% $^1$H NMR yield. **$^1$H NMR** (300 MHz, $CDCl_3$) δ (ppm): 7.30 – 7.20 (m, 2H, $CH_{(Ph)}$), 6.86 (m, 3H, $CH_{(Ph)}$) and 3.77 (s, 3H, $CH_3$).

#### 4.2.3. Stability test for Ir6 and Ir7 under photocatalytic conditions

A vial equipped with a magnetic stir bar was charged with 2-bromoacetophenone (0.2 mmol), the corresponding **Ir** complex (5 mol%, 0.01 mmol) and 1.0 mL of EtOH (0.2 M). The reaction mixture was degassed *via* freeze-pump-thaw cycles (3×10 min under vacuum). Then, the reaction was irradiated and stirred in the photoreactor **setup 2** under 450 nm LED for 5. The solvent was evaporated under reduced pressure and the crude mixture was washed with n-pentane (5 x 2 mL) to remove all the organic compounds and dried under vacuum. The resulting solid was analysed by $^1$H NMR spectroscopy comparing its signals with the corresponding ones of pure complex (**Ir6** or **Ir7**) and **Ir1**.

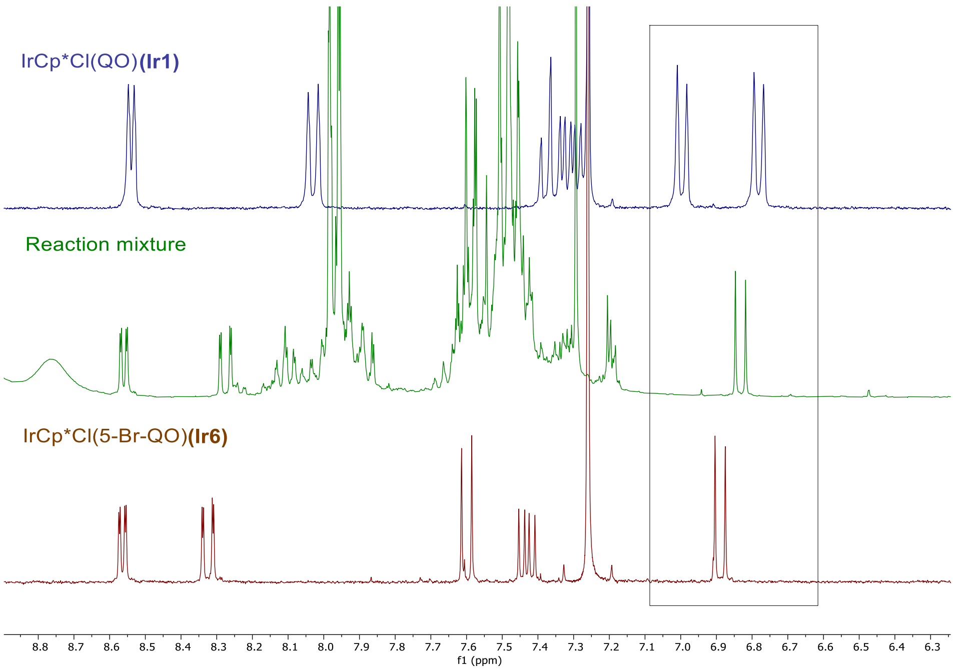


Figure S1. $^{1}$H NMR comparison of **Ir1** (blue), reaction mixture (green) and **Ir6** (red).

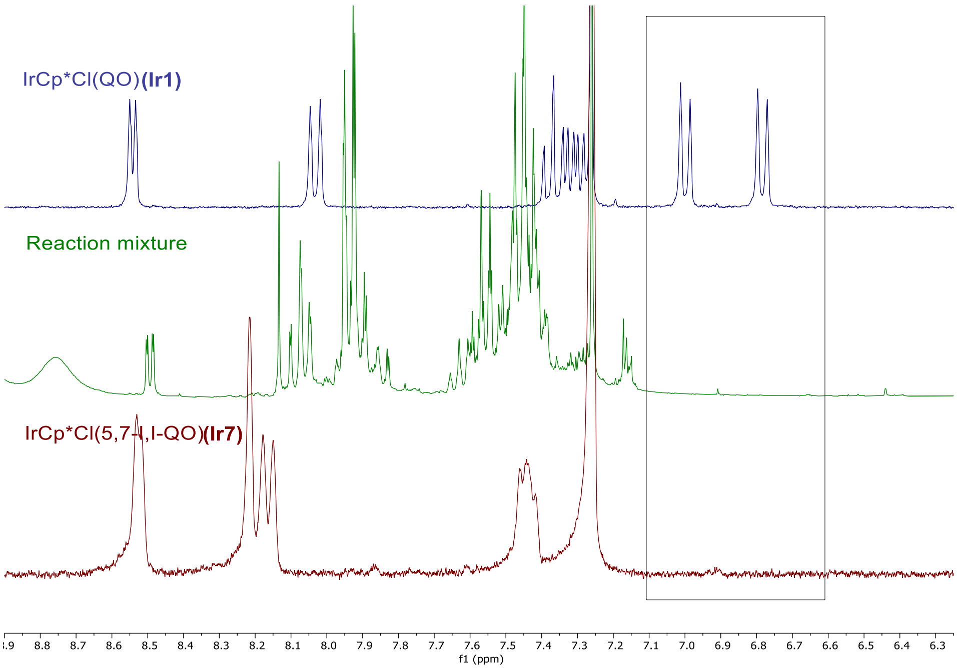


Figure S2. $^{1}$H NMR comparison of **Ir1** (blue), reaction mixture (green) and **Ir7** (red).

## 5. General procedure for transfer hydrogenation reactions

### 5.1. General procedure D for transfer hydrogenation reactions using 2-propanol

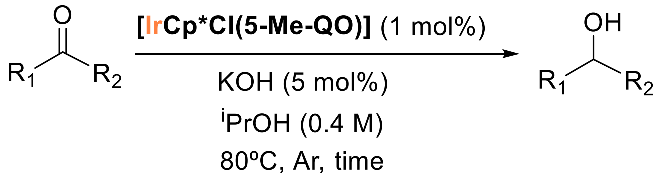


To a 5 mL Schlenk flask the corresponding ketone (1.0 mmol), **Ir2** complex (0.01 mmol, 1 mol%), KOH (0.05 mmol, 5 mol%) in 2.5 mL degassed iPrOH were added under argon. Then, the reaction was stirred at 80 ºC and monitored by $^1$H NMR at different reaction times (24, 48 and 72 hours) comparing signals with authentic samples or reported products. The $^1$H NMR yield was calculated using 1,3,5-trimethoxybenzene (0.10 mmol) as an internal standard.

### 5.2. General procedure E for transfer hydrogenation reactions using formic acid

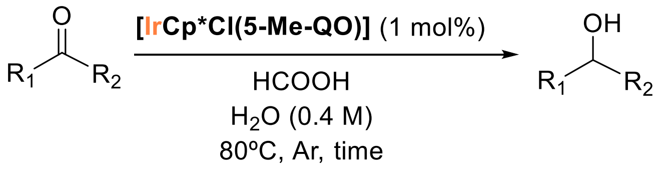


A 10 mL Schlenk flask equipped with a condenser was charged with the corresponding ketone (1.0 mmol), **Ir2** complex (0.01 mmol, 5.21 mg, 1 mol%) and HCOOH (5.0 mmol, 0.4 mL) under argon in 2.1 mL degassed $H_2O$. The resulting suspension was stirred at 80 ºC. The reaction was monitored by $^1$H NMR at different reaction times (24 and 48 hours) comparing the NMR signals with authentic samples or reported products. The $^1$H NMR yield was calculated using 1,3,5-trimethoxybenzene (0.10 mmol) as an internal standard.

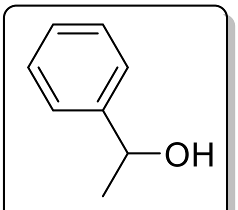


**23**

Following general procedure D for 48 h, 132.4 mg of acetophenone **6** (1.0 mmol) gave product **23** in a 89% $^1$H NMR yield. Following general procedure E for 24 h, product **23** was obtained in a 51% $^1$H NMR yield. **$^1$H NMR** (300 MHz, $CDCl_3$) δ (ppm) 7.27-7.16 (m, 2H, $CH_{(Ph)}$), 7.13-

7.07 (m, 3H, $CH_{(Ph)}$), 5.17 (s, 1H, OH), 4.74 (q, *J* = 6.4 Hz, 1H, CH-OH), 1.34 (d, *J* = 6.4 Hz, 3H, $CH_3$). Spectroscopic data matched the signals of an authentic sample.

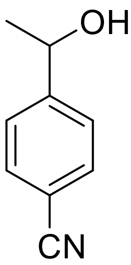


**25**

Following general procedure D for 48 h, 145.3 mg of 4-acetylbenzonitrile **24** (1.0 mmol) gave product **25** in a 99% $^1$H NMR yield. Following general procedure E for 48 h, product **25** was obtained in a 83% $^1$H NMR yield. **$^1$H NMR** (300 MHz, $CDCl_3$) δ (ppm) 7.50 (d, *J* = 7.0 Hz, 2H, $CH_{(Ph)}$), 7.38 (d, *J* = 7.0 Hz, 2H, $CH_{(Ph)}$), 4.79 (q, *J* = 6.3 Hz, 1H, CH-OH), 1.34 (d, *J* = 6.3 Hz, 3H, $CH_3$). Spectroscopic data matched reported data for this compound.[10]

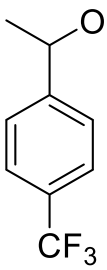


**27**

Following general procedure D for 24 h, 188.2 mg of 4′-trifluoromethylacetophenone **26** (1.0 mmol) gave product **27** in a 99% $^1$H NMR yield. Following general procedure E for 24 h, product **27** was obtained in a 85% $^1$H NMR yield. **$^1$H NMR** (300 MHz, $CDCl_3$) δ (ppm) 7.44 (d, *J* = 8.2 Hz, 2H, $CH_{(Ph)}$), 7.36 (d, *J* = 8.2 Hz, 2H, $CH_{(Ph)}$), 4.77 (q, J = 7.0 Hz, 1H, CH-OH), 1.33 (d, *J* = 7.0 Hz, 3H, $CH_3$). Spectroscopic data matched reported data for this compound.[11]

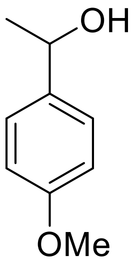


**28**

Following general procedure D for 48 h, 150.5 mg of 4′-methoxyacetophenone **8** (1.0 mmol) gave product **28** in a 10% $^1$H NMR yield. Following general procedure E for 24 h, product **28** was obtained in a 43% $^1$H NMR yield. **$^1$H NMR** (300 MHz, $CDCl_3$) δ (ppm) 7.50 (d, *J* = 7.0 Hz, 2H, $CH_{(Ph)}$), 7.38 (d, *J* = 7.0 Hz, 2H, $CH_{(Ph)}$), 4.79 (q, *J* = 6.3 Hz, 1H, CH-OH), 1.34 (d, *J* = 6.3 Hz, 3H, $CH_3$). Spectroscopic data matched reported data for this compound.[10]

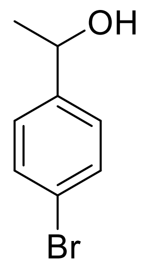

**30**

Following general procedure D for 24 h, 199.0 mg of 4′-bromoacetophenone **29** (1.0 mmol) did not afford the desired alcohol. Following general procedure E for 24 h, product **30** was obtained in a 90% $^{1}$H NMR yield. **$^{1}$H NMR** (300 MHz, $CDCl_3$) δ (ppm) 7.48 – 7.32 (m, 2H, $CH_{(Ph)}$), 7.18 (d, *J* = 8.6 Hz, 2H, $CH_{(Ph)}$), 4.79 (q, *J* = 6.6 Hz, 1H, CH-OH), 1.66 (s, 1H, OH), 1.40 (d, *J* = 6.6 Hz, 3H, $CH_3$). Spectroscopic data matched reported data for this compound.[11]

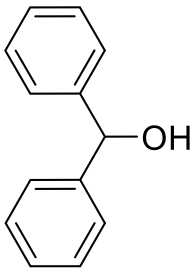

**32**

Following general procedure D for 72 h, 182.3 mg of benzophenone **31** (1.0 mmol) gave product **32** in a 84% $^{1}$H NMR yield. Following general procedure E for 48 h, product **32** was obtained in a 32% $^{1}$H NMR yield. **$^{1}$H NMR** (300 MHz, $CDCl_3$) δ (ppm) 7.42 (d, *J* = 7.2 Hz, 4H, $CH_{(Ph)}$), 7.56-7.36 (m, 6H, $CH_{(Ph)}$), 6.02 (s, 1H, CH-OH). Spectroscopic data matched reported data for this compound.[10]

## 6. General procedure E for one-pot sequential reaction

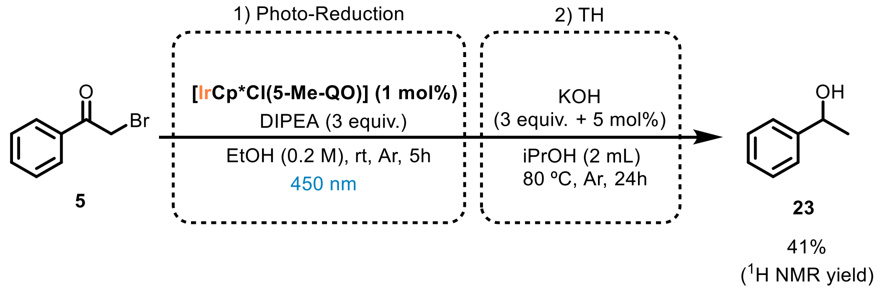


A Schlenk flask equipped with a magnetic stir bar was charged with 39.8 mg of 2-bromoacetophenone **5** (0.2 mmol), **Ir2** complex (1 mol%, 0.002 mmol) and 1.0 mL of EtOH (0.2 M). Degasification of the reaction mixture was performed via freeze-pump-thaw cycling (3×10 min under vacuum). Then, the reaction was irradiated and stirred in the photoreactor **setup 2** under 450 nm LED for 5 hours. The solvent was evaporated under reduced pressure in the schlenk line cooling down the mixture with an ice bath. Subsequently, 34.2 mg KOH (3 equiv + 5 mol%) was added to the crude and then it was dissolved in 2.5 mL degassed iPrOH

under argon atmosphere. Then, the reaction was stirred at 80 °C for 24 hours. Finally, the yield was measured by $^{1}H$ NMR using 1,3,5-trimethoxybenzene as internal standard.

## 7. Optimization of isomerization reactions

Table S1. Screening of different reaction conditions for the isomerization of ***trans*-1**

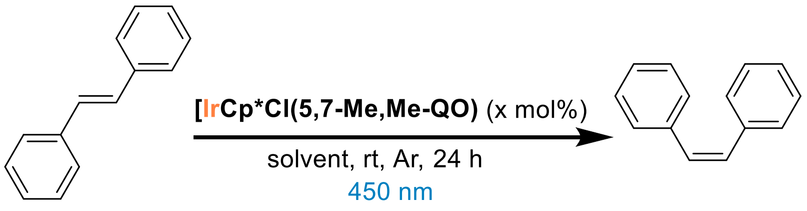


***trans*-1** ***cis*-1**

| Entry | Catalyst loading (mol%) | Concentration (M) | Solvent | NMR yield (%) |
|---|---|---|---|---|
| **1** | 1 | 0.10 | MeCN | 83 |
| **2** | 1 | 0.10 | $CH_2Cl_2$ | 53 |
| **3** | 1 | 0.10 | DMF | 40 |
| **4** | 1 | 0.10 | THF | 40 |
| **5** | 2 | 0.10 | MeCN | 84 |
| **6** | 1 | 0.13 | MeCN | 84 |
| **7** | 1 | 0.07 | MeCN | 57 |

## 8. UV-Vis absorption data for complexes [IrCp*Cl(N^O)]

The absorption spectra of 1.0 mM solutions of the corresponding complexes in degassed $CH_2Cl_2$ were recorder using an Agilent 8453 UV-vis spectrophotometer. Molar extinction coefficients of each complex ($3.29 \cdot 10^{-5}$ M in $CH_2Cl_2$) were calculated using the Beer-Lambert equation: $A = \varepsilon \cdot l \cdot c$

Table S2. Molar extinction coefficient (ε) of each complex ($3.29 \cdot 10^{-5}$ M) in $CH_2Cl_2$.

| Ir Complex | $\lambda_{abs}$ nm/ ε (L/mol·cm) |
|---|---|
| **[IrCp*Cl(QO)] (Ir1)** | 259 (20973), 349 (3835), 430 (3152) |
| **[IrCp*Cl(5-Me-QO)] (Ir2)** | 263 (30556), 357 (6208), 471 (5452) |
| **[IrCp*Cl(5-OMe-QO)] (Ir3)** | 265 (19864), 368 (5041), 485 (3135) |
| **[IrCp*Cl(5,7-Me, Me-QO)] (Ir4)** | 264 (20207), 358 (4460), 475 (3582) |
| **[IrCp*Cl(5-Cl-QO)] (Ir5)** | 262 (22968), 359 (4238), 468 (3394) |
| **[IrCp*Cl(5-Br-QO)] (Ir6)** | 265 (28387), 358 (4225), 465 (4224) |

| | |
|---|---|
| **[IrCp*Cl(5,7-I, I-QO)] (Ir7)** | 277 (22048), 365 (5338), 466 (4467) |
| **[IrCp*Cl(2-Me-QO)] (Ir8)** | 260 (19339), 350 (3308), 420 (2961) |

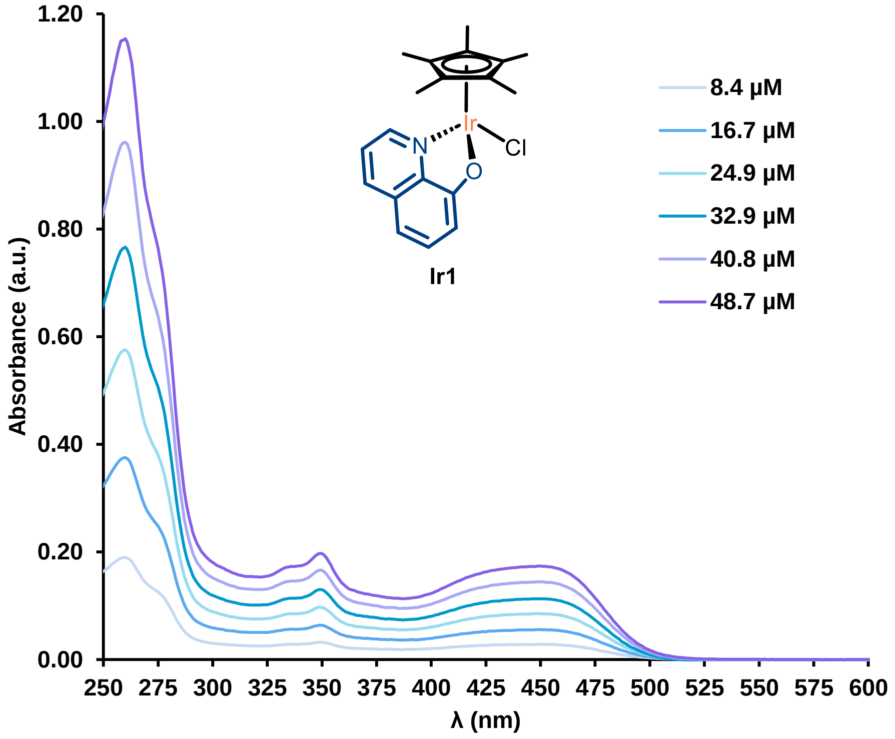


Figure S3. Absorption spectra of complex **Ir1** at different concentrations in degassed $CH_2Cl_2$.

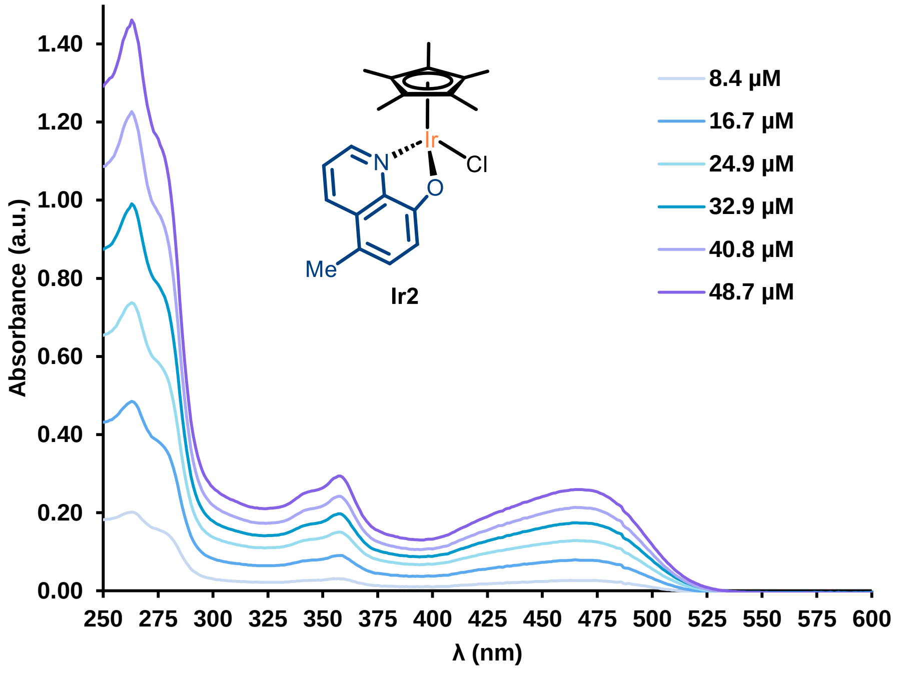


Figure S4. Absorption spectra of complex **Ir2** at different concentrations in degassed $CH_2Cl_2$.

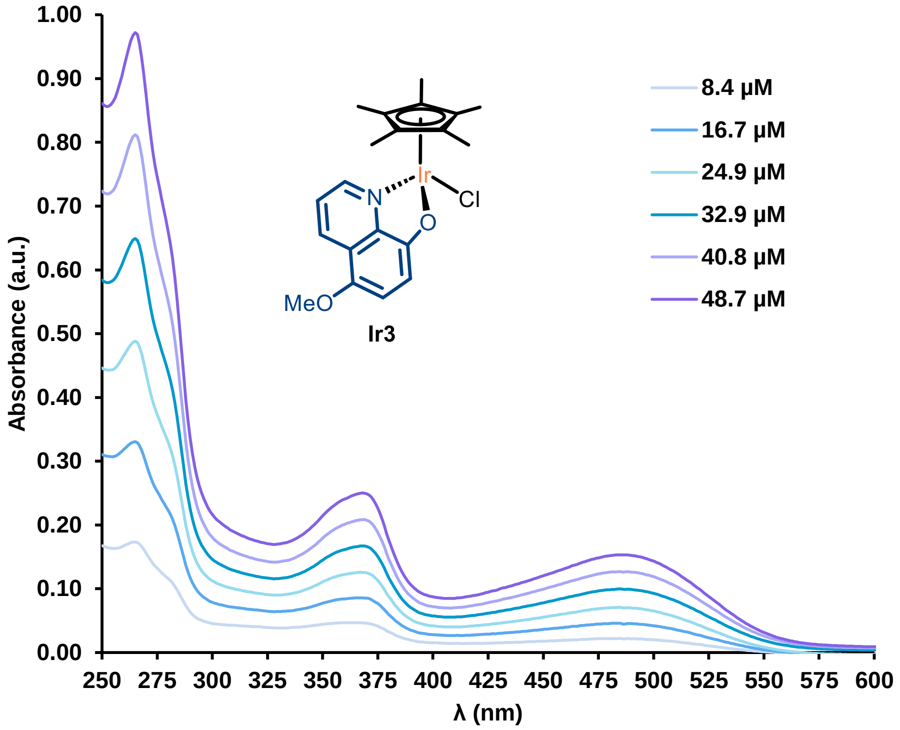


Figure S5. Absorption spectra of complex **Ir3** at different concentrations in degassed $CH_2Cl_2$.

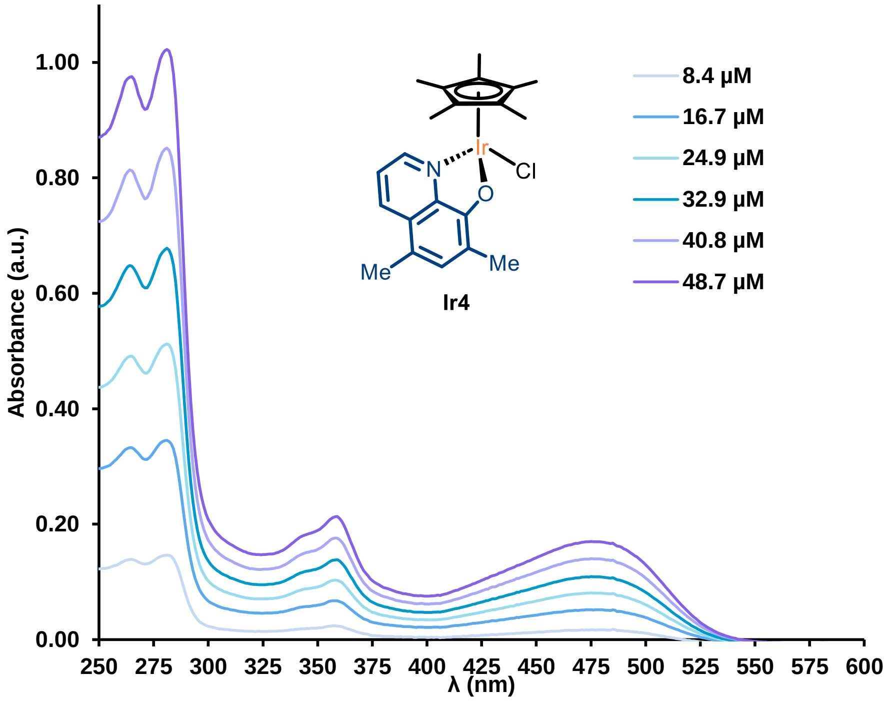


Figure S6. Absorption spectra of complex **Ir4** at different concentrations in degassed $CH_2Cl_2$.

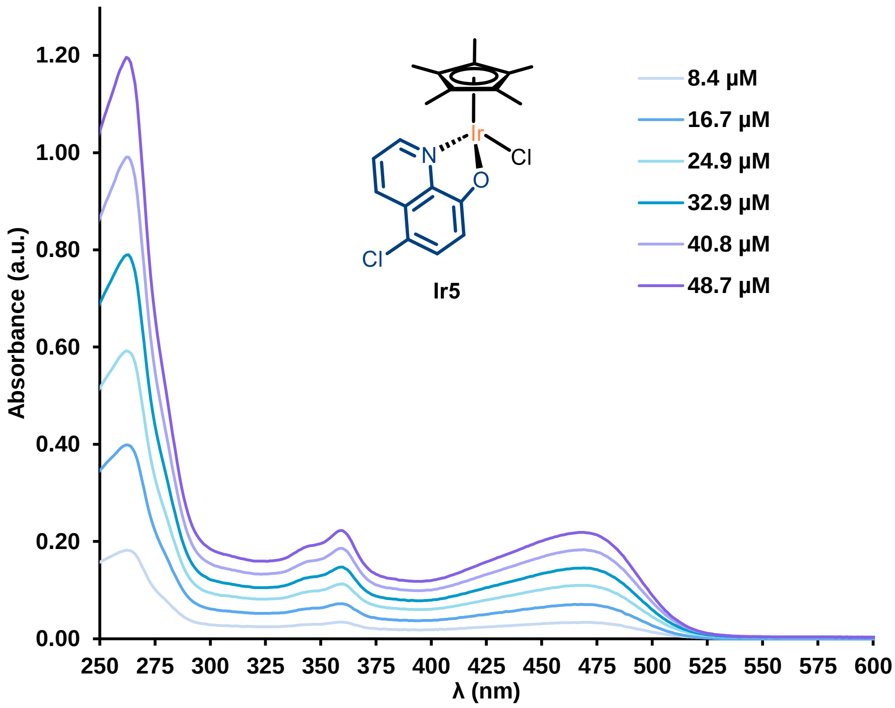


Figure S7. Absorption spectra of complex **Ir5** at different concentrations in degassed $CH_2Cl_2$.

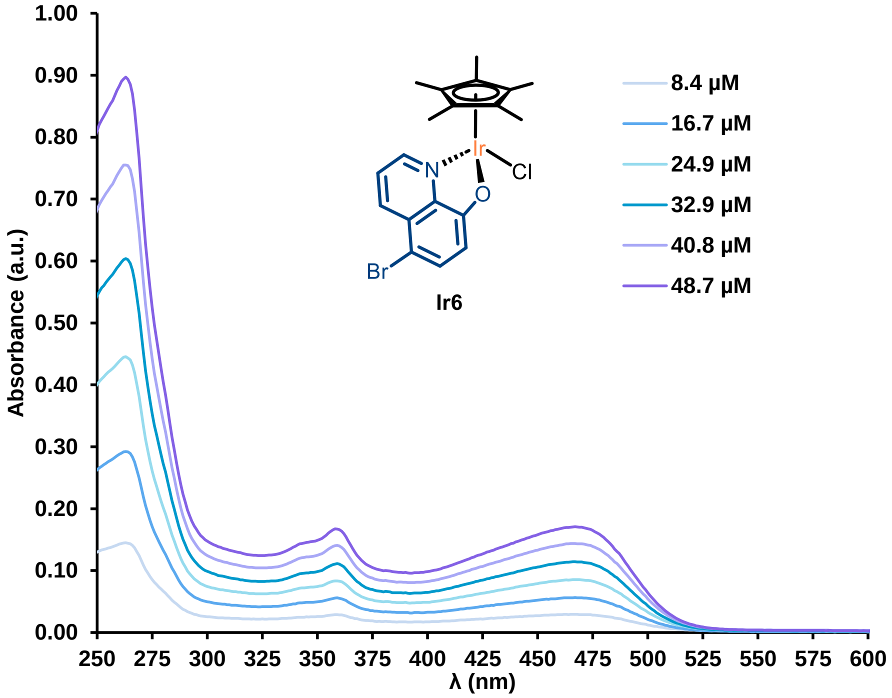


Figure S8. Absorption spectra of complex **Ir6** at different concentrations in degassed $CH_2Cl_2$.

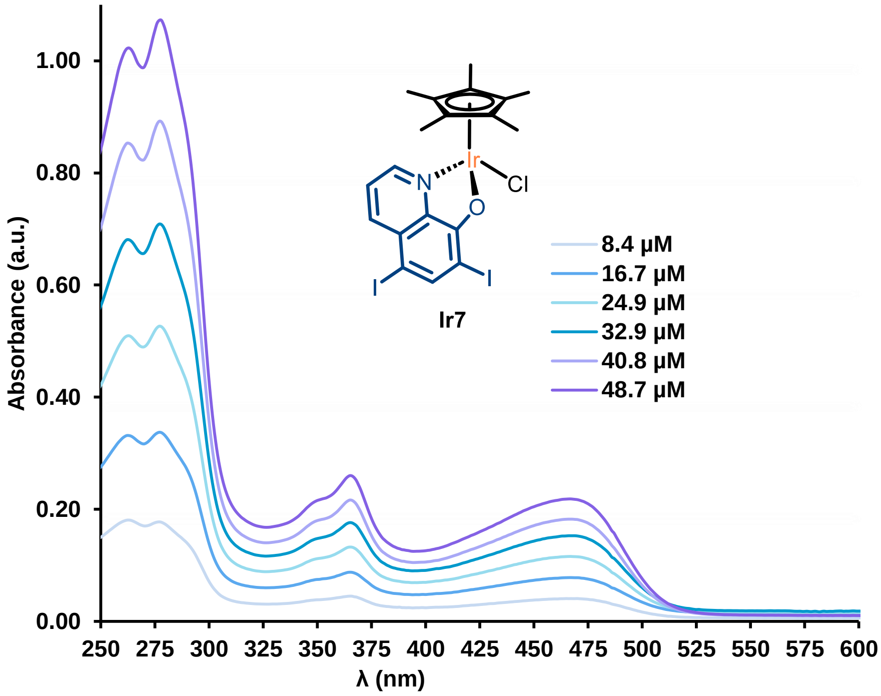


Figure S9. Absorption spectra of complex **Ir7** at different concentrations in degassed $CH_2Cl_2$.

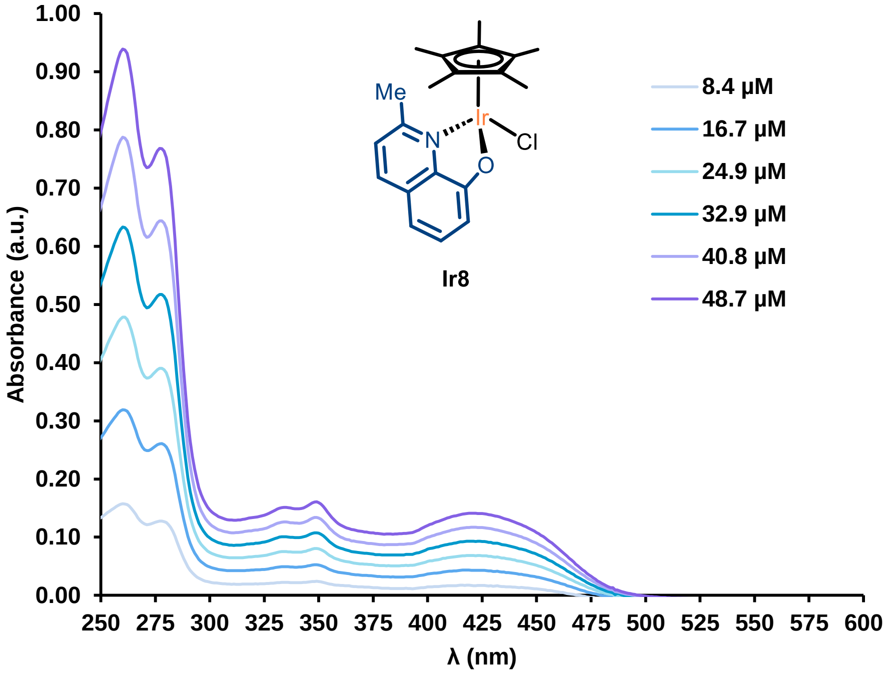


Figure S10. Absorption spectra of complex **Ir8** at different concentrations in degassed $CH_2Cl_2$.

## 9. Emission spectra data for complexes [IrCp*Cl(N^O)]

All the measurements were obtained from acetonitrile media for room temperature emission and glassy 2-MeTHF for low-temperature emission (77 K).

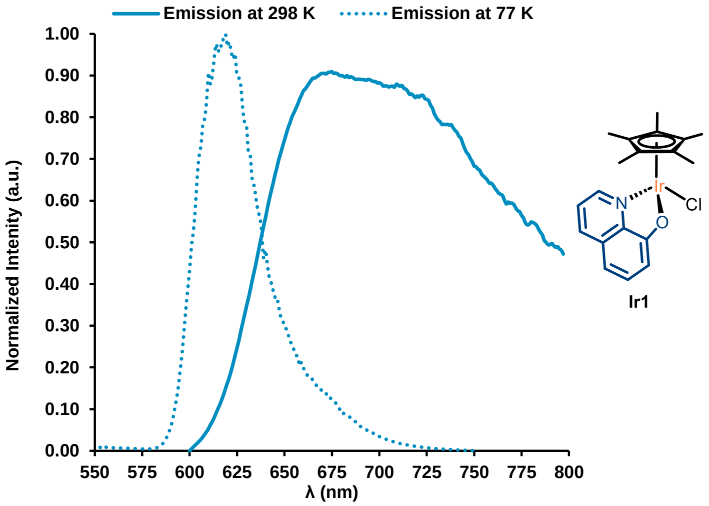


Figure S11. Overlaid room-temperature (298 K, solid line) and low temperature (77 K, dotted line) emission spectra of complex **Ir1**.

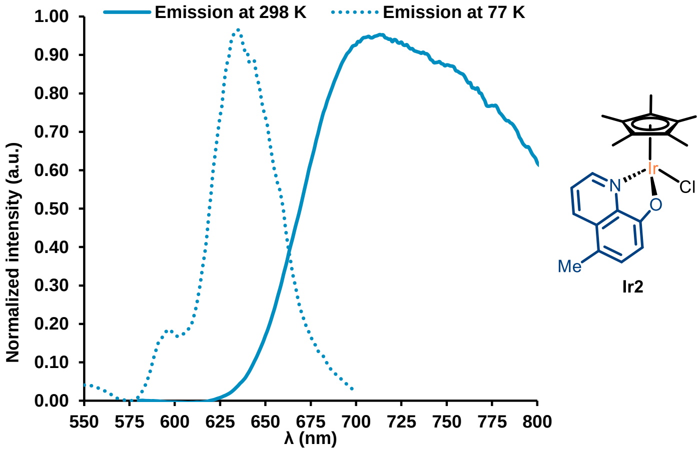


Figure S12. Overlaid room-temperature (298 K, solid line) and low temperature (77 K, dotted line) emission spectra of complex **Ir2**.

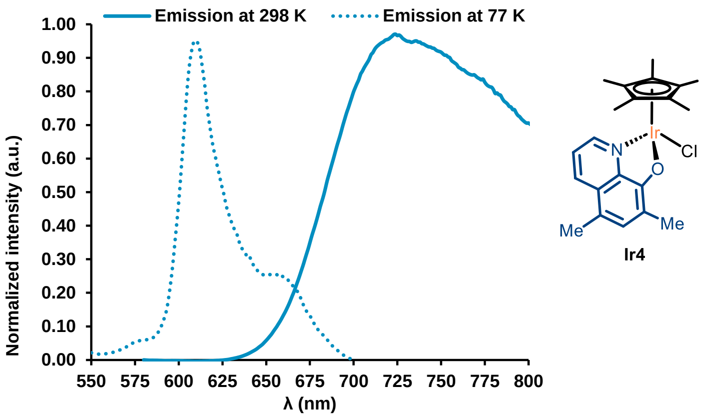


Figure S13. Overlaid room-temperature (298 K, solid line) and low temperature (77 K, dotted line) emission spectra of complex **Ir4**.

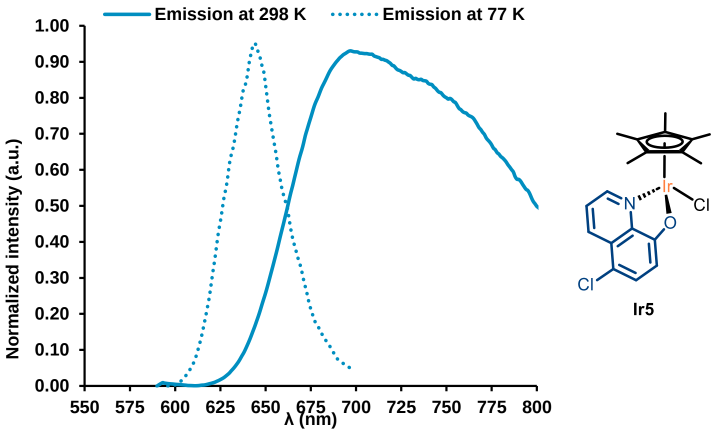


Figure S14. Overlaid room-temperature (298 K, solid line) and low temperature (77 K, dotted line) emission spectra of complex **Ir5**.

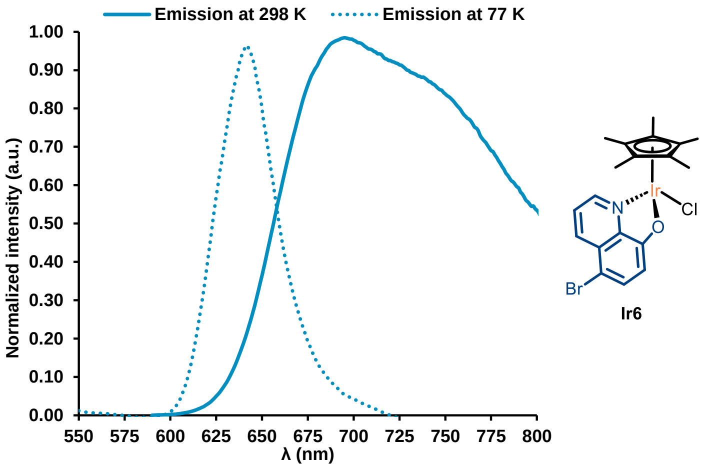


Figure S15. Overlaid room-temperature (298 K, solid line) and low temperature (77 K, dotted line) emission spectra of complex **Ir6**.

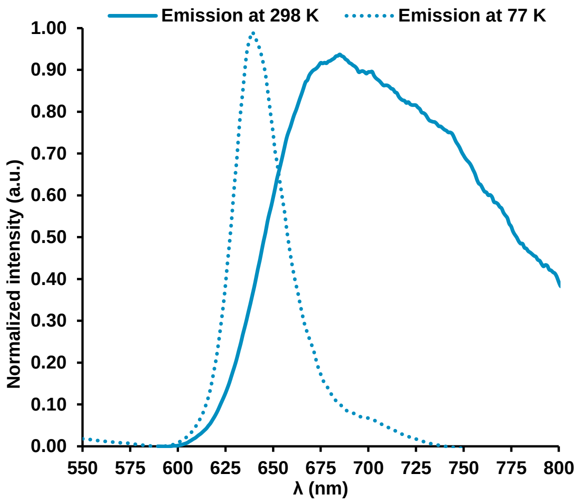


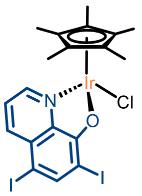

Ir7

Figure S16. Overlaid room-temperature (298 K, solid line) and low temperature (77 K, dotted line) emission spectra of complex **Ir7**.

## 10. Stern-Volmer quenching studies for [IrCp*Cl(N^O)]

All samples were prepared under an argon atmosphere to avoid any quenching by oxygen. Solutions of the iridium complex, DIPEA and bromides were prepared in degassed MeCN. The iridium complex **Ir2** was diluted in a quartz cuvette to make a solution with an absorption of 0.15 at 450 nm under argon atmosphere ($\lambda_{exc}$ = 450 nm). The concentration of the quencher stock solution was 0.31 M and aliquots of 25 µL were introduced to the cuvette.

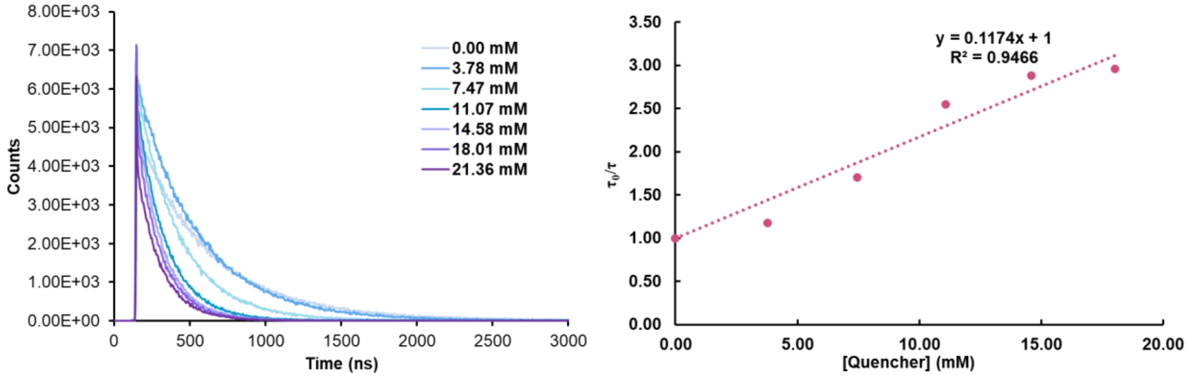


Figure S17. Time resolved emission quenching of **Ir2** photocatalyst with increasing concentrations of DIPEA and Stern-Volmer plot to obtain $k_q$.

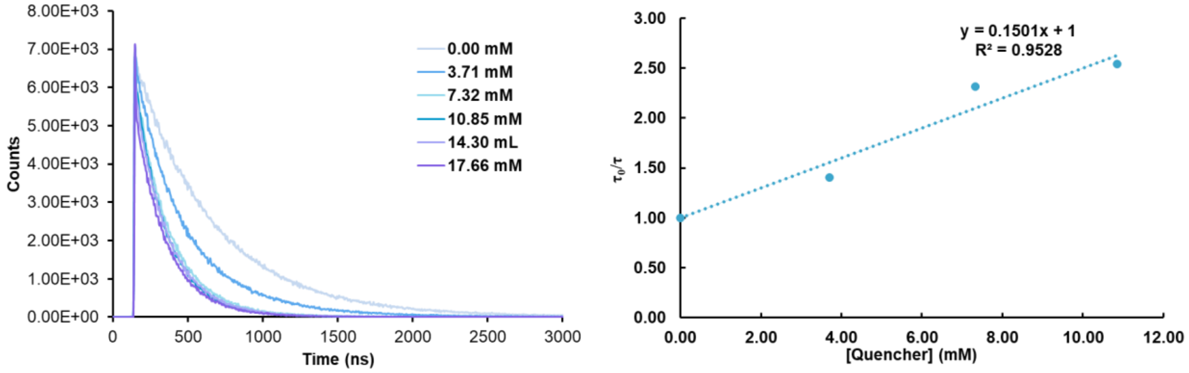


Figure S18. Time resolved emission quenching of **Ir2** photocatalyst with increasing concentrations of **5** and Stern-Volmer plot to obtain $k_q$.

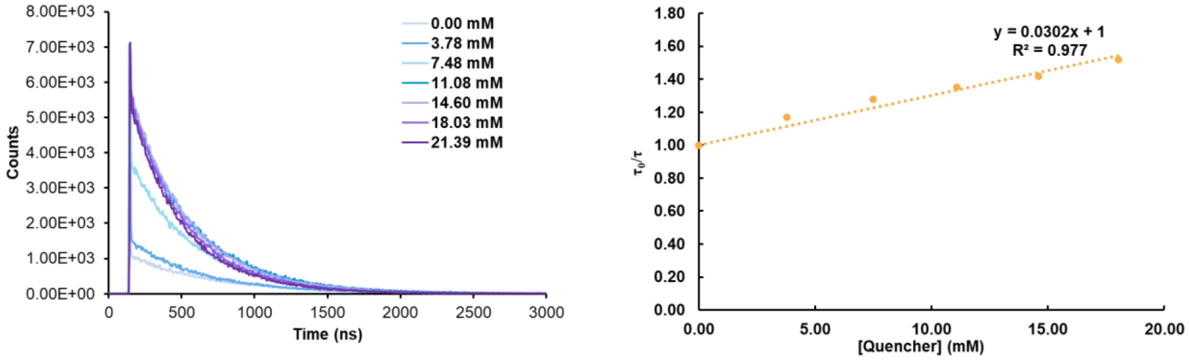


Figure S19. Time resolved emission quenching of **Ir2** photocatalyst with increasing concentrations of **11** and Stern-Volmer plot to obtain $k_q$.

Table S3. Determination of the quenching constant $k_q$.

| | **$K_{sv}$ ($mM^{-1}$)** | **τ (ns)** | **$k_q$ ($M^{-1}\cdot s^{-1}$)[a]** |
|---|---|---|---|
| **Ir2 + 5** (2-bromoacetophenone) | 0.15014 | 525.14 | $2.86\cdot10^8$ |
| **Ir2 + DIPEA** | 0.11745 | 481.22 | $2.44\cdot10^8$ |
| **Ir2 + 11** (4-Bromobenzonitrile) | 0.02795 | 587.16 | $4.76\cdot10^7$ |

[a]The formula used for the calculation is: $K_{sv}=k_q\cdot\tau$

## 11. Electrochemistry

Electrochemical measurements were performed in a Metrohm Autolab Potentiostat model PGSTAT204. A three-electrode cell including a 3 mm-diameter glassy carbon working electrode, a platinum wire counterelectrode and a Ag/AgCl reference electrode was employed. Cyclic voltammograms of complexes **Ir1-Ir8** were recorded under argon, in acetonitrile ($10^{-3}$ M), using $[nBu_4]PF_6$ ($10^{-1}$ M) as supporting electrolyte at 20 ˚C at a 100 mV/s scan rate, unless otherwise specified. Potentials (V) are relative to the Ag/AgCl electrode. Measured potentials were calibrated using an internal $Fc/Fc^+$ standard.

## 11.1 Cyclic voltammograms (CV) of Ir1-Ir8

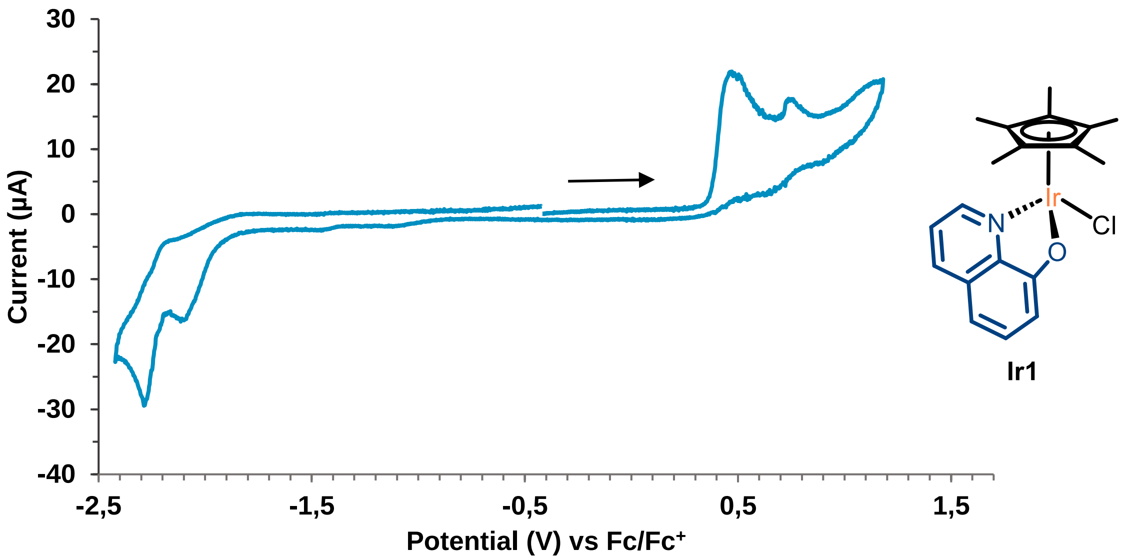


Figure S20. CV of complex **Ir1**

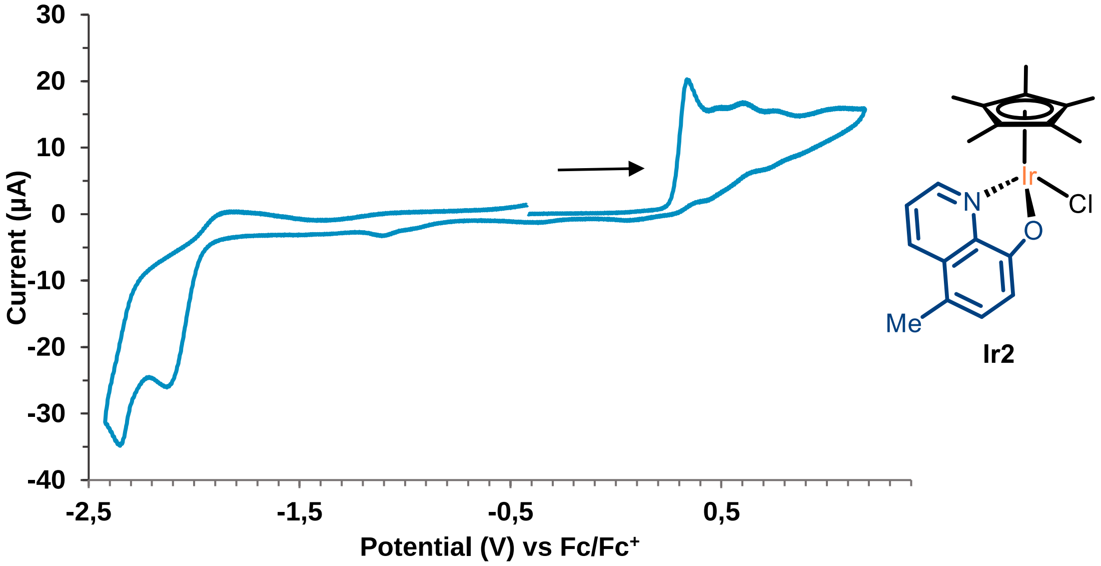


Figure S21. CV of complex **Ir2**

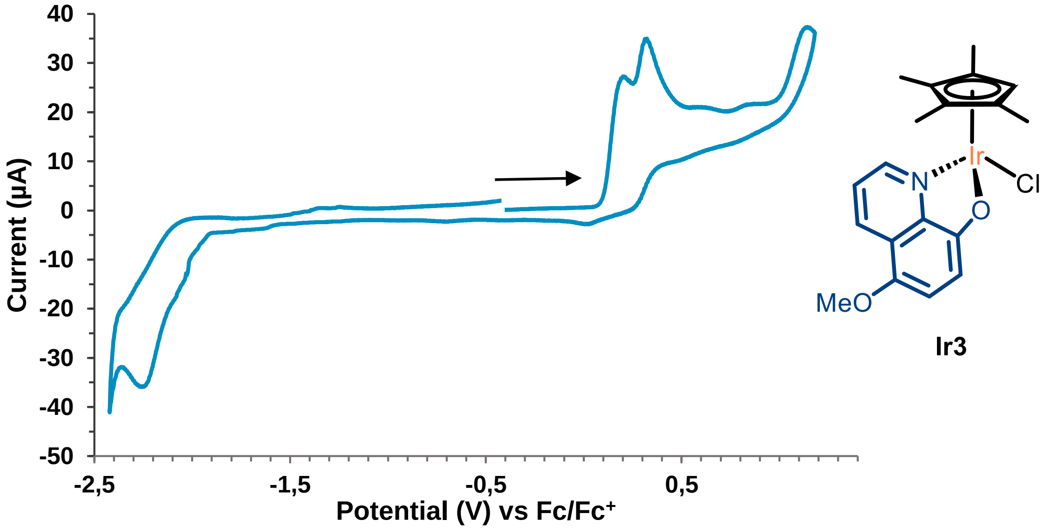


Figure S22. CV of complex **Ir3**

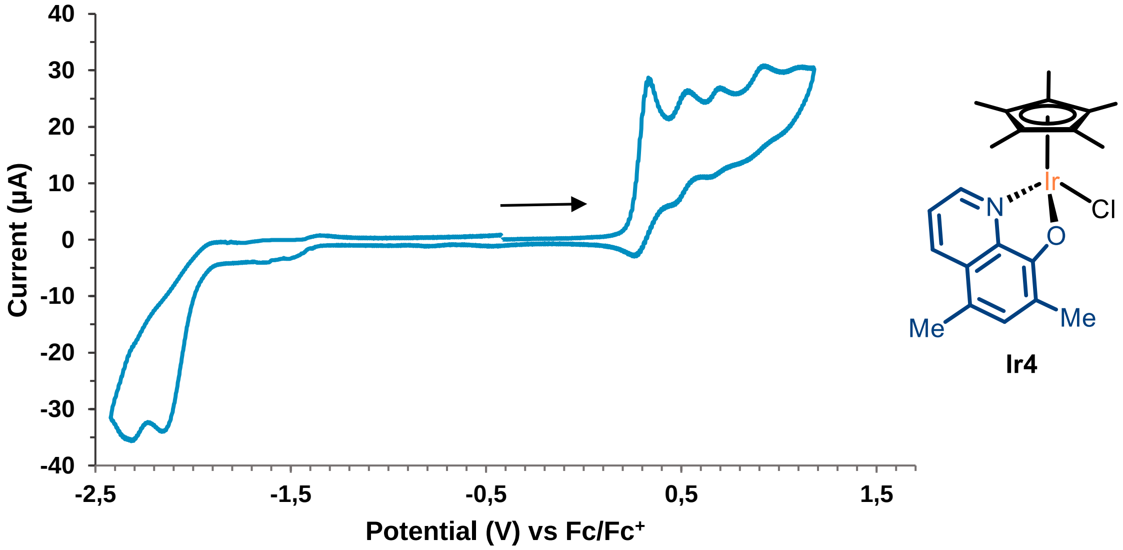


Figure S23. CV of complex **Ir4**

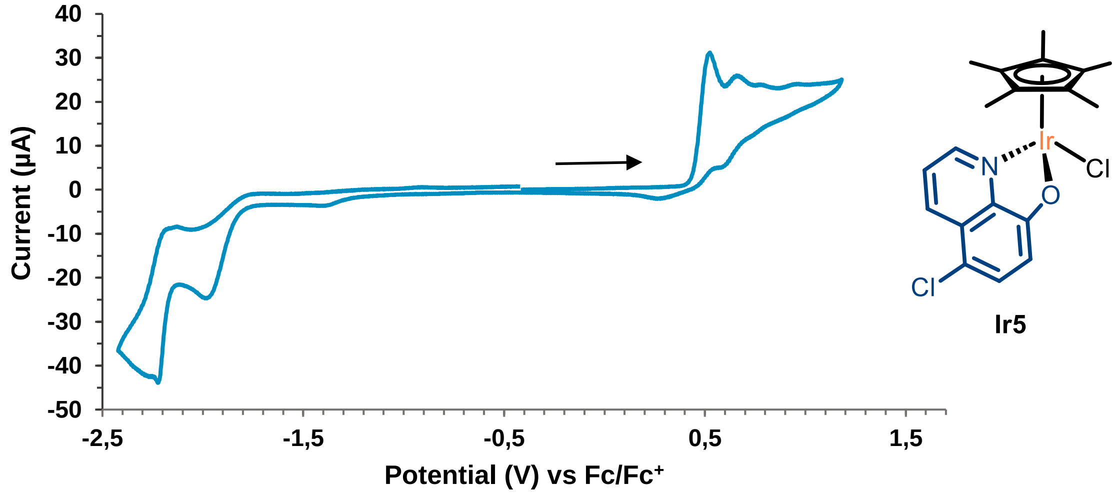


Figure S24. CV of complex **Ir5**

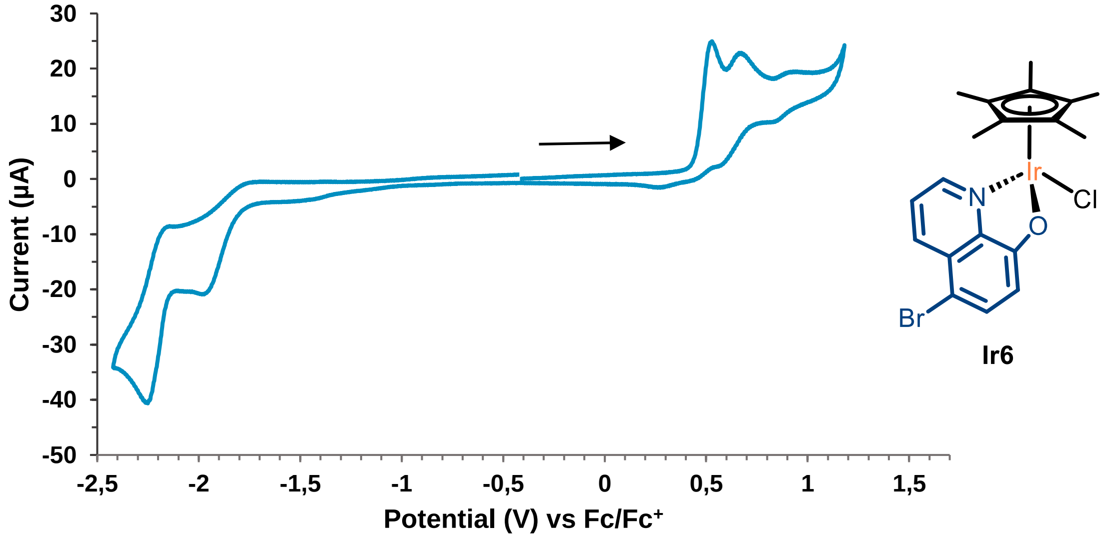


Figure S25. CV of complex **Ir6**

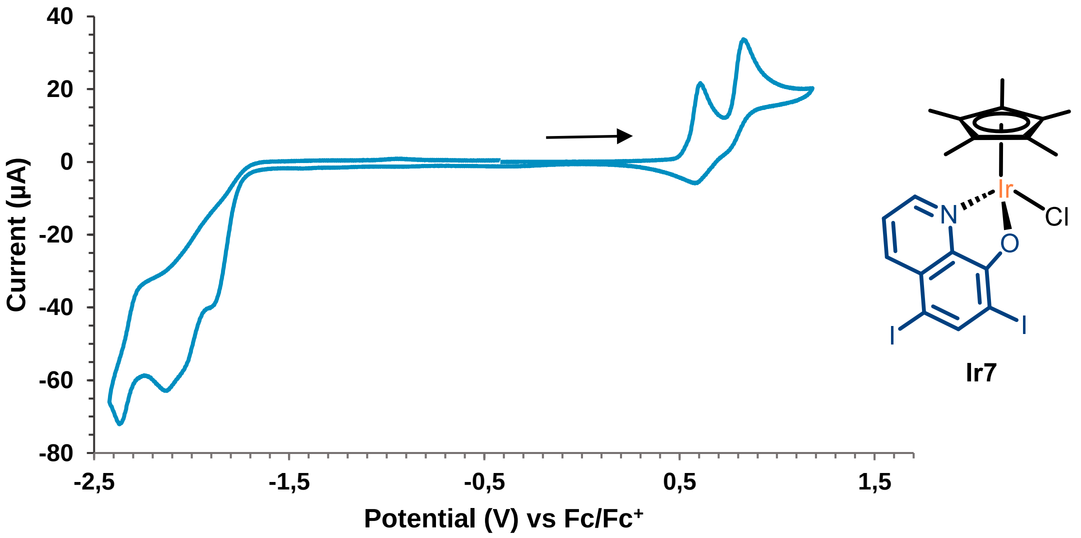


Figure S26. CV of complex **Ir7**

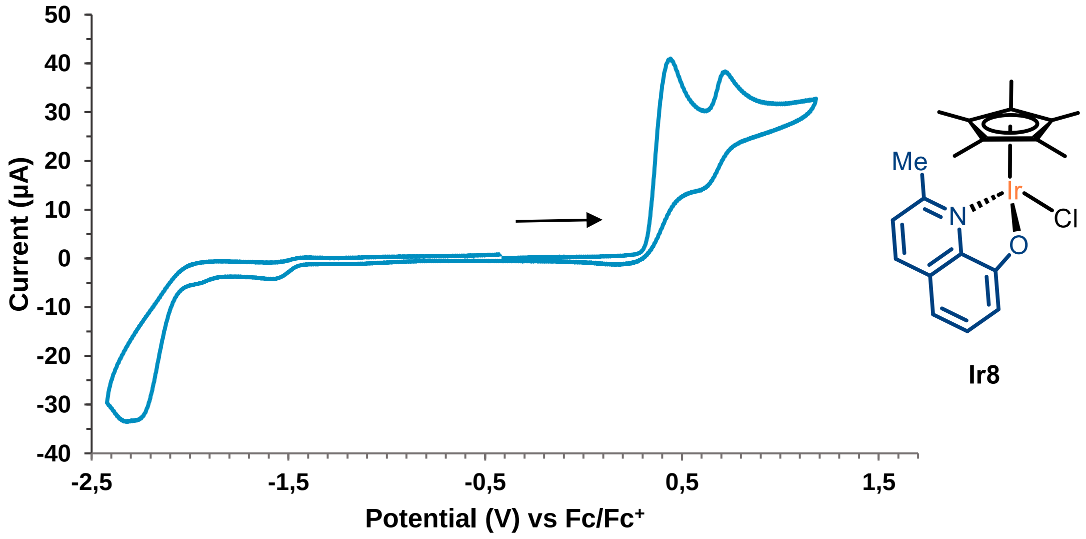


Figure S27. CV of complex **Ir8**

## 11.2 Scan rate dependence of first oxidation process of Ir1-Ir8

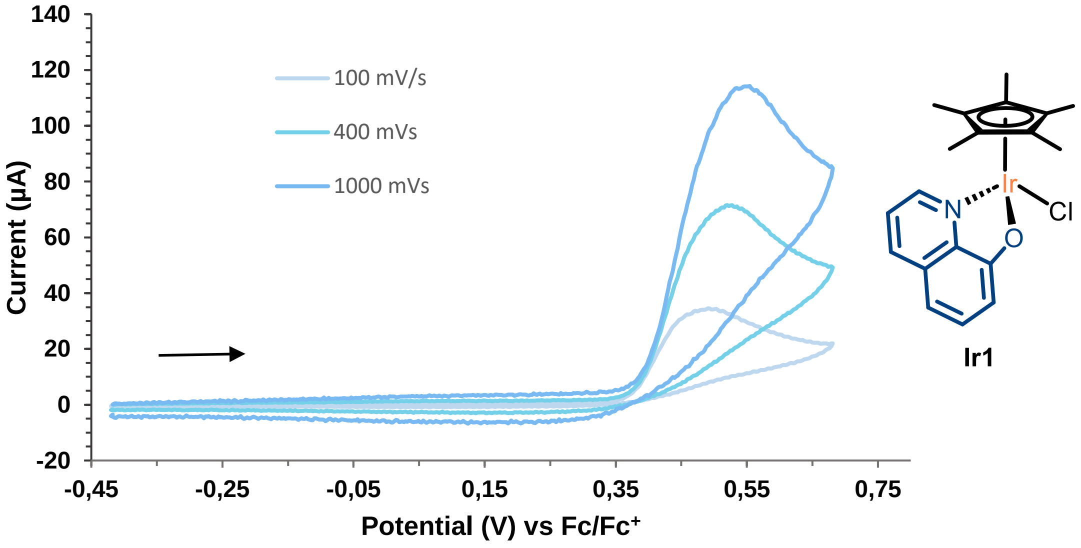


Figure S28. CV of complex **Ir1** at different scan rates

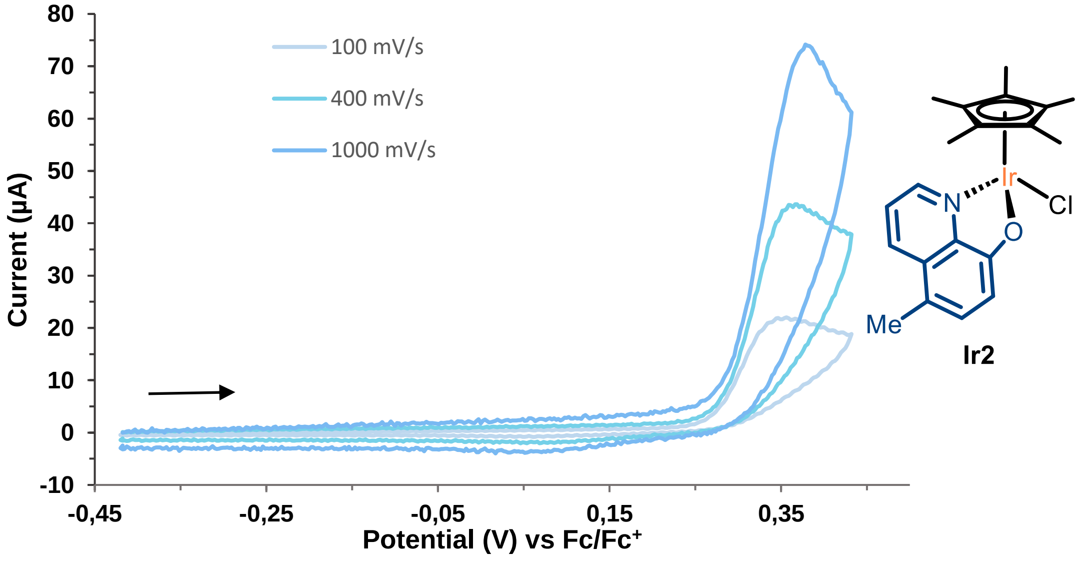


Figure S29. CV of complex **Ir2** at different scan rates

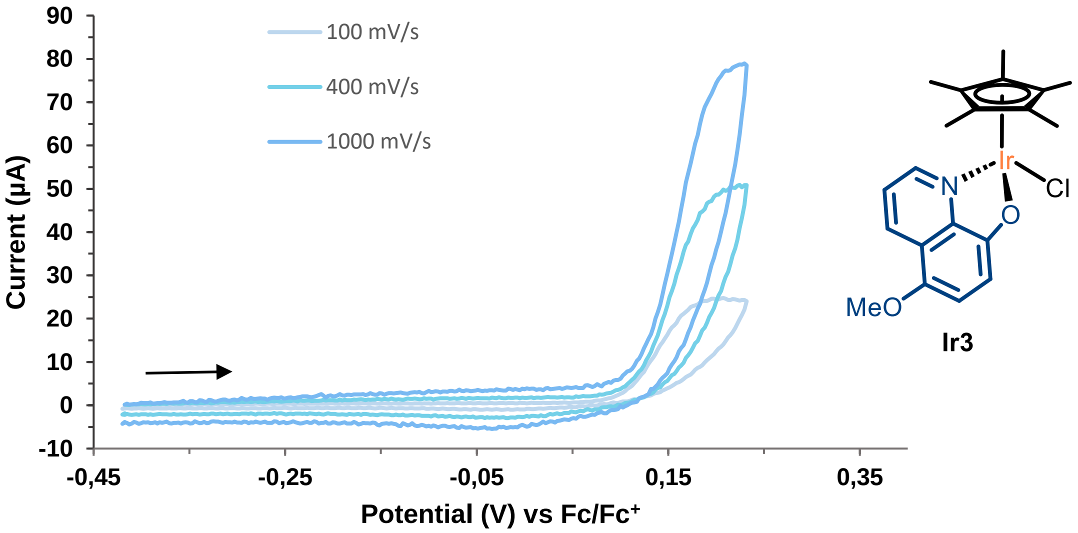


Figure S30. CV of complex **Ir3** at different scan rates

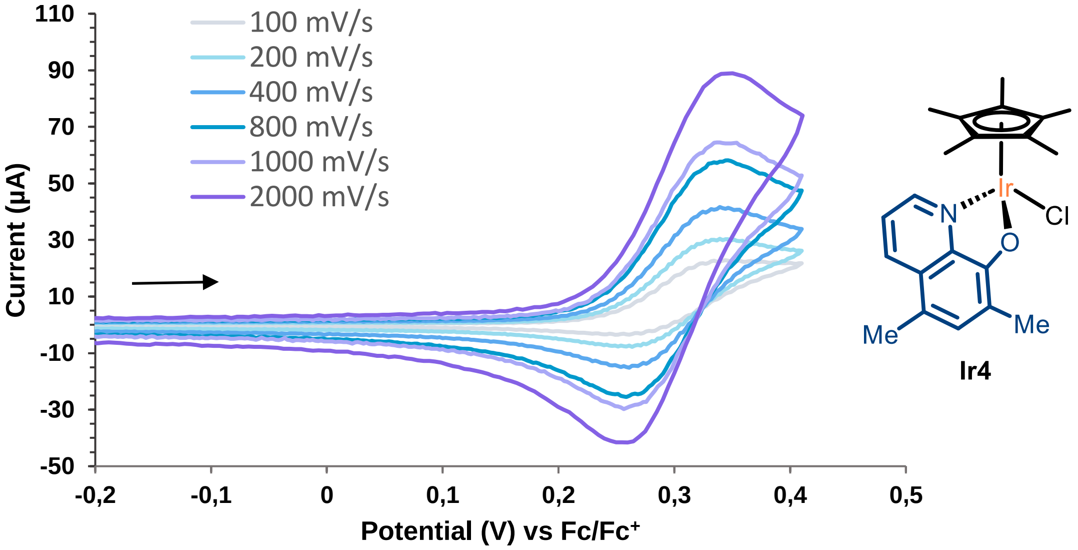


Figure S31. CV of complex **Ir4** at different scan rates

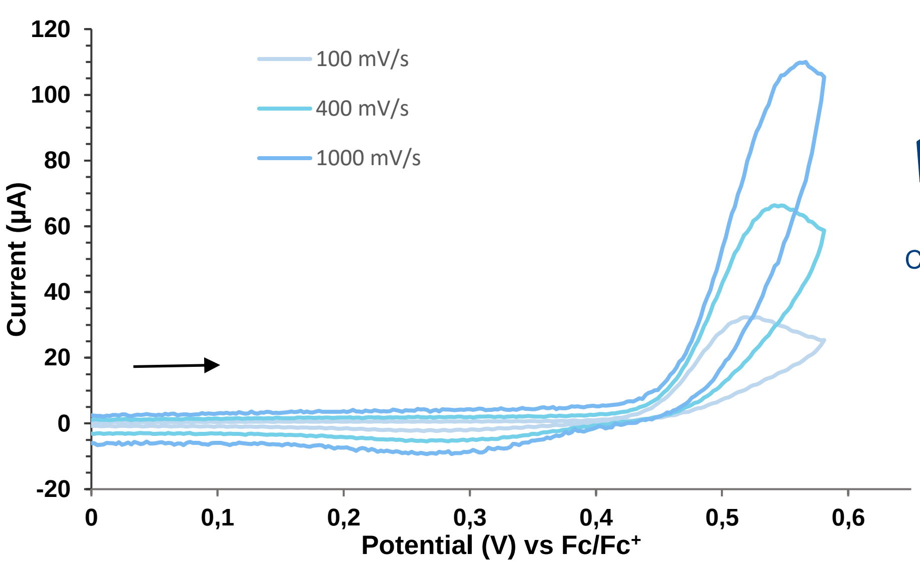


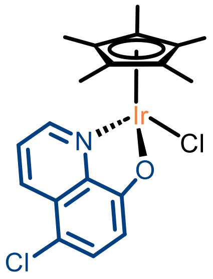


**Ir5**

Figure S32. CV of complex **Ir5** at different scan rates

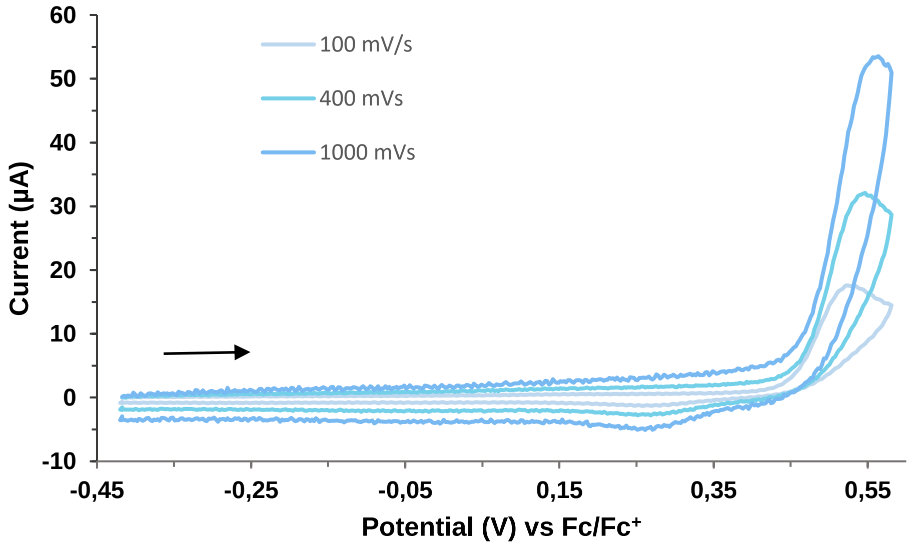


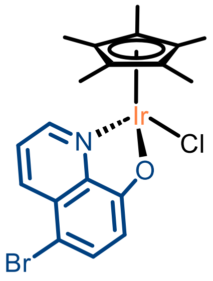


**Ir6**

Figure S33. CV of complex **Ir6** at different scan rates

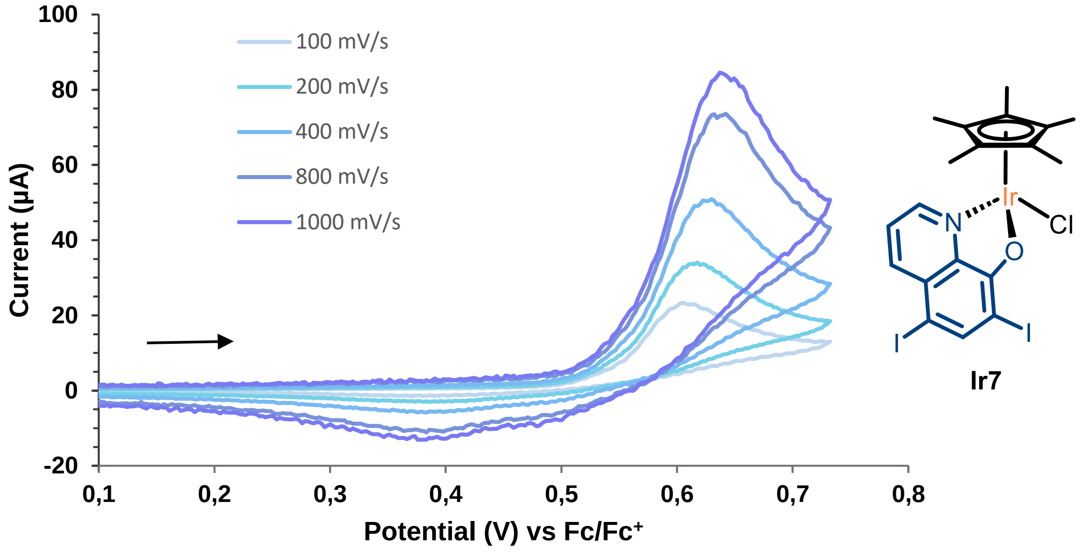


Figure S34. CV of complex **Ir7** at different scan rates

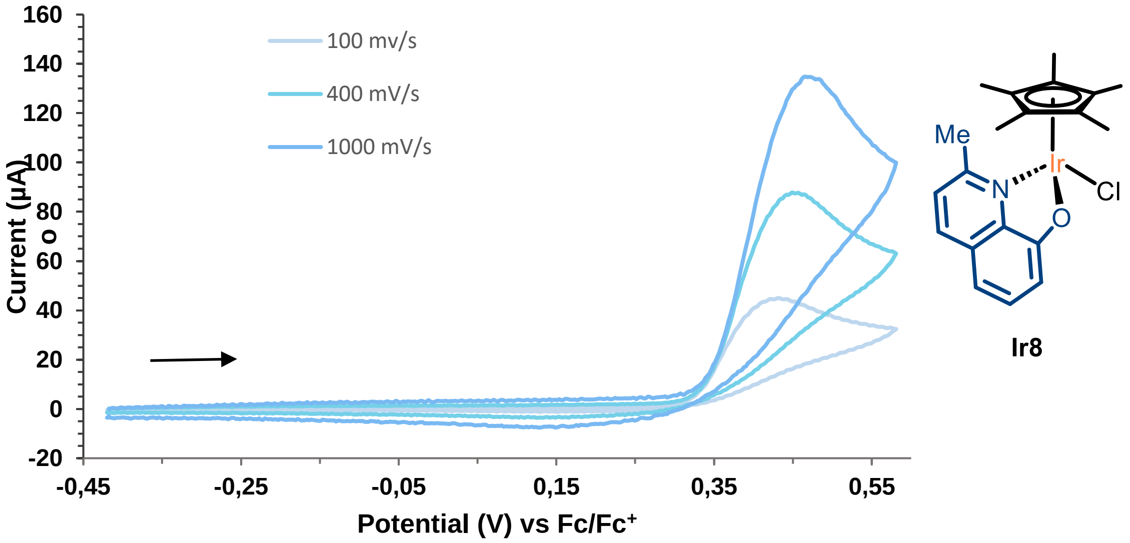


Figure S35. CV of complex **Ir8** at different scan rates

## 12. NMR spectra

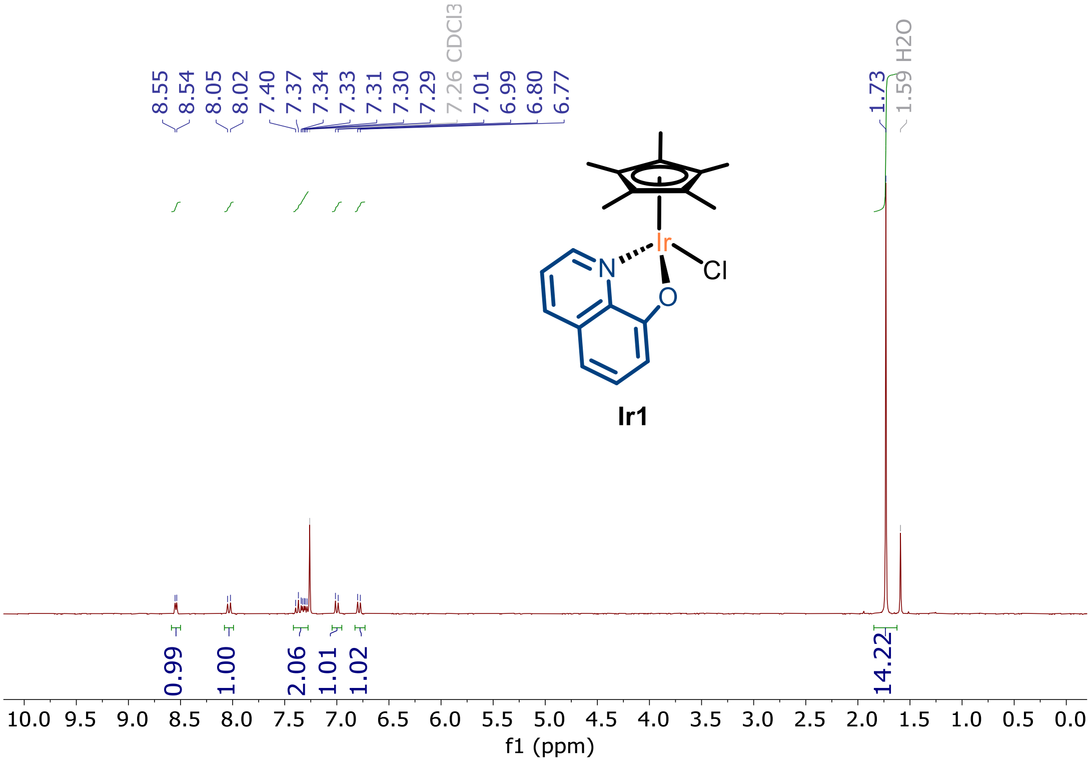


Figure S36. $^{1}$H NMR spectrum of **Ir1** in $CDCl_3$ (300 MHz)

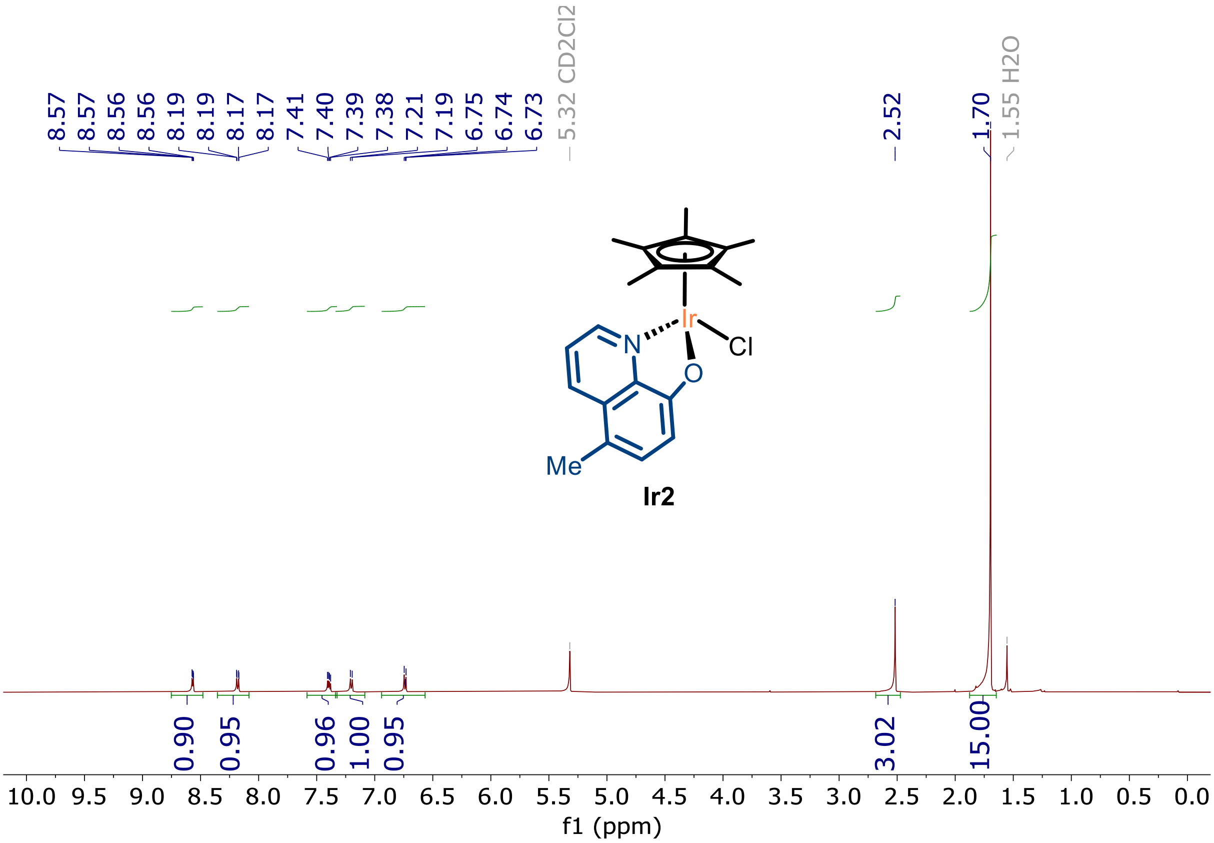


Figure S37. $^{1}$H NMR spectrum of **Ir2** in $CD_2Cl_2$ (500 MHz)

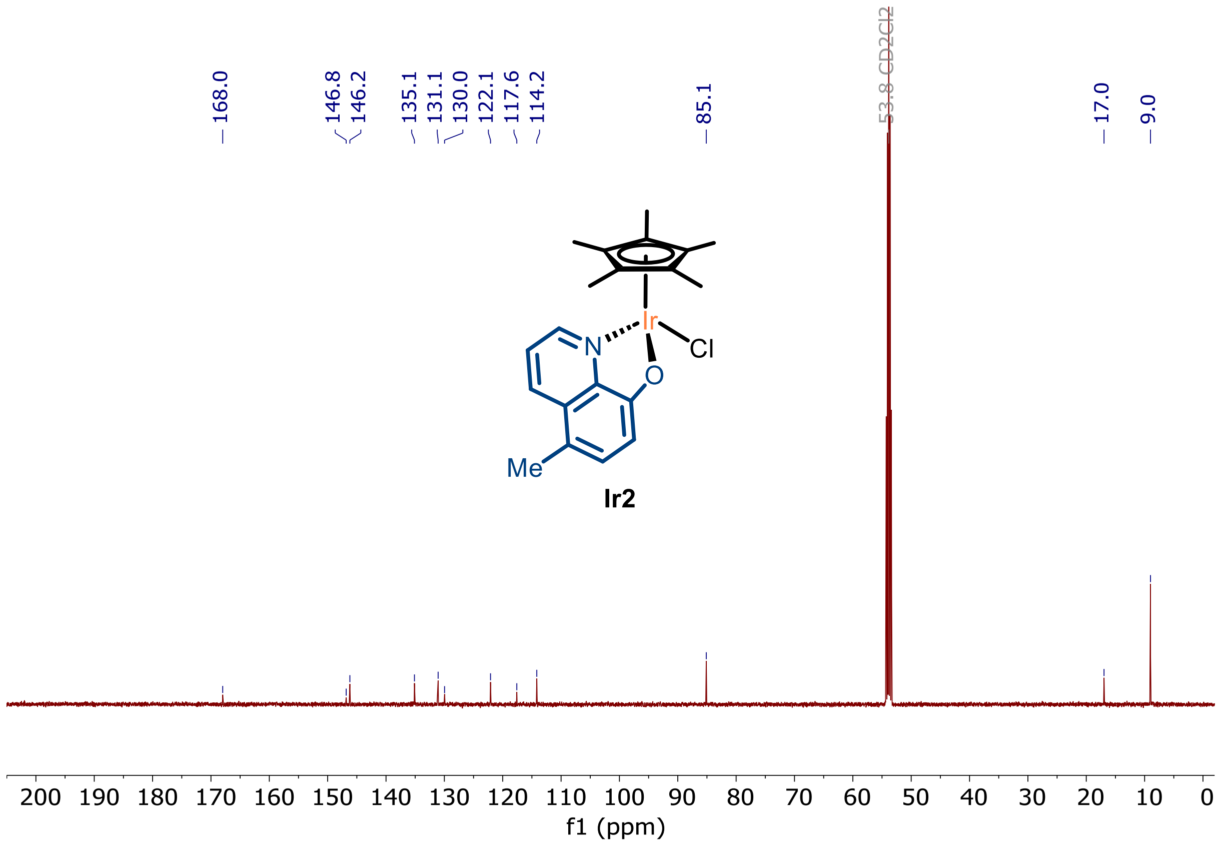


Figure S38. $^{13}C\{^1H\}$ NMR spectrum of **Ir2** in $CD_2Cl_2$ (126 MHz)

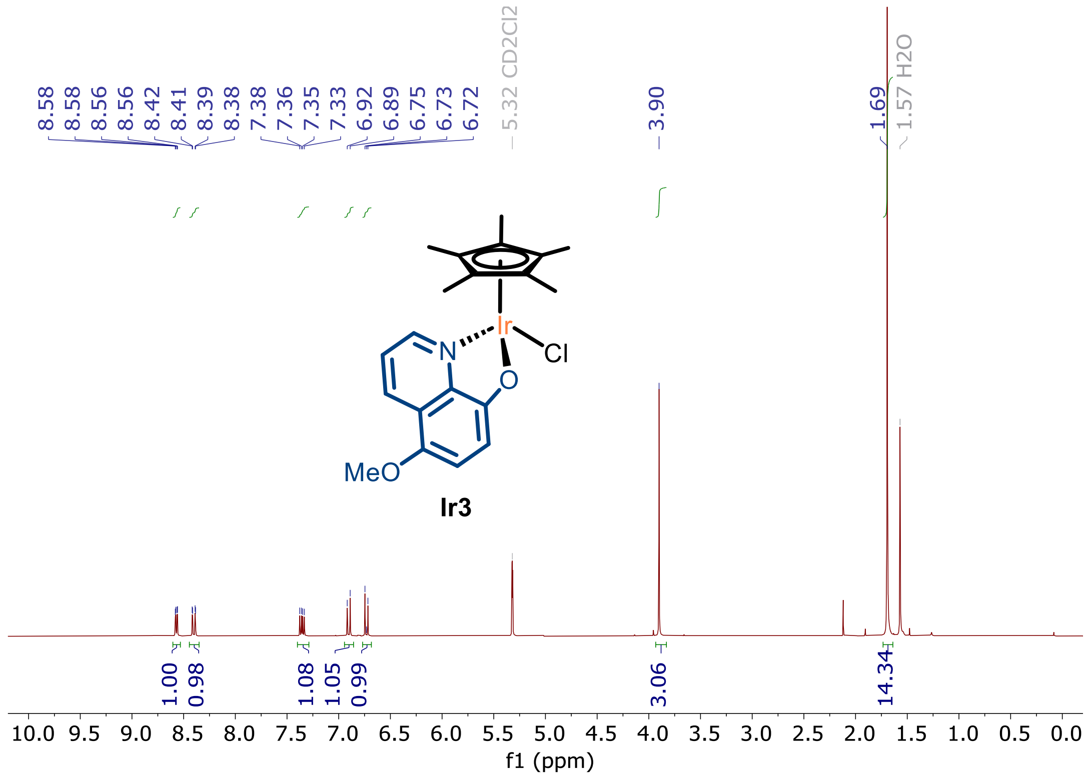


Figure S39. $^1H$ NMR spectrum of **Ir3** in $CD_2Cl_2$ (300 MHz)

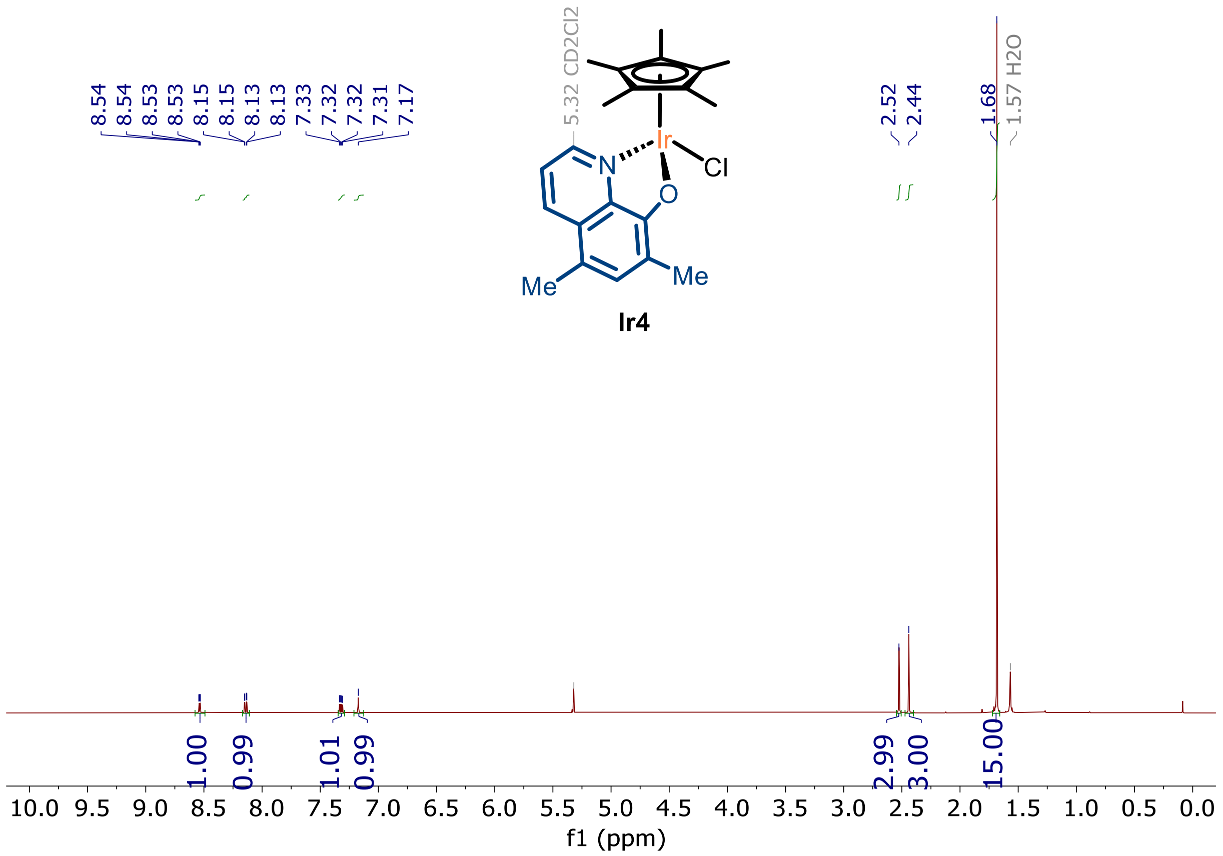


Figure S40. $^{1}$H NMR spectrum of **Ir4** in $CD_2Cl_2$ (500 MHz)

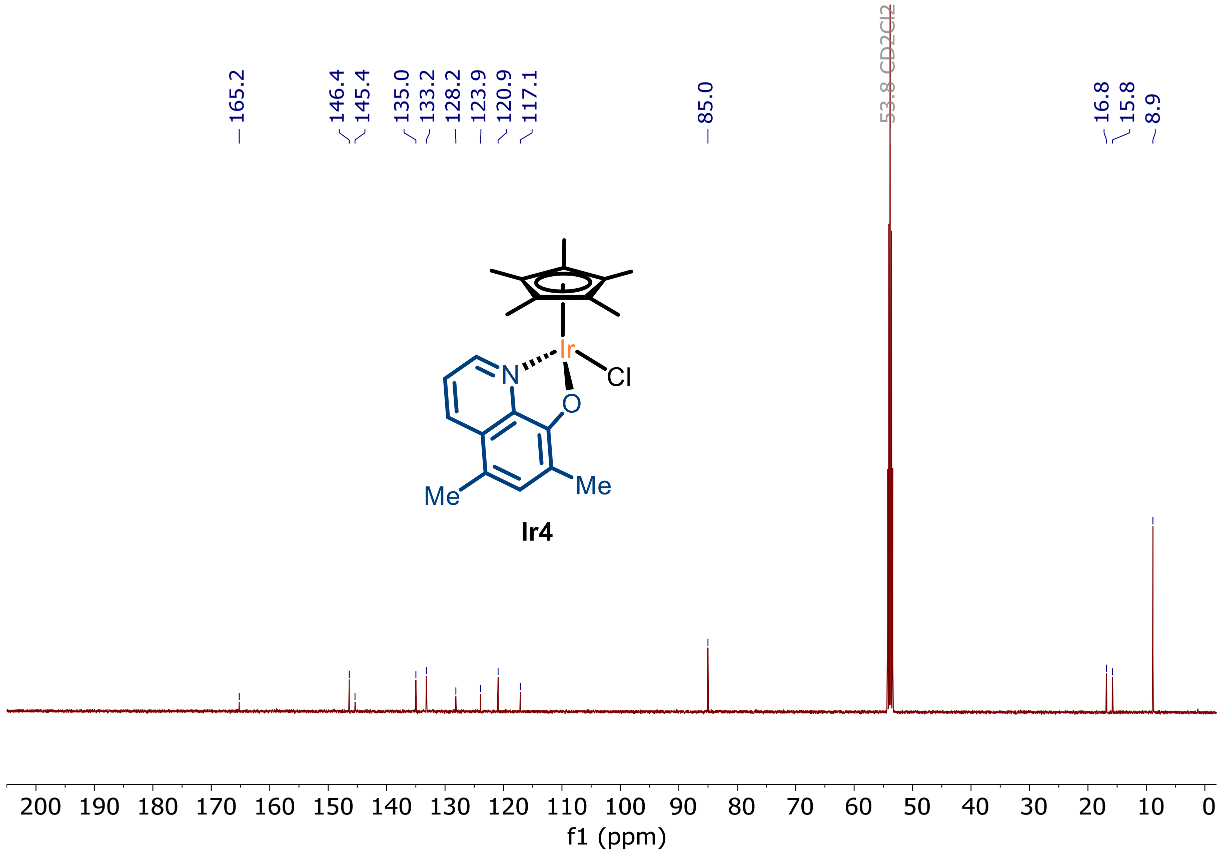

Figure S41. $^{13}C\{^{1}H\}$ NMR spectrum of **Ir4** in $CD_2Cl_2$ (126 MHz)

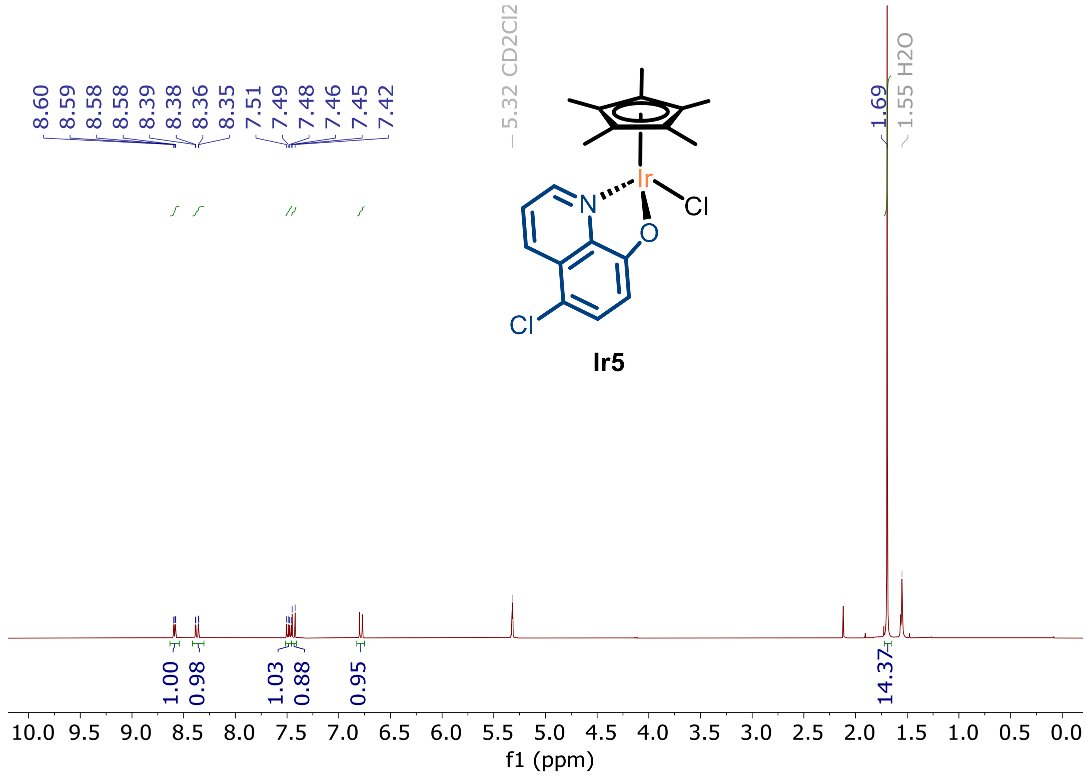


Figure S42. $^{1}H$ NMR spectrum of **Ir5** in $CD_2Cl_2$ (300 MHz)

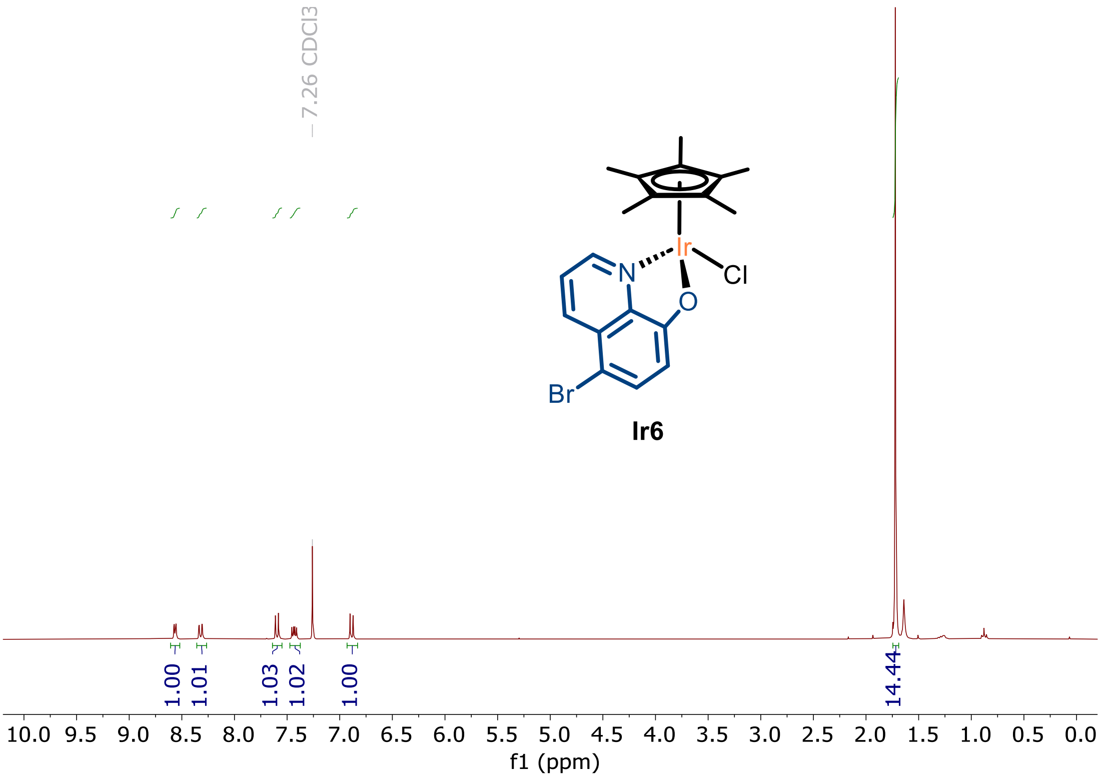


Figure S43. $^{1}H$ NMR spectrum of **Ir6** in $CDCl_3$ (300 MHz)

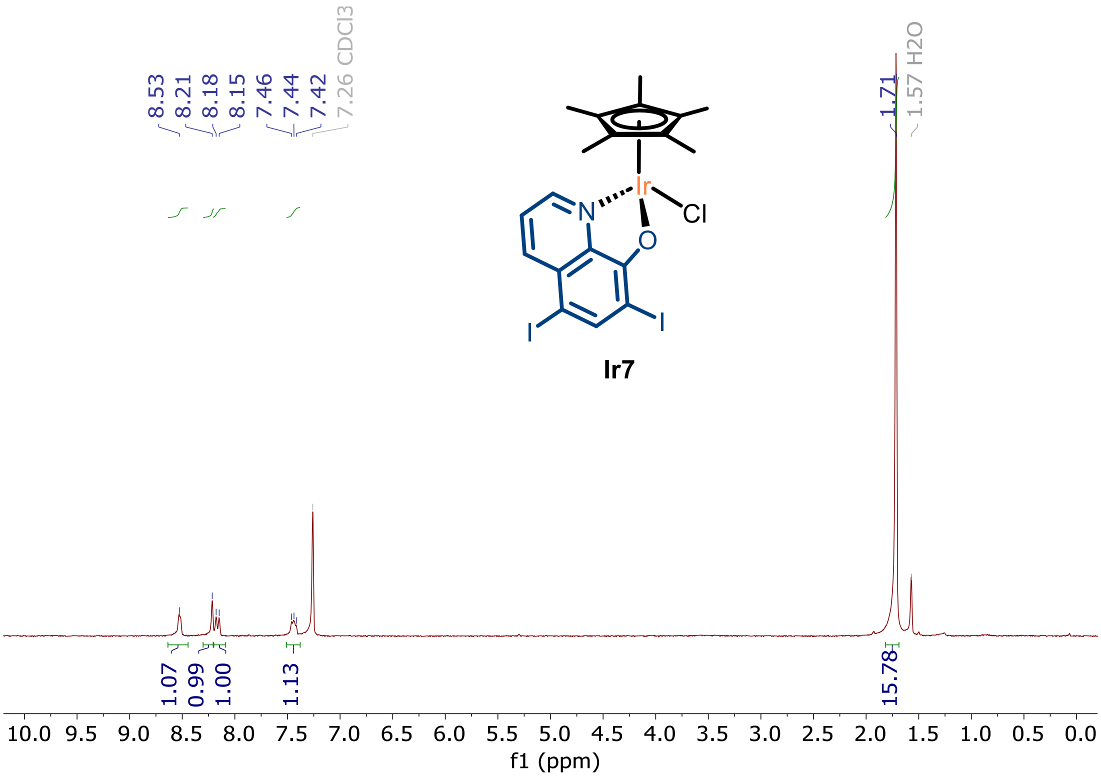


Figure S44. $^{1}$H NMR spectrum of **Ir7** in $CDCl_3$ (300 MHz)

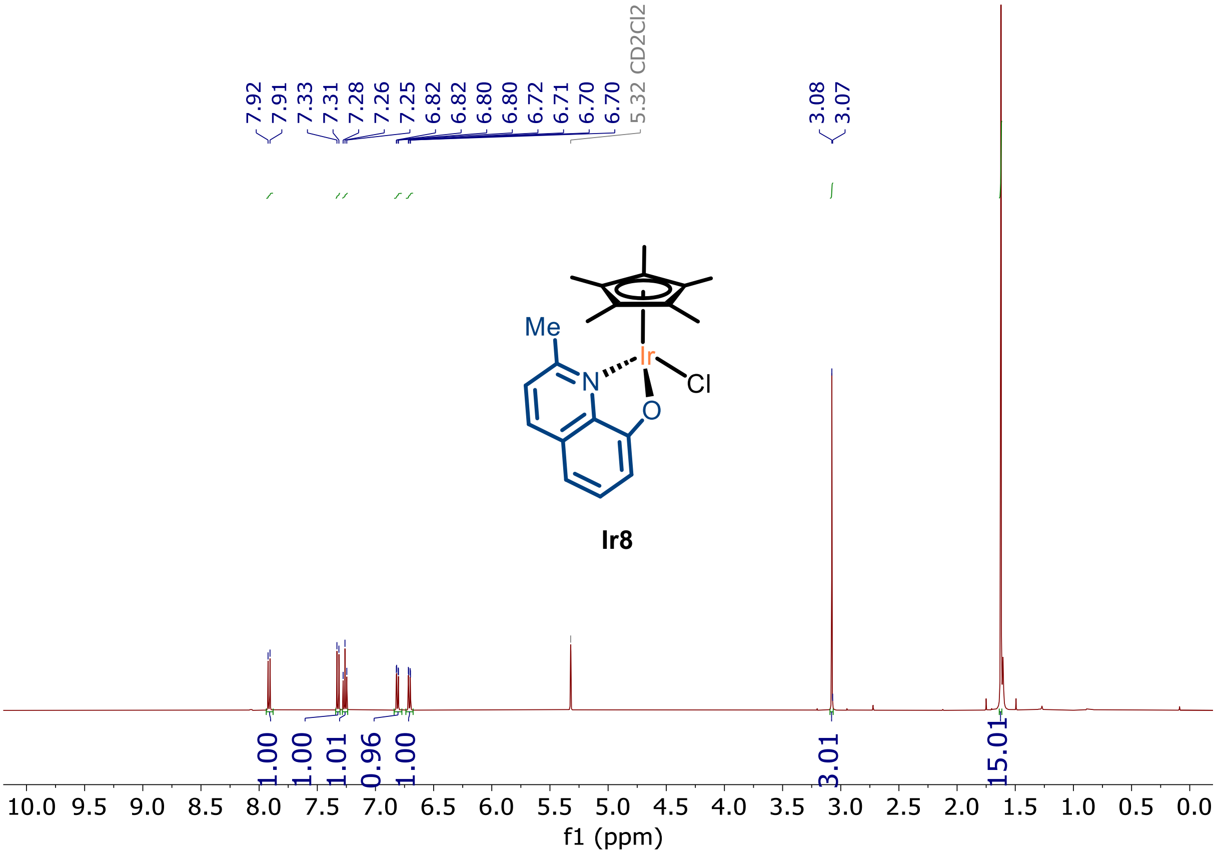


Figure S45. $^{1}$H NMR spectrum of **Ir8** in $CD_2Cl_2$ (500 MHz)

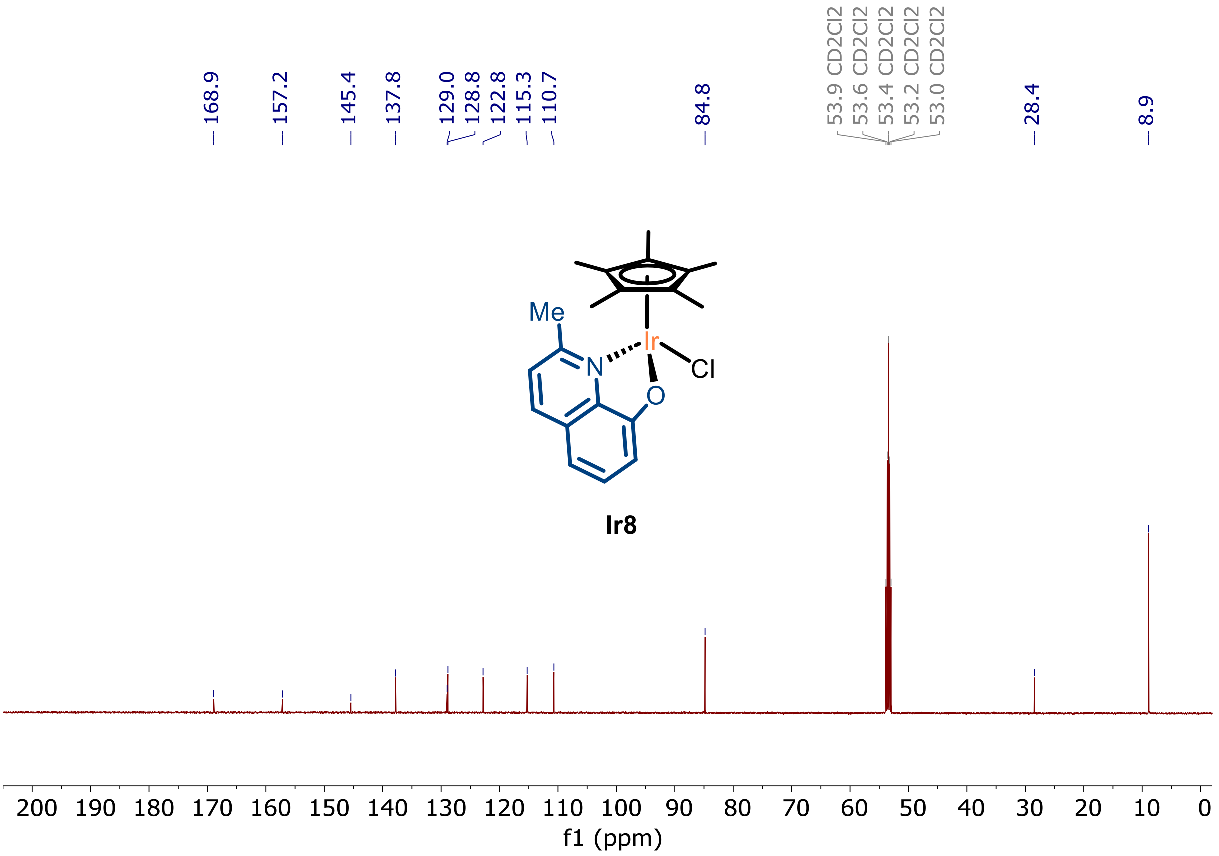


Figure S46. $^{13}C\{^1H\}$ NMR spectrum of **Ir8** in $CD_2Cl2$ (126 MHz)

## 13. Single Crystal X-Ray Diffraction of Ir2, Ir4 and Ir8

Table S4. Sample and crystal data for **Ir2**

| **Chemical formula** | **$C_{20}H_{23}ClIrNO$** | |
|---|---|---|
| **Formula weight** | 521.04 g/mol | |
| **Temperature** | 250(2) K | |
| **Wavelength** | 0.71073 Å | |
| **Crystal size** | 0.053 x 0.099 x 0.125 mm | |
| **Crystal habit** | clear intense orange prism | |
| **Crystal system** | monoclinic | |
| **Space group** | P 1 21/n 1 | |
| **Unit cell dimensions** | a = 8.1363(2) Å | α = 90° |
| | b = 17.7224(6) Å | β = 97.8770(10)° |
| | c = 12.7814(4) Å | γ = 90° |
| **Volume** | 1825.62(10) $Å^3$ | |
| **Z** | 4 | |
| **Density (calculated)** | 1.896 $g/cm^3$ | |
| **Absorption coefficient** | 7.466 $mm^{-1}$ | |
| **F(000)** | 1008 | |

Table S5. Data collection and structure refinement for **Ir2**.

| Theta range for data collection | 1.98 to 25.34° | |
|---|---|---|
| Index ranges | -9<=h<=9, -21<=k<=21, -15<=l<=15 | |
| Reflections collected | 59933 | |
| Independent reflections | 3337 [R(int) = 0.0734] | |
| Coverage of independent reflections | 99.9% | |
| Absorption correction | Multi-Scan | |
| Max. and min. transmission | 0.6930 and 0.4550 | |
| Structure solution technique | direct methods | |
| Structure solution program | XT, VERSION 2018/2 | |
| Refinement method | Full-matrix least-squares on $F^2$ | |
| Refinement program | SHELXL-2019/1 (Sheldrick, 2019) | |
| Function minimized | $\Sigma w(F_o^2 - F_c^2)^2$ | |
| Data / restraints / parameters | 3337 / 247 / 223 | |
| Goodness-of-fit on $F^2$ | 1.047 | |
| $\Delta/\sigma_{max}$ | 0.001 | |
| Final R indices | 2779 data; I>2σ(I) | R1 = 0.0261, wR2 = 0.0829 |
| | all data | R1 = 0.0398, wR2 = 0.1169 |
| Weighting scheme | $w=1/[\sigma^2(F_o^2)+(0.0742P)^2+7.1700P]$ where $P=(F_o^2+2F_c^2)/3$ | |
| Largest diff. peak and hole | 0.899 and -1.278 eÅ$^{-3}$ | |
| R.M.S. deviation from mean | 0.382 eÅ$^{-3}$ | |

Table S6. Sample and crystal data for **Ir4**

| Chemical formula | $C_{21}H_{25}ClIrNO$ | |
|---|---|---|
| Formula weight | 535.07 g/mol | |
| Temperature | 250(2) K | |
| Wavelength | 0.71073 Å | |
| Crystal size | 0.012 x 0.034 x 0.067 mm | |
| Crystal habit | clear intense orange prism | |
| Crystal system | orthorhombic | |
| Space group | P n a 21 | |
| Unit cell dimensions | a = 16.4890(6) Å | α = 90° |
| | b = 10.1064(3) Å | β = 90° |
| | c = 11.4265(4) Å | γ = 90° |
| Volume | 1904.16(11) Å$^3$ | |
| Z | 4 | |
| Density (calculated) | 1.866 g/cm$^3$ | |
| Absorption coefficient | 7.161 mm$^{-1}$ | |
| F(000) | 1040 | |

Table S7. Data collection and structure refinement for **Ir4**.

<table>
<tr><th>Theta range for data collection</th><th colspan="2">2.96 to 25.35°</th></tr>
<tr><td>Index ranges</td><td colspan="2">-19<=h<=13, -8<=k<=12, -13<=l<=13</td></tr>
<tr><td>Reflections collected</td><td colspan="2">9876</td></tr>
<tr><td>Independent reflections</td><td colspan="2">3472 [R(int) = 0.0503]</td></tr>
<tr><td>Coverage of independent reflections</td><td colspan="2">99.7%</td></tr>
<tr><td>Absorption correction</td><td colspan="2">Multi-Scan</td></tr>
<tr><td>Structure solution technique</td><td colspan="2">direct methods</td></tr>
<tr><td>Structure solution program</td><td colspan="2">XT, VERSION 2018/2</td></tr>
<tr><td>Refinement method</td><td colspan="2">Full-matrix least-squares on $F^2$</td></tr>
<tr><td>Refinement program</td><td colspan="2">SHELXL-2019/1 (Sheldrick, 2019)</td></tr>
<tr><td>Function minimized</td><td colspan="2">$\Sigma w(F_o^2 - F_c^2)^2$</td></tr>
<tr><td>Data / restraints / parameters</td><td colspan="2">3472 / 257 / 233</td></tr>
<tr><td>Goodness-of-fit on $F^2$</td><td colspan="2">1.005</td></tr>
<tr><td rowspan="2">Final R indices</td><td>2605 data; I>2σ(I)</td><td>R1 = 0.0376, wR2 = 0.0755</td></tr>
<tr><td>all data</td><td>R1 = 0.0610, wR2 = 0.0837</td></tr>
<tr><td>Weighting scheme</td><td colspan="2">$w=1/[\sigma^2(F_o^2)+(0.0352P)^2]$<br>where $P=(F_o^2+2F_c^2)/3$</td></tr>
<tr><td>Absolute structure parameter</td><td colspan="2">-0.016(13)</td></tr>
<tr><td>Largest diff. peak and hole</td><td colspan="2">0.654 and -0.977 eÅ$^{-3}$</td></tr>
<tr><td>R.M.S. deviation from mean</td><td colspan="2">0.146 eÅ$^{-3}$</td></tr>
</table>

Table S8. Crystal data and structure refinement for **Ir8**.

| Empirical formula | $C_{20}H_{23}ClIrNO$ |
|---|---|
| **Formula weight** | 521.04 |
| **Temperature/K** | 250.00(10) |
| **Crystal system** | monoclinic |
| **Space group** | $P2_1/c$ |
| **a/Å** | 7.51620(10) |
| **b/Å** | 29.5788(5) |
| **c/Å** | 16.6411(3) |
| **α/°** | 90 |
| **β/°** | 95.839(2) |
| **γ/°** | 90 |
| **Volume/Å$^3$** | 3680.46(10) |
| **Z** | 8 |
| **$\rho_{calc}$g/cm$^3$** | 1.881 |
| **μ/mm$^{-1}$** | 15.418 |
| **F(000)** | 2016.0 |
| **Crystal size/mm$^3$** | 0.105 × 0.02 × 0.015 |
| **Radiation** | Cu Kα (λ = 1.54184) |
| **2Θ range for data collection/°** | 5.976 to 136.47 |

| | |
|---|---|
| **Index ranges** | $-9 \le h \le 9$, $-35 \le k \le 12$, $-20 \le l \le 20$ |
| **Reflections collected** | 34505 |
| **Independent reflections** | 6714 [$R_{int}$ = 0.0387, $R_{sigma}$ = 0.0292] |
| **Data/restraints/parameters** | 6714/0/445 |
| **Goodness-of-fit on $F^2$** | 1.034 |
| **Final R indexes [I>=2σ (I)]** | $R_1$ = 0.0243, $wR_2$ = 0.0574 |
| **Final R indexes [all data]** | $R_1$ = 0.0307, $wR_2$ = 0.0597 |
| **Largest diff. peak/hole / e Å$^{-3}$** | 1.15/-0.58 |

## 14. References


(1) SAINT, Area-Detector Integration Program, Bruker-Nonius AXS, Madison, Wisconsin, USA, 2004 (+ v7.12a)

(2) G. M. Sheldrick, SADABS Version 2004/1. A Program for Empirical Absorption Correction, University of Göttingen, Germany, 2004.

(3) SHELXTL-NT version 6.12, Structure Determination Package, Bruker-Nonius AXS, Madison, Wisconsin, USA, 2001

(4) Hong, S. Y.; Park, Y.; Hwang, Y.; Kim, Y. B.; Baik, M.-H.; Chang, S., Selective formation of γ-lactams via C–H amidation enabled by tailored iridium catalysts. *Science* **2018,** *359*, 1016-1021.

(5) Hong, S. Y.; Chang, S., Stereodefined Access to Lactams via Olefin Difunctionalization: Iridium Nitrenoids as a Motif of LUMO-Controlled Dipoles. *J. Am. Chem. Soc.* **2019,** *141*, 10399-10408.

(6) Lee, M.; Jung, H.; Kim, D.; Park, J.-W.; Chang, S., Modular Tuning of Electrophilic Reactivity of Iridium Nitrenoids for the Intermolecular Selective α-Amidation of β-Keto Esters. *J. Am. Chem. Soc.* **2020,** *142*, 11999-12004.

(7) Hwang, Y.; Jung, H.; Lee, E.; Kim, D.; Chang, S., Quantitative Analysis on Two-Point Ligand Modulation of Iridium Catalysts for Chemodivergent C–H Amidation. *J. Am. Chem. Soc.* **2020,** *142*, 8880-8889.

(8) Dong, D.-J.; Li, H.-H.; Tian, S.-K., A Highly Tunable Stereoselective Olefination of Semistabilized Triphenylphosphonium Ylides with N-Sulfonyl Imines. *J. Am. Chem. Soc.* **2010,** *132*, 5018-5020.

(9) Zimmerman, H. E.; Pushechnikov, A., The Stereochemistry of Allenic Enol Tautomerism – Independent Generation and Reactivity of the Enolates. *Eur. J. Org. Chem.* **2006,** *2006*, 3491-3497.

(10) Alshakova, I. D.; Foy, H. C.; Dudding, T.; Nikonov, G. I., Ligand Effect in Alkali-Metal-Catalyzed Transfer Hydrogenation of Ketones. *Chem. Eur. J.* **2019,** *25*, 11734-11744.

(11) Wang, J.; Peng, Y.; Xu, J.; Wu, Q., Deracemization of racemic alcohols combining photooxidation and biocatalytic reduction. *Org. Biomol. Chem.* **2022,** *20*, 7765-7769.
